# Protein Kinase PoxMKK1 Regulates Plant-Polysaccharide-Degrading Enzyme Biosynthesis, Mycelial Growth and Conidiation in *Penicillium oxalicum*

**DOI:** 10.3390/jof9040397

**Published:** 2023-03-23

**Authors:** Bo Ma, Xue-Mei Luo, Shuai Zhao, Jia-Xun Feng

**Affiliations:** State Key Laboratory for Conservation and Utilization of Subtropical Agro-Bioresources, Guangxi Research Center for Microbial and Enzyme Engineering Technology, College of Life Science and Technology, Guangxi University, Nanning 530004, China

**Keywords:** *Penicillium oxalicum*, PoxMKK1, plant-polysaccharide-degrading enzyme, mycelial development

## Abstract

The ability to adapt to changing environmental conditions is crucial for living organisms, as it enables them to successfully compete in natural niches, a process which generally depends upon protein phosphorylation-mediated signaling transduction. In the present study, protein kinase PoxMKK1, an ortholog of mitogen-activated protein kinase kinase Ste7 in *Saccharomyces cerevisiae*, was identified and characterized in the filamentous fungus *Penicillium oxalicum*. Deletion of *PoxMKK1* in *P. oxalicum* Δ*PoxKu70* led the fungus to lose 64.4–88.6% and 38.0–86.1% of its plant-polysaccharide-degrading enzyme (PPDE) production on day 4 after a shift under submerged- and solid-state fermentation, respectively, compared with the control strain Δ*PoxKu70*. In addition, PoxMKK1 affected hypha growth and sporulation, though this was dependent on culture formats and carbon sources. Comparative transcriptomics and real-time quantitative reverse transcription PCR assay revealed that PoxMKK1 activated the expression of genes encoding major PPDEs, known regulatory genes (i.e., *PoxClrB* and *PoxCxrB*) and cellodextrin transporter genes (i.e., *PoxCdtD* and *PoxCdtC*), while it inhibited the essential conidiation-regulating genes, including *PoxBrlA*, *PoxAbaA* and *PoxFlbD*. Notably, regulons modulated by PoxMKK1 and its downstream mitogen-activated protein kinase PoxMK1 co-shared 611 differential expression genes, including 29 PPDE genes, 23 regulatory genes, and 16 sugar-transporter genes. Collectively, these data broaden our insights into the diverse functions of Ste7-like protein kinase, especially regulation of PPDE biosynthesis, in filamentous fungi.

## 1. Introduction

Plant biomass is one of the most abundant and sustainable polymeric substrates used for the production of renewable bioenergy and commodity biochemicals, and it is comprised primarily of lignocellulose, which is rich in polysaccharides (such as cellulose and hemicellulose) and lignin (i.e., complex aromatic polymer) [1]. In the recent decades, the yields of renewable bio-based products from plant biomass-derived sugars have increased exponentially [2]. However, the often-inefficient saccharification of plant biomass still represents a key industrial bottleneck, largely resulting from its natural recalcitrance [3].

Fungi are widespread in a variety of natural environments and can exploit a wide range of carbon sources. They play a crucial function in the global carbon cycle because of their ability to break down plant biomass by secreting plant-polysaccharide-degrading enzyme (PPDE) [1,4]. Filamentous fungi are excellent producers of PPDE, where the expression of PPDE genes is finely regulated at both the transcriptional and post-translational levels [5]. Protein phosphorylation is the most common form of post-translational modification, functioning in many biological processes [6].

Many signals are transmitted by the mitogen-activated protein (MAP) kinase pathway, which is evolutionarily conserved and found ubiquitously from yeast to mammals [7]. The core elements of the MAP kinase (MAPK) pathway consist of three-tiered cascade kinases, termed MAPKKK (MAP kinase kinase kinase), MAPKK (MAP kinase kinase) and MAPK (MAP kinase), which are sequentially activated by a phosphorylation cascade, which is in turn initiated by the sensing of environmental stimuli using its upstream two-component signal transduction system or G-proteins-coupled receptors [7,8]. Five MAPK pathways have been identified in *Saccharomyces cerevisiae*, while only three of them have been found in many filamentous fungi [9].

The pheromone response/filamentation pathway, mediated by Ste11–Ste7–Fus3/Kss1, is the most extensively studied, and it is responsible for the regulation of fungal development, the production of secondary metabolites, and even pathogenicity in filamentous fungi [10]. However, it exhibits diversity in its regulatory functions in homologues and/or different components of the signaling pathways of filamentous fungus. For instance, deletion of the orthologous gene of yeast, *Ste11*, resulted in conidiation defects in *Cochliobolus heterostrophus* [11] but produced more spores in *Ashbya gossypii* [12]. The loss of *AbSte7* from *Alternaria brassicicola*, caused notably faster growth on potato dextrose agar (PDA) containing sorbitol [13], whereas a lack of Ste7-like kinase Mkk6 in *Beauveria bassian*a presented inconspicuous changes in sensitivity to sorbitol [14]. In *Ustilaginoidea virens*, *UvPmk1* knockout demonstrated an increase in tolerance to cell wall stresses [15], while, conversely, *CcPmk1* deletion led to hypersensitivity to cell wall inhibitors in *Cytospora chrysosperma* [16]. Furthermore, in the plant pathogenic fungus *Colletotrichum gloeosporioides*, CgSte50, CgSte11, CgSte7, and CgMK1 were required for appressorium formation, penetration of the cellophane membrane, invasive growth, and pathogenicity, and also affected vegetative growth under nitrogen limitation conditions. Notably, CgSte50, CgSte11, and CgSte7, but not CgMk1, played important roles in the oxidative stress response [17]. However, the function of Ste7 homologues has not been investigated in detail in industrial filamentous fungus.

*Penicillium oxalicum* exhibits promise for application in ecological reconstruction, drug production, agricultural biocontrol, and biorefinery [18,19,20]. Meanwhile, as a fast-growing saprophytic fungus species, *P. oxalicum* can secrete various lignocellulolytic enzymes, and has potential for the production of industrial-scale PPDEs. Although many transcription factors (TFs) that regulate the expression of PPDE genes have been identified, few reports have been published regarding the signal transduction cascade in *P. oxalicum*. Heterotrimeric G protein, as an important component of the cell signaling cascade that transduces receptor signals to the intracellular environment, is involved in the regulation of PPDE production in *P. oxalicum*. For instance, Gα subunit 3 mediates the G protein-cAMP signaling pathway in order to transduce various carbon source signals, and positively regulates the expression of TF-encoding gene *amyR*, subsequently affecting amylase and cellulase production [21]. The Gγ protein modulates PPDE production by mediating the expression of the regulatory gene *PoxCxrB*, a gene which is required for the expression of the major cellulase and xylanase genes. PoxCxrB also indirectly regulates the mRNA levels of major amylase genes by controlling the expression of *amyR* [22]. Recently, we found that the terminal component MAP kinase, PoxMK1, of the pathway positively regulated the expression of major PPDE genes and known essential regulatory genes—e.g., *PoxClrB* and *PoxCxrB*—in *P. oxalicum* [23].

In this work, PoxMK1-interacting proteins were screened by yeast-two hybrid assay (Y2H), and Ste7-homologous PoxMKK1 (POX07948) was selected for further study. We characterized its roles in the regulation of PPDE production, vegetative growth and conidiation in *P. oxalicum* under both submerged (SmF) and solid-state (SSF) fermentation conditions, respectively.

## 2. Materials and Methods

### 2.1. Strains, Media and Growth Conditions

*P. oxalicum* HP7-1 (#10781; China General Microbiological Culture Collection, CGMCC), a wild-type strain isolated from the forest soil of Huaping National Natural Reserve in Guilin, Guangxi, China, was reported previously [24]. In order to improve gene targeting frequency, *POX01583*, encoding the homologue of *Ku70* involved in the non-homologous end-joining pathway, was replaced in the wild-type HP7-1 by a hygromycin resistance gene via homologous recombination techniques [24]. The strain Δ*PoxKu70* (#3.15650; CGMCC) was used as a background strain for the construction of deletion mutants, and then the mutant was used to construct the corresponding complementation strain. All *P. oxalicum* strains were maintained on potato dextrose agarose (PDA, Difco^®^ Laboratories, Le Pont de Claix, Auvergne-Rhône-Alpes, France) for 6 days at 28 °C in order to collect asexual conidia through the use of sterile distilled H_2_O supplemented with 0.1% (*w*/*v*) Tween-80 (Sangon, Shanghai, China). The harvested spores were stored in 25% (*v*/*v*) glycerol at −80 °C for a long time.

For the determination of PPDE production, RNA sequencing, and real-time quantitative reverse transcription PCR (RT-qPCR), *P. oxalicum* conidia (1.0 × 10^8^) were inoculated into 100 mL modified minimum medium [MMM, per liter: KH_2_PO_4_ 4.0 g, (NH_4_)_2_SO_4_ 4.0 g, MgSO_4_·7H_2_O 0.6 g, CaCl_2_ 0.6 g, 1.0 mL Tween-80; FeSO_4_·7H_2_O 0.005 g, MnSO_4_ 0.0016 g, ZnCl_2_ 0.0017 g and CoCl_2_ 0.002 g; pH 5.0] with 1.0% (*w*/*v*) D-glucose and cultivated for 24 h at 28 °C with shaking at 180 rpm [25]. Mycelia were harvested and transferred to MMM containing 2.0% Avicel (Avicel-PH101, Sigma-Aldrich, St. Louis, MO, USA), 1.0% soluble corn starch (SCS, Sigma-Aldrich, St. Louis, MO, USA) or wheat bran plus rice straw (WR), and the inoculated media were incubated for 2–4 days at 28 °C for enzyme activity measurement or for 4–48 h for RNA sequencing and RT-qPCR assay, respectively.

For assay of radial growth, five microliters of conidial suspension (approximately 0.5 × 10^5^ spores) were dropped onto solid plates containing PDA, complete medium [CM, per liter: 10.0 g D-glucose, 2.0 g peptone, 1.0 g yeast extract, 1.0 g acid-hydrolysed casein (Sangon, Shanghai, China), 6.0 g NaNO_3_, 0.52 g KCl, 0.52 g MgSO_4_·7H_2_O, 1.52 g KH_2_PO_4_; pH 6.5] and MMM containing 1.0% glucose, 1.0% SCS or 2.0% Avicel. The inoculated plates were incubated at 28 °C. For the evaluation of submerged growth, equivalent spores were inoculated in 100 mL liquid media of CM and MMM supplemented with 1.0 g glucose, 1.0 g SCS, and 2.0 g Avicel, respectively, and were then cultivated for 72 h at 28 °C with shaking at 180 rpm.

### 2.2. Yeast-Two Hybrid Assay

In order to construct plasmids for Y2H analysis, the full-length sequence of *PoxMK1* cDNA digested by the restriction enzymes *Eco*RI and *Bam*HI was subcloned into the corresponding sites of pGBKT7 plasmid, as the bait, and the cDNA of candidate gene was subcloned into the *Eco*RI sites of pGADT7 plasmid, as the prey. All the cDNAs were amplified by PCR with special primer pairs (Supplementary Table S1), followed by verification through DNA sequencing. The verified bait plasmids were transformed into Y2H GOLD yeast-competent cells in order to test for toxicity and auto-activation activity on SD/-Trp plates using the lithium acetate/PEG protocol, and the bait and prey with candidate gene was co-transformed in to Y2H GOLD cells and placed on SD/-Leu/-Trp (DDO) plates. The transformant was verified by PCR amplification with special primer pairs (Supplementary Table S1) and then inoculated into liquid DDO medium cultivated for 1 day at 28 °C with shaking at 200 rpm. Ten microliters of tenfold serial dilution cells were grown on QDO plates with 150 mg/mL AbA and 20 ng/mL X-α-gal at 30 °C for 5 days. The experiment was performed twice independently.

### 2.3. Construction of Gene Deletion Mutant and Its Complementary Strain

For the generation of deletion mutant of each gene *POX04853*, *POX06496*, *POX07588*, and *POX07948*, the knockout cassette, containing 5′ and 3′ DNA fragments of the target gene and the G418-resistance gene fragment, was constructed by fusion PCR with specific primer pairs (Supplementary Table S1) according to previously published protocols [25]. Subsequently, the knockout cassette was directly transformed into fresh protoplasts of strain Δ*PoxKu70*, the transformants were selected on G418-containing PDA plates, and then knockout candidates were verified by PCR amplification using the special primer pairs (Supplementary Table S1). Similarly, the deletion mutant of *PoxMK1* could also be obtained by the above method [23].

For the creation of a complementary strain of mutant Δ*POXO7948* (Δ*PoxMKK1*)-, the complementary cassette was used to replace the protease gene *PoxPepA*, composed of the complete coding region of *PoxMKK1*, its native promoter and terminator, the bleomycin resistance gene fragment, and the upstream- and downstream-flanking DNA sequence of *PoxPepA*. The transformants were screened on PDA plate containing 80 μg/mL bleomycin (Sigma-Aldrich, Darmstadt, Germany) and was validated by the special primer pairs (Supplementary Table S1), as previously described by Yan et al. [26].

### 2.4. Molecular Manipulation

For total DNA extraction, *P. oxalicum* spores (~10^8^) were transferred into liquid CM at 28 °C in a rotatory shaker (180 rpm) for 24 h. Subsequently, vegetative mycelia were collected through vacuum filtration and used for extraction of total DNA by following the modified phenol-chloroform method [24]. The genomic DNA was digested with *Xho*I (TaKaRa Bio Inc. Dalian, China) and separated from 0.75% agarose gel electrophoresis. Subsequently, the DNA was transferred onto the Hybond-N^+^ nylon membranes (GE Healthcare Limited, Amersham, UK). Detection of the probe-hybridized DNA fragment was carried out using the DIG High Prime DNA Labeling and Detection Starter Kit I following the manufacturer’s protocol (Roche Diagnostics, Mannheim, Germany). The probe was amplified by special primers sPOX07948-F/sPOX07948-R for Southern hybridization (Supplementary Table S1).

For total RNA extraction, mycelia were harvested and separated by filtering the culture with an eight-layer filter fabric, after which they were washed three times using diethyl pyrocarbonate-treated water prior to RNA extraction. Total RNA isolation was performed using Trizol reagent (Invitrogen, Carlsbad, CA, USA) according to the manufacturer’s instructions.

### 2.5. RNA Sequencing and RT-qPCR Analysis

For RNA sequencing, total RNA was extracted from the mycelia of *P. oxalicum* strains (Δ*PoxMKK1* and Δ*PoxKu70*) exposed on Avicel for 24 h, treated with DNase I and purified after mRNA enrichment using oligo (dT) according to the manufacturer’s guidelines. mRNA sequencing was carried out on a BGISEQ-500 platform at BGI (Shenzhen, China). The sequenced data were processed as described by Yan et al. [26]. Values in terms of Fragments per kilobase of exon per million mapped reads (FPKM) represented gene transcriptional levels, and differentially expressed genes (DEGs) were screened with a standard of probability ≥ 0.8 and |log2 (Δ*PoxMKK1*_FPKM/Δ*PoxKu70*_FPKM)| ≥ 1. Similarly, DEGs between Δ*PoxMK1* and Δ*PoxKu70* were also selected with the same standard and were described in detail in Reference [23].

For comprehensive deciphering of the transcription profiling of Δ*PoxMKK1* in response to Avicel, the DEGs were annotated according to the gene ontology (GO) knowledge base. DEGs were enriched and functionally classified by Blast2GO program with Fisher’s exact test in association with a false discovery rate (FDR) correction for multiple testing (FDR < 0.05) [27].

The RT-qPCR assay was conducted on an ABI 7500 Real-System (Thermo Fisher Scientific, Waltham, MA, USA). The Bio-Rad iQ5 software was used to compile PCR protocols and to define the plate setup. The PCR reaction mixture and program were referred to in the previous report [23]. The primer sequences used were listed in Supplementary Table S1. LightCycler480 software 1.5.0 was used to calculate the Ct value. The transcription levels of target genes were normalized against the level of actin gene (*POX09248*), as the endogenous reference gene, with 2^−ΔΔCt^ relative quantification method [28]. All samples were analyzed in three independent experiments with three replicates.

### 2.6. Determination of Enzyme Activity and Protein Concentration

The preparation of crude enzyme solution and the determination of PPDE activities were implemented as previously described [26,29]. For crude enzyme solution preparation under SmF conditions, the culture was centrifuged at 11,300× *g* for 10 min at 4 °C, and the supernatant was stored at 4 °C for further analysis of enzyme activity. Under SSF conditions, the cultivated solid medium was added to 200 mL sterile ddH_2_O and crushed by a glass rod, then the mixture was shaken at 180 rpm for 2 h at 28 °C before being squeezed and centrifuged at 11,300× *g* for 20 min. The supernatant was used as a crude enzyme solution.

In order to measure PPDE activity, filer paper cellulase (FPase), carboxy methyl cellulase (CMCase), soluble starch-degrading enzyme (SSDE), raw starch-degrading enzyme (RSDE) and xylanase were assayed against Whatman No. 1 filter paper (1.0 cm × 6.0 cm; GE Healthcare Limited, Little Chalfont, Buckinghamshire, UK), CMC-Na (Sigma-Aldrich, Darmstadt, Germany), SCS (Sigma-Aldrich, Darmstadt, Germany), raw cassava starch (self-preparation) and xylan from beechwood (Megazyme International Ireland, Wicklow, Ireland) using a DNS method, respectively. The activities of *p*-nitrophenyl-β-cellobiosidase (pNPCase) and *p*-nitrophenyl-β-glucopyranosidase (pNPGase) were evaluated against *p*-nitrophenyl-β-D-cellobioside (pNPC) and *p*-nitrophenyl-β-D-glucopyranoside (pNPG) (Sigma-Aldrich, Darmstadt, Germany), respectively. The activity unit (U) was defined as the amount of enzyme required to produce one mol of reducing sugar or *p*-nitrophenyl per minute from the reaction substrates.

In addition, the protein concentration of mycelia cells was determined using the Detergent Compatible Bradford Assay Kit (Pierce Biotechnology, Rockford, IL, USA) against a BSA standard.

### 2.7. Phenotypic and Growth Analyses

For radial growth assay, the diameters of colonies found on different media cultured for 5 days were determined through two measurements taken perpendicular to each other across the center as indices of radial growth rates, and a Canon EOS 600D (Canon, Japan) was used for photography. For liquid growth assay, mycelia were collected by vacuum filtration at 12 h intervals in liquid CM and MMM with 1.0% (*w*/*v*) glucose or SCS and were then washed three times with deionized water. The mycelia were dried at 50 °C to a constant weight. However, the growth was indirectly measured by the amount of intracellular protein in liquid MMM with 2.0% (*w*/*v*) Avicel, as described above. All of the tests were repeated three times. Furthermore, the hypha of *P. oxalicum* in liquid medium were observed on day 2 after inoculation under a light microscope (OLYMPUS DP480, Olympus, Tokyo, Japan), and the photomicrographs were taken and analyzed by Olympus cellSens Dimension digital imaging software (Version 1.14).

### 2.8. Quantification of Conidia Production

In order to investigate the effect of PoxMKK1 on conidiation in *P. oxalicum*, the spores were counted using a hemocytometer on solid plates and in liquid culture, respectively. In the case of spores on solid plate, conidia were washed twice by 5.0 mL sterile ddH_2_O with 0.1% Tween-80, and the conidial suspension was mixed thoroughly and filtered through two layers of sterile gauze. Subsequently, the collected conidia were washed and ultimately re-suspended in ddH_2_O. The conidia production was quantified with the number of conidia per unit of colony area. For spores in the liquid culture, the culture was filtered through sterile gauze in order to remove the hyphae and medium residues, and the collected filtrate was diluted appropriately and used for conidia counting. Conidia production was indicated by the number of conidia per milliliter. Three biological replicates were performed for each strain.

### 2.9. Protein Sequence Analysis

The Simple Modular Architecture Research Tool (SMART, http://smart.emblheidelberg.de/ (accessed on 6 July 2020)) and InterPro online (http://www.ebi.ac.uk/interpro/ (accessed on 8 July 2020)) were used for prediction of the conserved domains contained in the PoxMKK1. The homologous sequences of MAP kinases from different fungi were searched with BlastP and downloaded from GenBank. Multiple sequence alignment was carried out using ClustalX 2.0 [30], and the phylogenetic tree was constructed by MEGA-X using the neighbor-joining method [31].

### 2.10. Accession Numbers

The transcriptomic data of the *P. oxalicum* strains have been loaded into the Sequence Read Archive database (accession No. GSE154710). In addition, the sequence of PoxMKK1 was submitted to GenBank with accession number MT468562.

## 3. Results

### 3.1. POX07948 Interacting with PoxMK1 Is Required for FPase Production in P. oxalicum

A previous work found that MAP kinase PoxMK1 modulated the production of PPDE in *P. oxalicum* under both SmF and SSF conditions. Here, the Y2H was used to screen for PoxMK1-interacting proteins in *P. oxalicum*. The full-length PoxMK1 was used as bait, and the differentially phosphorylated TFs and kinases reported previously as a result of *PoxMK1* deletion [23] were used as prey. A total of four PoxMK1-interacting proteins (POX04853, POX06496, POX07588, and POX07948) were identified (Supplementary Figure S1).

In order to test whether these identified PoxMK1-interacting proteins were involved in the cellulase production of *P. oxalicum*, all of them were deleted in the control strain Δ*PoxKu70* in order to generate the corresponding mutants Δ*POX04853*, Δ*POX06496*, Δ*POX07588*, and Δ*POX07948*, which were validated by PCR (Supplementary Figure S2A) with specific primers (Supplementary Table S1). When cultured in MMM containing 2.0% Avicel as the sole carbon source for 2–4 days after a shift from glucose, mutant Δ*POX06496* showed 32.3–55.0% increased FPase production as compared with the Δ*PoxKu70*, whereas mutant Δ*POX07948* lost 41.2–92.9% of its FPase production. In contrast, mutants Δ*POX04853* and Δ*POX07588* exhibited similar FPase production to Δ*PoxKu70* (Supplementary Figure S3). These data displayed that POX07948 positively participated in the regulation of cellulase production, and it was therefore selected for further study.

Southern hybridization analysis was performed with a special probe (Supplementary Table S1) in order to further determine whether the single copy of the *POX07948* deletion cassette was integrated into the right site of the Δ*PoxKu70* genome (Supplementary Figure S2B). The complementary strain C*POX07948* was generated by introducing the complementary cassette to deletion mutant Δ*POX07948* as described previously [26], and the bleomycin-resistant transformants were isolated and verified by PCR with special primers (Supplementary Table S1). The expected size bands were amplified, as shown in Supplementary Figure S4.

### 3.2. Characterization and Phylogenetic Analyses of PoxMKK1 in P. oxalicum

**Figure 1:**
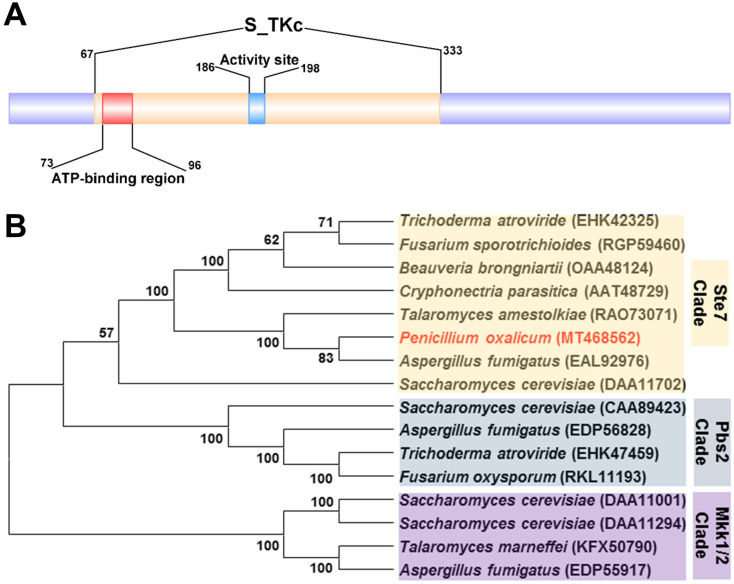
Structure and phylogenetic analyses of protein kinase PoxMKK1 (POX07948). (**A**) Conserved domain. S_TKc, serine/threonine protein kinase catalytic domain. (**B**) Phylogenetic tree of PoxMKK1 and its homologs. The phylogenetic tree was generated by the MEGA X software using the neighbor-joining (N–J) method. The numbers at the branch nodes are bootstrap values (>50%) based on 1000 replicates.

The POX07948 protein was composed of 557 amino acids, encoded by gene *POX07948* with a length of 1925 bp, containing three introns, according to the genome annotation of *P. oxalicum* strain HP7-1 [20]. The Simple Modular Architecture Research Tool (SMART) and InterPro online analyses indicated that the POX07948 protein contained a conserved serine/threonine protein kinase catalytic (S_TKc) domain from residues 67–333, whose ATP-binding region and protein kinase activity site were located at residues 73–96 and 186–198, respectively (Figure 1A). An NCBI BlastP search revealed that the POX07948 protein shared 100%, 78.30%, 67.66%, and 44.33% of its identity with its homologous proteins from *P. oxalicum* 114-2 (GenBank accession No. EPS26612), *Aspergillus fumigatus* Af293 (EAL92976), *Talaromyces amestolkiae* CIB (RAO73071), and *S. cerevisiae* S228C (DAA11702), respectively. Moreover, phylogenetic analysis showed that the POX07948 protein was resolved in the Ste7 clade and visibly separated from the Pbs2 and Mkk1/2 clades (Figure 1B). In order to handily study further, POX07948 was denominated as PoxMKK1.

### 3.3. PoxMKK1 Is Involved in Mycelial Growth and Conidiation in P. oxalicum

**Figure 2:**
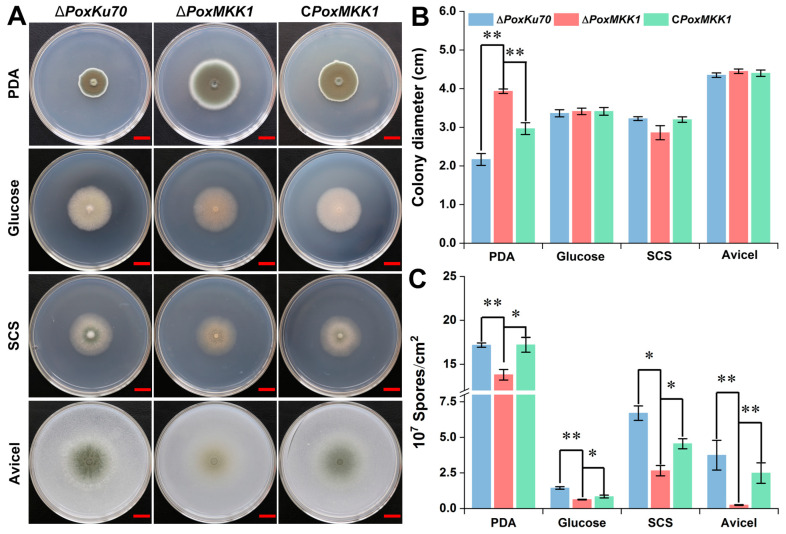
Phenotype characteristics of *P. oxalicum* mutant Δ*PoxMKK1*, the control strain Δ*PoxKu70*, and complementary strain C*PoxMKK1* on the different solid plates. (**A**) Colony morphology grown for 4 days. Scale bars = 1.0 cm. (**B**) Colony diameter. (**C**) Spore count. The number of spores was counted on PDA at 28 °C for 5 days, on MMM with 1.0% glucose and 1.0% SCS for 14 days, and on 2.0% Avicel for 7 days, respectively. PDA, potato dextrose agarose. MMM, modified minimum medium. SCS, soluble corn starch. The symbols * and ** showed significant differences (* *p* < 0.05, ** *p* < 0.01) between mutant Δ*PoxMKK1* and the control strain Δ*PoxKu70*, and between mutant Δ*PoxMKK1* and complementary strain C*PoxMKK1*, as assessed by Student’s *t*-test.

In order to examine the effects of *PoxMKK1* deletion on the vegetative growth and sporulation of *P. oxalicum*, the colony diameter and hypha biomass, as well as the spore number, were measured on solid or liquid medium, respectively. Fresh spores (0.5 × 10^6^) of the mutant ∆*PoxMKK1*, control strain ∆*PoxKu70*, and complementary strain C*PoxMKK1* were directly pointed on PDA and MMM plates containing distinct carbon sources cultivated for 4 days at 28 °C. Compared with the control strain Δ*PoxKu70* and complementary strain C*PoxMKK1*, the colony diameter of mutant Δ*PoxMKK1* became larger on PDA (*p* < 0.01, Student’s *t*-test), but not on glucose, SCS, or Avicel (Figure 2A,B). Furthermore, the colony color of Δ*PoxKu70* and C*PoxMKK1* on PDA plates was black-brown, while that of the Δ*PoxMKK1* mutant was black-green (Figure 2A). Notably, the colony center color of Δ*PoxKu70* and C*PoxMKK1* was pale green on MMM containing Avicel, while that of the Δ*PoxMKK1* mutant was grey, which might have resulted from different numbers of spores. Therefore, the spore number was quantified, and the result showed that the conidial production of Δ*PoxMKK1* was significantly less than that of the control and complementary strains on PDA, glucose, SCS, and Avicel cultivated for 5, 14, 14, and 7 days, respectively (Figure 2C).

**Figure 3:**
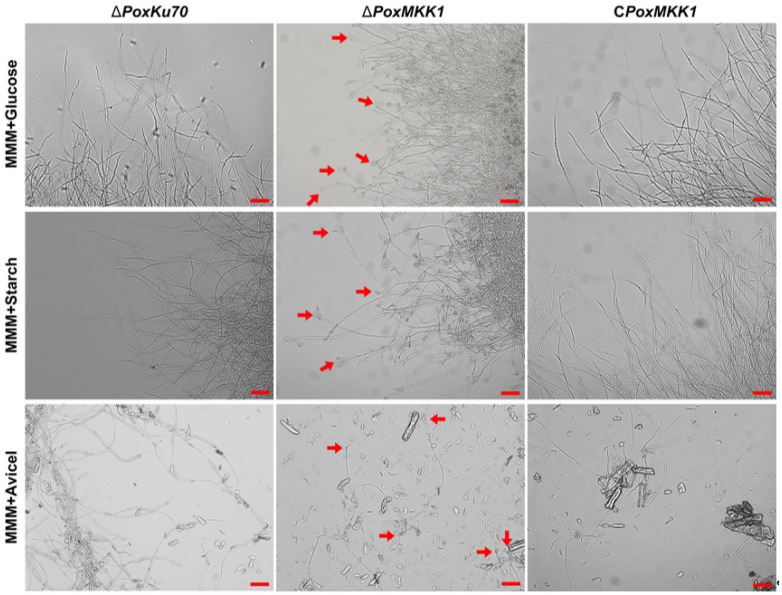
Microscopic images of hyphae in MMM containing 1.0% glucose, 1.0% SCS, and 2.0% Avicel as the solo carbon source at 28 °C for 24 h with 180 rpm (10^8^ conidia/mL). The red arrowheads point to conidiophores. Scale bars = 50 μm.

For samples grown under liquid culture conditions, the mycelium dry weight or intracellular protein were measured in MMM supplied with glucose, SCS, or Avicel, and CM. Surprisingly, the mycelial biomass of mutant Δ*PoxMKK1* had no significant difference relative to that of Δ*PoxKu70* when cultivated in MMM containing SCS or CM (Supplementary Figure S5A,B), whereas the growth of Δ*PoxMKK1* mutant decreased during the whole cultivation stage in MMM with either glucose or Avicel (Supplementary Figure S5C,D). In addition, the asexual spore yields of Δ*PoxMKK1* increased notably compared to those of Δ*PoxKu70* and C*PoxMKK1* in the aforementioned liquid media when grown for 6 days at 28 °C with shaking at 180 rpm (Supplementary Figure S6). Moreover, microscopic observation showed that there were a number of phialides for conidiation at the top segments of the hyphae in the ∆*PoxMKK1* mutant when cultivated on glucose, Avicel, or SCS for two days, whereas these were absent in both ∆*PoxKu70* and C*PoxMKK1* (Figure 3), suggesting that PoxMKK1 repressed mycelial development. Collectively, these findings indicated that PoxMKK1 was involved in vegetative growth and conidiation which was dependent on culture formats and media.

### 3.4. Loss of PoxMKK1 Alters PPDE Production of P. oxalicum under SmF and SSF

**Figure 4:**
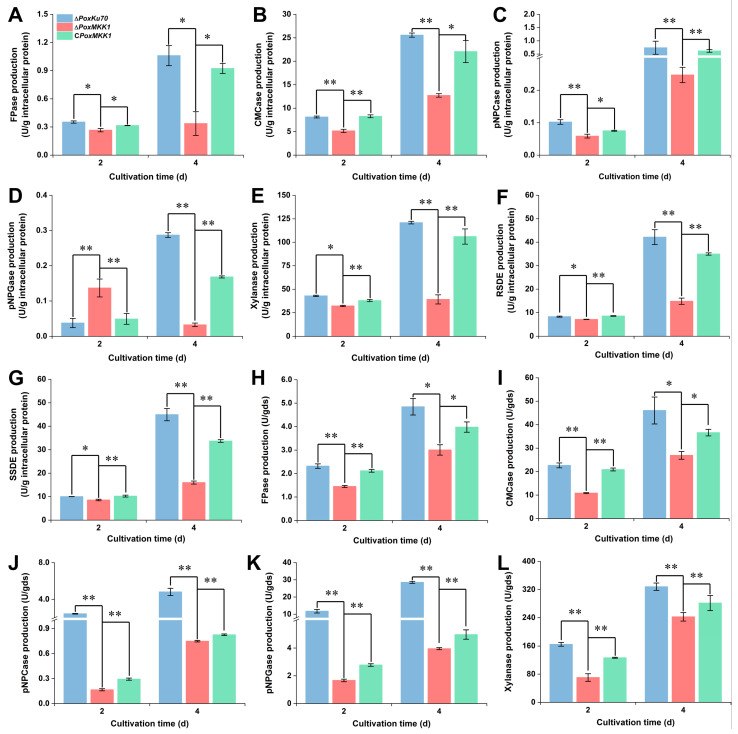
Protein kinase PoxMKK1 affects PPDE production in *P. oxalicum*. Cellulase (**A**−**D**,**H**−**K**) and xylanase (**E**,**L**) production are determined in liquid medium with 2.0% Avicel and solid medium containing wheat bran plus rice straw under SmF and SSF, respectively. (**F**) RSDE and (**G**) RSDE. Production was performed in liquid medium with 1.0% SCS. Enzymatic activity was assayed at 2–4 days after a shift from glucose. SSDE, soluble starch-degrading enzyme. RSDE, raw cassava starch-degrading enzyme. SmF, submerged fermentation. SSF, solid-state fermentation. Error bars indicate standard deviations of these results from three biological replicates. Significant differences are indicated by an asterisk between the deletion mutant Δ*PoxMKK1* and the control strain or the complementation strain C*PoxMKK1*, respectively (* *p* < 0.05, ** *p* < 0.01, Student’s *t*-test).

In order to further confirm the effects of *PoxMKK1* deletion on PPDE production in *P. oxalicum*, the mutant Δ*PoxMKK1* and control strain Δ*PoxKu70* were individually cultivated for 2–4 days after a shift from glucose, and their secreted PPDE productions were monitored. As depicted in Figure 4, when grown on Avicel under SmF for 2–4 days, the Δ*PoxMKK1* displayed decreased FPase, CMCase, pNPCase, and xylanase production by 24.6–68.3%, as compared with that in Δ*PoxKu70* (*p* < 0.05, Student’s *t*-test). Notably, Δ*PoxMKK1* showed a 2.6-fold increase in pNPGase production on day 2 and an 88.6% decrease on day 4 (Figure 4D). When cultivated on SCS under SmF for 4 days, both the RSDE and SSDE of the Δ*PoxMKK1* were reduced by 64.4% and 64.8%, respectively (Figure 4F,G). Interestingly, the yields of cellulase and xylanase by the Δ*PoxMKK1* were also depressed on WR under SSF by 37.4–93.2% and 26.0–57.2%, respectively (Figure 4H–L). As expected, the production of cellulase, xylanase, and amylase by the complementation strain C*PoxMKK1* was restored to the level of that by the Δ*PoxKu70* cultivated as described above (*p* < 0.05, Figure 4).

### 3.5. RNA-seq Analyses Revealed the Global Regulation of PoxMKK1 in P. oxalicum

In order to elucidate the functions of PoxMKK1 on genome-scale gene expression in *P. oxalicum*, the mutant Δ*PoxMKK1* and control strain Δ*PoxKu70* were first grown in glucose medium for 24 h, and then the mycelia were transferred to Avicel medium for 24 h. The samples were used to isolate total RNA and for sequencing. As displayed in Supplementary Figure S7, a good Pearson’s correlation coefficient was obtained (*R* > 0.92) among the three biological repeats for each strain. The generated data displayed that more than 98% of the clean reads of Δ*PoxKu70* and Δ*PoxMKK1* were successfully aligned to the *P. oxalicum* HP7-1 genome [20]. With the criteria of probability ≥0.8 and |Log2(Δ*PoxMKK1*_FRKM/Δ*PoxKu70*_FRKM)| ≥ 1.0, a total of 1114 differential expression genes (DEGs) were selected in mutant Δ*PoxMKK1*, relative to the control strain Δ*PoxKu70*, including 634 upregulated and 480 downregulated genes (Supplementary Table S2).

**Figure 5:**
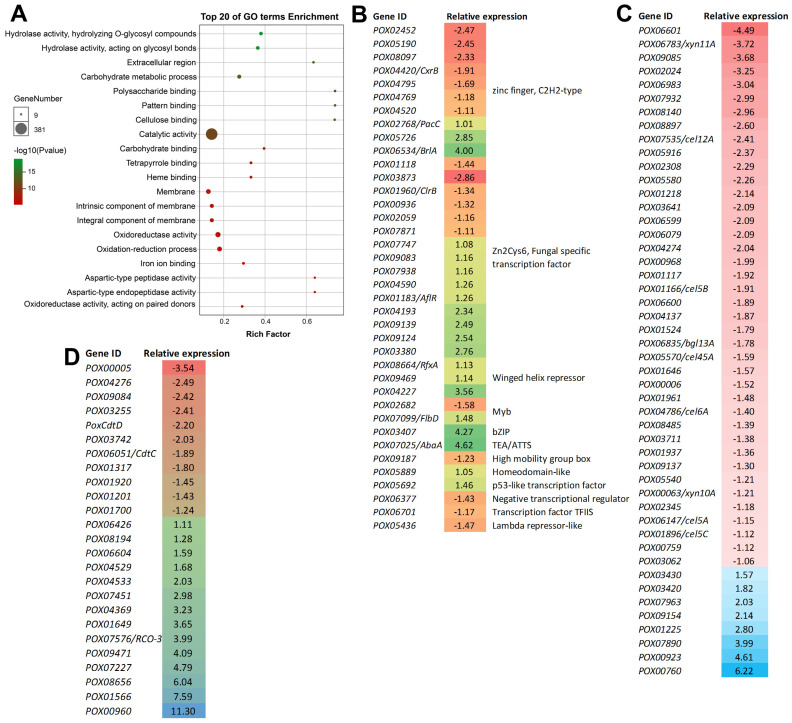
Transcriptomic analysis of the *P. oxalicum* mutant Δ*PoxMKK1* and the control strain Δ*PoxKu70* grown in MMM with 2.0% Avicel. Total RNAs for RNA sequencing were prepared from fungal hypha cultivated for 24 h after a shift. (**A**) The gene ontology (GO) annotation of differentially expressed genes (DEGs) for the top 20 GO terms’ enrichment. (**B**−**D**) Heatmap showing the transcription abundance of DEGs encoding putative CAZymes, transcription factors (TFs), and sugar transporters. FPKM, fragments per kilobase of exon per million mapped reads.

GO enrichment analyses implied that 15 of the top 20 enriched terms were associated with molecular function, such as hydrolase activity (GO: 0016798 and GO: 0004553), catalytic activity (GO: 0003824), and carbohydrate binding (GO: 0030246, GO: 0030247, and GO: 0030247). Additionally, the DEGs in the Δ*PoxMKK1*, which participated in the carbohydrate metabolic process and encoded extracellular region components, were also significantly enriched (Figure 5A).

Remarkably, of the 1114 DEGs, there were 133 genes encoding carbohydrate-active enzymes (CAZymes), including 48 plant cell-wall degrading enzymes (CWDEs). Of them, 40 DEGs were downregulated by 52.19–95.53% in mutant Δ*PoxMKK1*, such as one CBH gene (*cbh2*), seven EG genes (*Cel5B*, *Cel45A*, *Cel5A*, *eg2*, *Cel5C*, *POX04137*, and *POX06983*), five BGL genes (*Bgl1*, *POX00923*, *POX00968*, *POX03062*, and *POX03641*), five xylanase genes (*Xyn10A*, *Xyn11A*, *POX04274*, *POX06601*, and *POX05916*), two lytic polysaccharide monooxygenases genes (*AA9A* and *POX02308*), and two expansin-like protein genes (*POX01524* and *POX08485*) (Figure 5B).

Besides CAZyme-encoding genes, the DEGs also contained 38 TF-encoding genes (Figure 5C). Among them, 18 DEGs were downregulated (−2.86 < log2 (fold change) < −1.11) and 20 DEGs were upregulated (1 < log2 (fold change) < 4.7). Several known regulatory genes of PPDE production, such as *PoxCxrB* [26], *PoxClrB* [32], *PoxRfxA* [33], *PoxPacC* [34], *POX01118* [35], *POX05276* [26], *POX09124*, and *POX09469* [36] were found, as well as *PoxBrlA* [37], *PoxFlbD*, and *PoxAbaA—*known to activate conidiation in filamentous fungi [38]. Moreover, 25 DEGs encoding sugar transporters were found by InterPro screening in Δ*PoxMKK1*, such as *PoxCdtD* (log2 (fold change) = −2.20), *PoxCdtC* (log2 (fold change) = −1.89), and *PoxRCO-3* (log2 (fold change) = −3.99) (Figure 5D).

### 3.6. Regulation Kinetics of PoxMKK1 on the Expression of Major Genes Encoding PPDE and TFs in P. oxalicum under SmF and SSF

**Figure 6:**
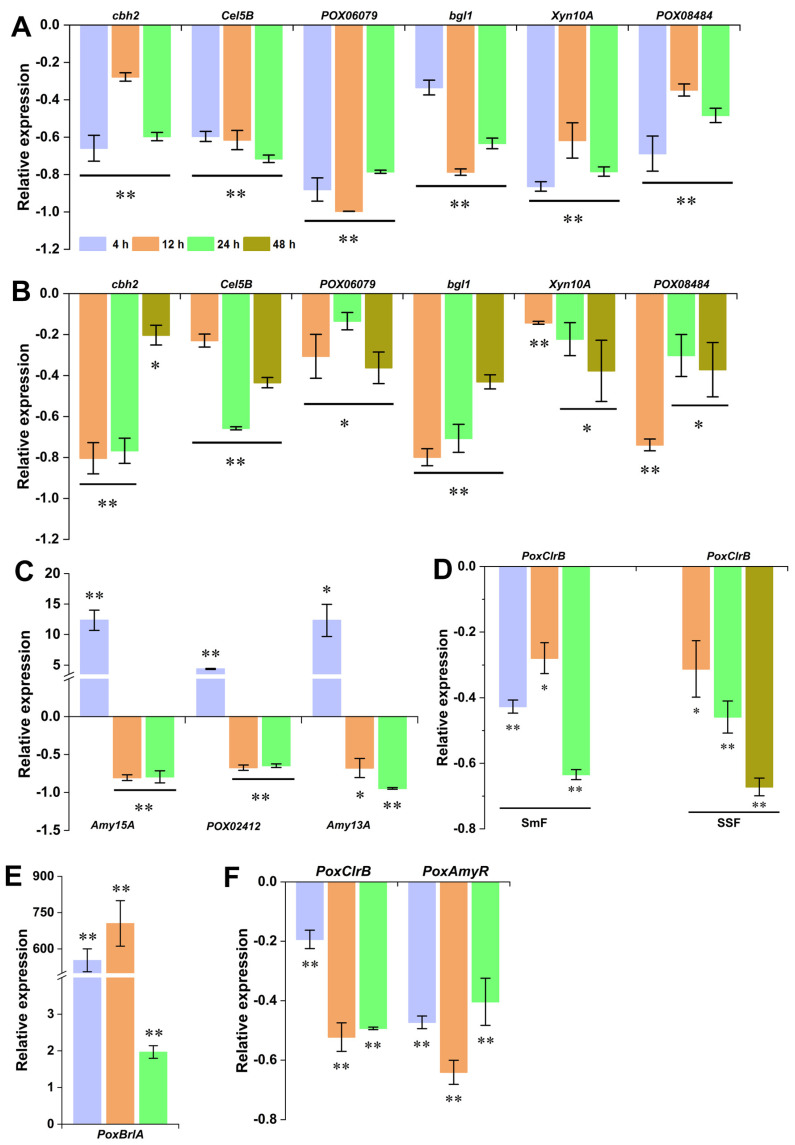
Key gene expression analysis of *P. oxalicum* strains by RT-qPCR assay. (**A**,**B**) Expression abundance of major cellulase and xylanase genes under SmF and SSF. (**C**) Expression abundance of major amylase genes. (**D**) Expression abundance of known transcription factor (TF) genes for regulating cellulase and xylanase production. (**E**) Expression abundance of known TF genes for regulating amylase production. (**F**) Expression abundance of *BrlA* gene involved in asexual development. The transcript levels of each gene were tested at three different times (at 4, 12, and 24 h under SmF and at 12, 24, and 48 h under SSF) after a transfer and were then standardized against those of the control strain Δ*PoxKu70*. * *p* < 0.05 and ** *p* < 0.01 according to Student’s *t*-test indicated significant differences between the mutant Δ*PoxMKK1* and the control strain.

In order to further validate the influences of PoxMKK1 on major PPDE- and TF-encoding genes at the transcriptional level, RT-qPCR analysis was conducted on selected genes as shown in Figure 6. Deletion of *PoxMKK1* significantly reduced the expression of four genes (*cbh2*, *Cel5B*, *Bgl3A*, and *POX06079*) encoding major cellulase and two xylanase genes (*Xyn10A* and *Xyn11B*) in *P. oxalicum* under SmF and SSF by 9.12–99.68%, as compared with the control Δ*PoxKu70* strain (Figure 6A). Notably, the transcriptional levels of amylase genes (*Amy15A*, *POX02412*, and *Amy13A*) in Δ*PoxMKK1* were significantly higher than those in Δ*PoxKu70* by 4.38- to 12.34-fold under 4 h of induction by SCS with SmF, whereas these decreased by 64.66–94.61% at later induction stages (Figure 6C).

Furthermore, under SmF with Avicel and SSF with WR, the transcriptional level of cellulase activator gene *PoxClrB* decreased by 27.95–63.45% and 31.23–67.22% in Δ*PoxMKK1* compared with those in Δ*PoxKu70*, respectively (Figure 6D). In contrast, the expression of *PoxBrlA* was increased 1.97–705.31 times in Δ*PoxMKK1*, which is consistent with the RNA-seq data (Figure 6E). Under SmF with SCS, *PoxClrB* and *PoxAmyR* expression was markedly less in Δ*PoxMKK1* than in Δ*PoxKu70* (Figure 6F).

### 3.7. Comparative Analysis of PoxMK1 and PoxMKK1 Regulons in P. oxalicum

As previously described, PoxMKK1 was an orthologue of Ste7 in *S. cerevisiae*, which was located in upstream of the MAP kinase Fus3/Kss1 in the mating/filamentation–invasion signaling pathway. Therefore, it was necessary to ascertain the overlapped regulons mediated by PoxMKK1 and PoxMK1 in *P. oxalicum*.

**Figure 7:**
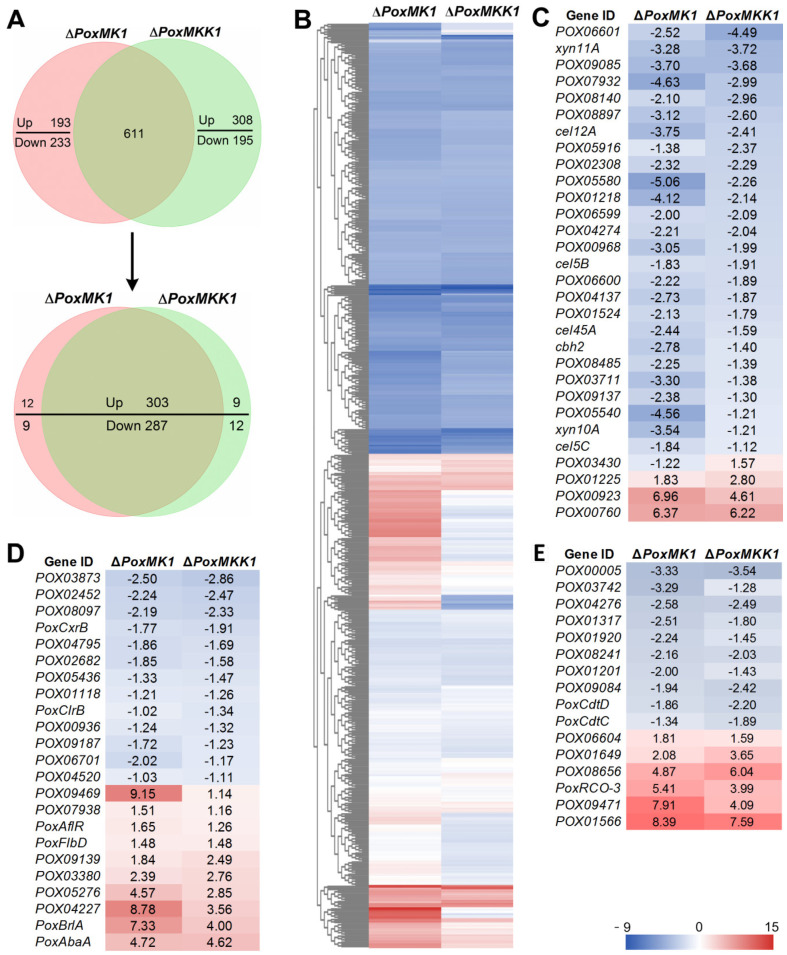
Regulon comparison of *PoxMK1* and *PoxMKK1* deduced by Avicel for 24 h. (**A**) Number of co-regulated differentially expressed genes (DEGs). (**B**) Heatmap illustrating co-regulated DEGs; (**C**−**E**) DEGs encoding predicted CAZymes, TFs, and sugar transporters.

Comparative analysis suggested that there were 611 shared DEGs in the regulons of *PoxMKK1* and *PoxMK1*. Among them, the regulatory function of 590 co-regulated genes was consistent between Δ*PoxMKK1* and Δ*PoxMK1*, including 303 upregulated and 267 downregulated genes, compared with the control strain Δ*PoxKu70* (Figure 7A,B). Interestingly, 7 cellulase genes (i.e., 1 *cbh*, 5 *eg*s, and 1 *bgl*), 5 xylanase genes, 23 TF genes (e.g., *PoxCxrB*, *PoxClrB*, *PoxBrlA*, *PoxFlbD*, and *PoxAbaA*), and 16 putative sugar transporter genes (e.g., *PoxCdtD PoxCdtC*, and *PoxRCO-3*) were found in the co-regulated DEGs set (Figure 7C–E), indicating that the Δ*PoxMK1* and Δ*PoxMKK1* mutants shared similar transcriptional profiles.

## 4. Discussion

As one of the multicellular eukaryotic organisms, the growth and development of filamentous fungi are regulated by evolutionarily conserved signal transduction pathways in which protein kinases are major players. In this study, the function of protein kinase PoxMKK1, a mediate component of three-tiered cascade kinases in the Fus3/Kss1-MAP kinase module, was investigated in *P. oxalicum* for the first time. PoxMKK1 is involved in modulating the production of PPDE—including cellulase, xylanase, and amylase, under both SSF and SmF conditions—the regulation of vegetative growth and conidiation.

In previous studies, MAPK modules were found to be involved in the regulation of the production of PPDE in fungi. For instance, three MAPKs are identified as Tmk1, Tmk2, and Tmk3 in *T. reesei*, which are homologous to yeast Hog1, Slt2, and Fus3, respectively [39]. Among of them, Tmk3 promotes cellulase production, whereas Tmk2 represses cellulase formation [40,41]. Deletion of gene *Tmk1* improves cellulase formation, but does not influence the expression of major cellulase genes [39]. However, when cultivated on sugarcane bagasse, the production of cellulase and xylanase by mutant Δ*tmk2* decreases significantly by repressing the transcription of major PPDE-encoding genes [42]. The upstream components of the Tmk3 signaling cascade, including TrSho1, TrSte20, and TrYpd1, differentially regulate cellulase production. Loss of *TrSte20* or repression of *TrSho1* significantly diminishes the transcriptional levels of cellulase genes, whereas overexpression of *TrYpd1* reduces the production of cellulase and repression of *TrYpd1* hardly affects cellulase induction [43]. In *A. nidulans*, xylanase activity is significantly reduced in the Δ*ste7* and Δ*mpkB* mutants, but it is significantly increased in the Δ*pbsA* mutant induced by xylose and/or glucose, especially after 72 h [44]. In some plant pathogenic fungi, such as *F. graminearum* [45], *A. brassicicola* [46,47], and *Valsa mali* [48] MAPK pathways also play an important role in the secretion of PPDE.

In the current study, the regulation of protein kinase PoxMKK1 for PPDE production was characterized in *P. oxalicum*. Although PoxMKK1 mainly positively regulated the production of cellulase and xylanase, fine regulation was different between SmF and SSF conditions. For example, under SmF, pNPGase production of mutant Δ*PoxMKK1* was significantly increased on day 2 after a transfer but was drastically decreased on day 4, while under SSF, pNPGase production remarkably reduced during the whole cultivation period compared with that of the control strain Δ*PoxKu70*. These conditions might have resulted from the fermentation format. During SSF, enough moisture was present on the surface of the porous and moist solid substrate particles to contribute to the development of fungal hyphae. Compared with SmF, SSF improves the kinetic parameters associated with growth and fungal morphology, modifies the expression of many genes, prevents catabolite repression, and increases the secretome complexity, which mimics their natural habitat [49,50]. Previous work demonstrated different transcription profiles of *P. oxalicum* cultivated under SSF and SmF. For instance, major cellulase genes increased their transcripts under SSF in comparison with those under SmF, but genes participating in the citric acid cycle were down-regulated, hinting that a distinct regulatory network was exhibited in *P. oxalicum* under SSF and SmF [36]. However, uncovering the detailed mechanism of PoxMKK1 functions would require further study under SSF and SmF, respectively.

Additionally, the restorative effect of cellulase and xylanase production was observed to result from gene *PoxMKK1* deletion under SSF, which also occurred in *T. reesei* Δ*tmk3* [41]. Accordingly, the effects of PoxMKK1 on PPDE production were dependent on carbon source, cultivation time and fermentation mode in *P. oxalicum*.

Moreover, it should be noted that the expression of major amylase genes including *amy15A*, *POX02412*, and *amy13A* was dynamically regulated by PoxMKK1; for instance, the expression of these genes was increased at 4 h of induction in the mutant Δ*PoxMKK1* in comparison with the Δ*PoxKu70*, whereas it was downregulated at 12 and 24 h. This result should be attributed to the induction of an extracellular complex carbon source. Starch consists of multiple glucose units that are linked by α-1,4-glycosidic bonds and branched by α-1,6-glycosidic bonds, which could be hydrolyzed by amylase. Amylase consists of four related enzymes, α-amylase (EC 3.2.1.1), glucoamylase (EC 3.2.1.3), α-glucosidase (EC 3.2.1.20), and 1,4-α-glucanbranching enzyme (EC 2.4.1.18). α-amylase breaks α-1,4-glycosidic bonds into amylopectin, or amylose straight chains, to release straight-chain and branched oligosaccharides of various lengths. Glucoamylase can cleave both α-1,4- or α-1,6-glucosidic bonds at the non-reducing ends of starch chains, or dextrins, to release glucose [51]. Filamentous fungi secrete a large number of starch-hydrolytic enzymes, all of which are induced by starch, dextrin, or maltose to different extents but depending on the requirement of fungal cells.

**Figure 8:**
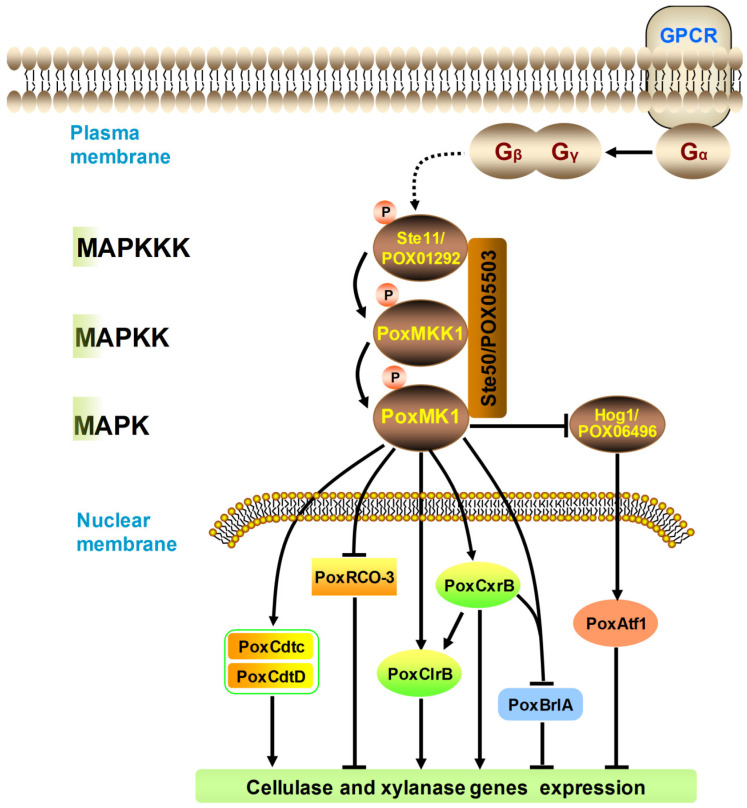
Schematic model for the regulatory network of protein kinase PoxMKK1 in *P. oxalicum* according to the data. GPCRs, G protein-coupled receptor. A P in orange-red circles represents phosphorylation events. The dashed lines represent that the pathway needs to be further confirmed. Arrows indicate activation, whereas bars indicate repression.

Furthermore, regulon comparative analysis showed that there was cross-talk regulation mediated by PoxMK1 and PoxMKK1 and that lots of DEGs, such as essential TF and sugar transporter genes, are co-regulated by them (Figure 8). ClrB is a crucial TF for cellulase activation in the presence of cellulose [52], while BrlA, as a central regulator of conidiation, not only plays an extensive role in the regulation of secondary metabolism, but also negatively regulates the expression of cellulase genes [37]. CxrB is a C2H2-type zinc finger TF for positively regulating the production of cellulase and xylanase, which directly bound the promoter regions of *BrlA* [22,26]. The sugar transporters are important for the utilization of lignocellulose, and of them, cellodextrin transporters CdtC and CdtD are necessary for the induction of cellulase expression [53]. A non-transporting glucose sensor, RCO-3, is involved in the regulation of the glucose transport system, and leads to carbon catabolite repression [54]. Additionally, direct interaction between PoxMK1 and POX06496/Hog1 was detected in Y2H assays. Deletion of gene *PoxMK1* enhances the phosphorylation of POX06496/Hog1, which phosphorylates PoxAtf1 to depress the expression of cellulase and xylanase genes [23]. These results reveal that PoxMKK1 may regulate the production of cellulase and xylanase via PoxMK1.

In *S. cerevisiae*, the heterotrimeric G-protein is initially separated into the Gα and Gβγ response to external signals. The Gβγ recruits the scaffold protein Ste5, and then the Ste5 assembles Ste11, Ste7, and Fus3/Kss1 [7]. The adaptor protein Ste50 also tethers Ste11, which contributes to the phosphorylation of Ste11. However, the orthologs of Ste5 do not exist in many filamentous fungi [10]. Therefore, it can be postulated that the Fus3/Kss1 signaling pathway regulates the cellulase and xylanase production in a tetrameric manner, but further research will need to be carried out in order to confirm this.

In addition, vegetative growth and sporulation are two fundamental processes in filamentous fungi. Previous studies showed that a lack of Fus3/Kss1-type MAPK cascade was correlated with a reduced hyphal growth rate and asexual sporulation in *Neurospora crassa* [55], *Aspergillus flavus* [56], *A. fumigatus* [57], *Aspergillus niger* [58], and *Botrytis cinerea* [59]. In *Cryphonectria parasitica*, although deletion of the gene *cpkk2* results in impaired growth on PDA plates, the biomass weight was similar to that of the wild-type strain in CM [60]. In *Colletotrichum higginsianum*, the *ChSte7* disruption mutant shows extremely decreased growth on PDA, biomass accumulation in PDB, and conidial germination, but produces as many conidia as the wild-type strain [61]. In *Fusarium graminearum*, the mutant Δ*FgSte7* grows obviously more slowly than the wild-type progenitor on the plates of PDA, CM, and minimum medium, and exhibits a significant decrease in conidiation after 4 days of incubation [45]. In *B. bassiana*, mutant Δ*mkk6* showed much less-severe growth defects on rich SDAY plates than on minimal CZA plates with different carbon or nitrogen sources, and the conidial yield was reduced by 78% on SDAY plates [14]. However, our study found that the mutant Δ*PoxMKK1* grew faster than the control strain on PDA plates, though it showed no significant difference on the plates of MMM with glucose, SCS, or Avicel. Moreover, the dry mycelium weight of Δ*PoxMKK1* in MMM with either glucose or Avicel showed a visible decrease compared to that of Δ*PoxKu70*, while no difference was displayed in CM and MMM with SCS. Interestingly, the asexual spores of the Δ*PoxMKK1* were reduced significantly on the above-mentioned solid plates, but they increased significantly in the liquid media. When cultivated for 2 days under SmF, phialides differentiated from the hypha tip of the Δ*PoxMKK1*. Furthermore, the transcriptions of conidiation-activator genes *FlbD*, *BrlA*, and *AbaA* in the Δ*PoxMKK1* increased by several to dozens of folds compared with those in the control strain under SmF, deduced by Avicel for 24 h after a shift. As is known, the TF BrlA is essential and sufficient for conidiation in filamentous fungi, and it is activated by FlbD and AbaA, and the AbaA is required for the differentiation of phialides [38], which suggests that spore production and hypha differentiation are consistent with the expression of conidiation-related TF genes in Δ*PoxMKK1* under SmF. Consequently, the Ste7 homologue PoxMKK1 affects vegetative growth and conidiation, which is not only related to species, but also to cultural conditions in filamentous fungi.

In future studies, we should investigate the influence of the simultaneous manipulation of gene *PoxMKK1* with other regulatory genes by, for example, constructing double/triple gene-deleted/overexpressed mutant, with the aim of maximizing PPDE production, which can potentially assist with the saccharification of lignocellulosic biomass. In addition, the heterologous expression of gene *PoxMKK1* in other systems such as *Trichoderma reesei* should be considered in order to possibly minimize the regulatory side-effects of its expression. Alternatively, the expression of gene *PoxMKK1* could be fine-tuned via the manipulation of promoter and terminator in *P. oxalium* in order to improve PPDE production.

In summary, our study reveals that the protein kinase PoxMKK1 modulated PPDE production, vegetative growth, and asexual sporogenesis in *P. oxalicum*. The functional characterization of PoxMKK1 will contribute to our understanding of the molecular mechanisms involved in morphogenesis and the regulation of processes of cellulolytic enzyme synthesis by the Fus3/Kss1 signaling pathway. In turn, this may allow for the development of strategies to construct an engineering strain with high PPDE production by rational design.

## Data Availability

All data are available in the article.

## Supplementary Materials

The following supporting information can be downloaded at: https://www.mdpi.com/article/10.3390/jof9040397/s1, Figure S1: Screening of PoxMK1-interacting protein in *P. oxalicum* using yeast two hybrid approach; Figure S2: Deletion mutants confirmation of PoxMK1-interacting protein gene in *P. oxalicum*; Figure S3: FPase production of the control strain Δ*PoxKu70* and mutants Δ*POX04853*, Δ*POX06496*, Δ*POX07588* and Δ*POX07948*; Figure S4: PCR verification of complementary strain C*PoxMKK1*; Figure S5: Biomass determination of *P. oxalicum* mutant Δ*PoxMKK1* and the control strain Δ*PoxKu70* in different liquid medium at 28 °C with 180 rpm for 72 h; Figure S6: Conidia number of *P. oxalicum* mutant Δ*PoxMKK1*, complementation strain C*PoxMKK1* and the control strain Δ*PoxKu70*; Figure S7: Pearson’s correlation analysis of the transcriptomes of *P. oxalicum* mutant strain Δ*PoxMKK1* and the control strain Δ*PoxKu70*; Table S1: Primers used in this study; Table S2: List of 1114 differentially expressed genes in *P. oxalicum* mutant ∆*PoxMKK1* compared with the control strain ∆*PoxKu70* in the presence of Avicel.
