# Supplementary material for: Protein Kinase PoxMKK1 Regulates Plant-Polysaccharide-Degrading Enzyme Biosynthesis, Mycelial Growth and Conidiation in Penicillium oxalicum
Source: J Fungi (Basel). 2023 Mar 23;9(4):397. doi: 10.3390/jof9040397 (PMC10143691; doi:10.3390/jof9040397)
Supplement: Supplementary file 1 [file jof-09-00397-s001.zip › jof-2250389-supplementary.pdf]

Journal of Fungi

MDPI

*Supplementary Material*

*Article*

# Protein Kinase PoxMKK1 Regulates Plant-Polysaccharide-Degrading Enzyme Biosynthesis, Mycelial Growth and Conidiation in *Penicillium oxalicum*

**Bo Ma, Xue-Mei Luo, Shuai Zhao * and Jia-Xun Feng ***

State Key Laboratory for Conservation and Utilization of Subtropical Agro-Bioresources, Guangxi Research Center for Microbial and Enzyme Engineering Technology, College of Life Science and Technology, Guangxi University, Nanning 530004, China

* Correspondence: shuaizhao0227@gxu.edu.cnb (S.Z.); jiaxunfeng@sohu.com (J.-X.F.); Tel.: +86-771-3239401 (S.Z.)

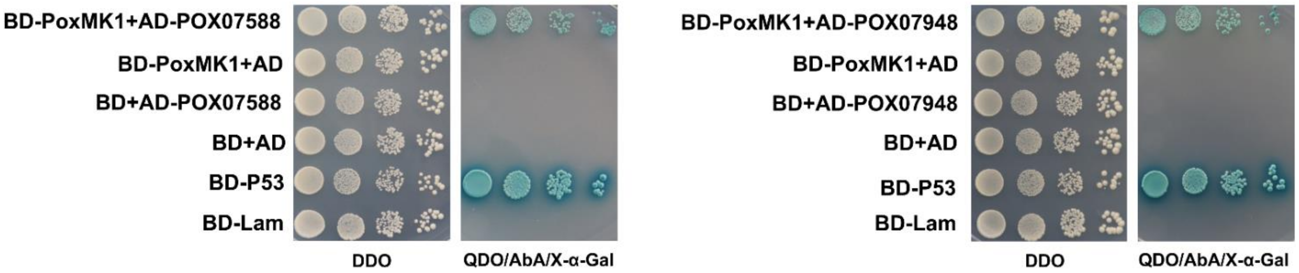


Figure S1. Screening of PoxMK1-interacting protein in *P. oxalicum* using yeast two hybrid approach. Y2HGold cells were co-transformed with pGBKT7-PoxMK1 and each of candidate prey plasmids, and then pointed on DDO and QDO/X/A plates at 30°C for 3–5 days, respectively. The presence of blue colonies indicated positive interaction. BD-53 was positive control, and BD-Lam was used as a negative control. BD, pGBKT7. AD, pGADT7.

Journal of Fungi

MDPI

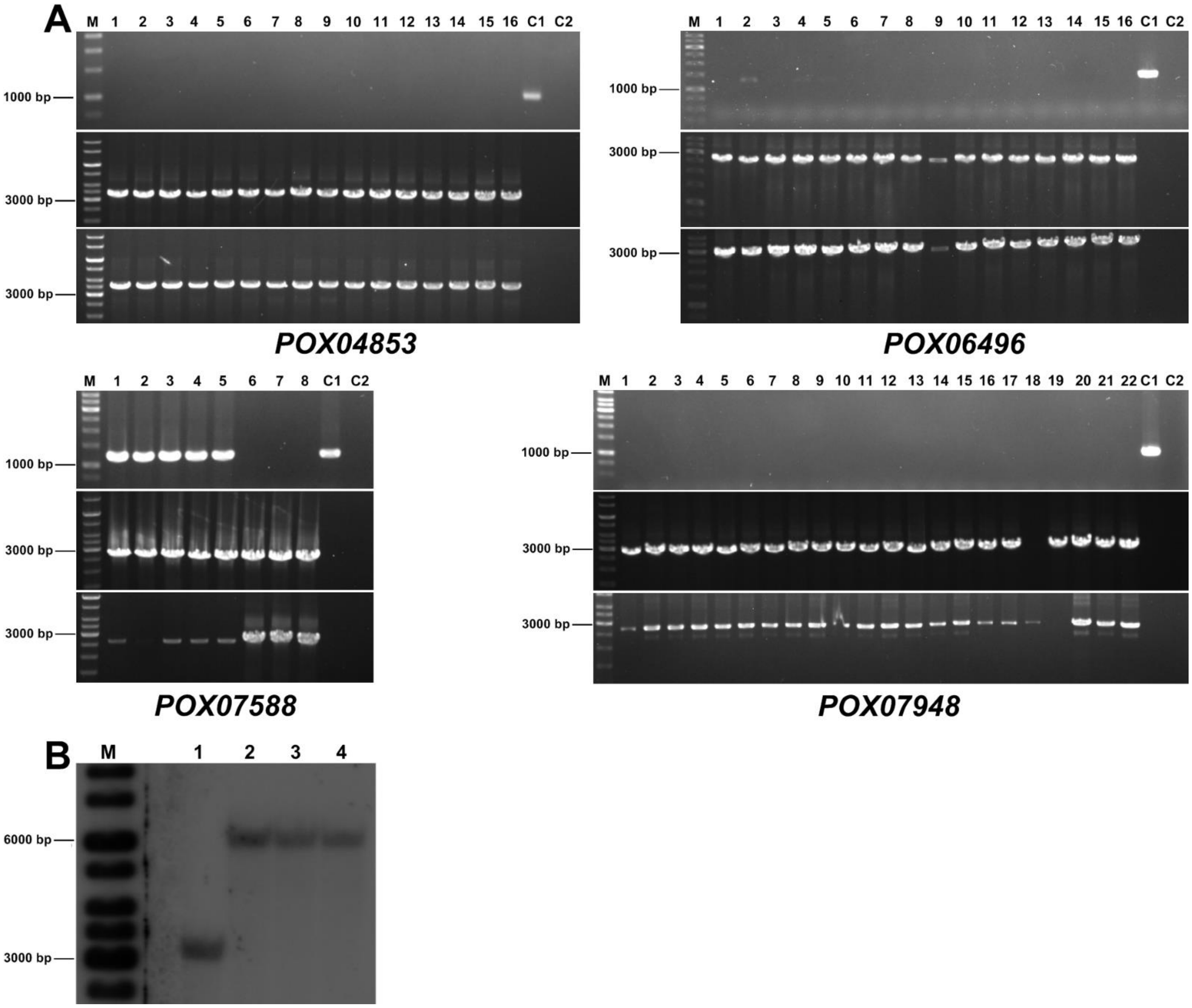


Figure S2. Deletion mutants confirmation of PoxMK1-interacting protein gene in *P. oxalicum*. (**A**) PCR verification. M, 10 kb DNA markers; Lane C1, the control strain Δ*PoxKu70*; Lane C2, dd$H_2O$; Lanes the rest, transformants for each candidate gene deletion mutant strain. The top panel shows amplification of target gene using primers Target gene V-F/Target gene V-R, the middle panel shows amplification of left-cross DNA region using primers Target gene L-F/G418 V-F, the bottom panel shows amplification of right-cross DNA region using primers G418 V-R/Target gene-R-R. (**B**) Southern blot analysis. M, 1.0 kb DNA marker; Lane 1, the control strain *PoxKu70*; Lane 2-4, three transformants for mutant Δ*POX07948*.

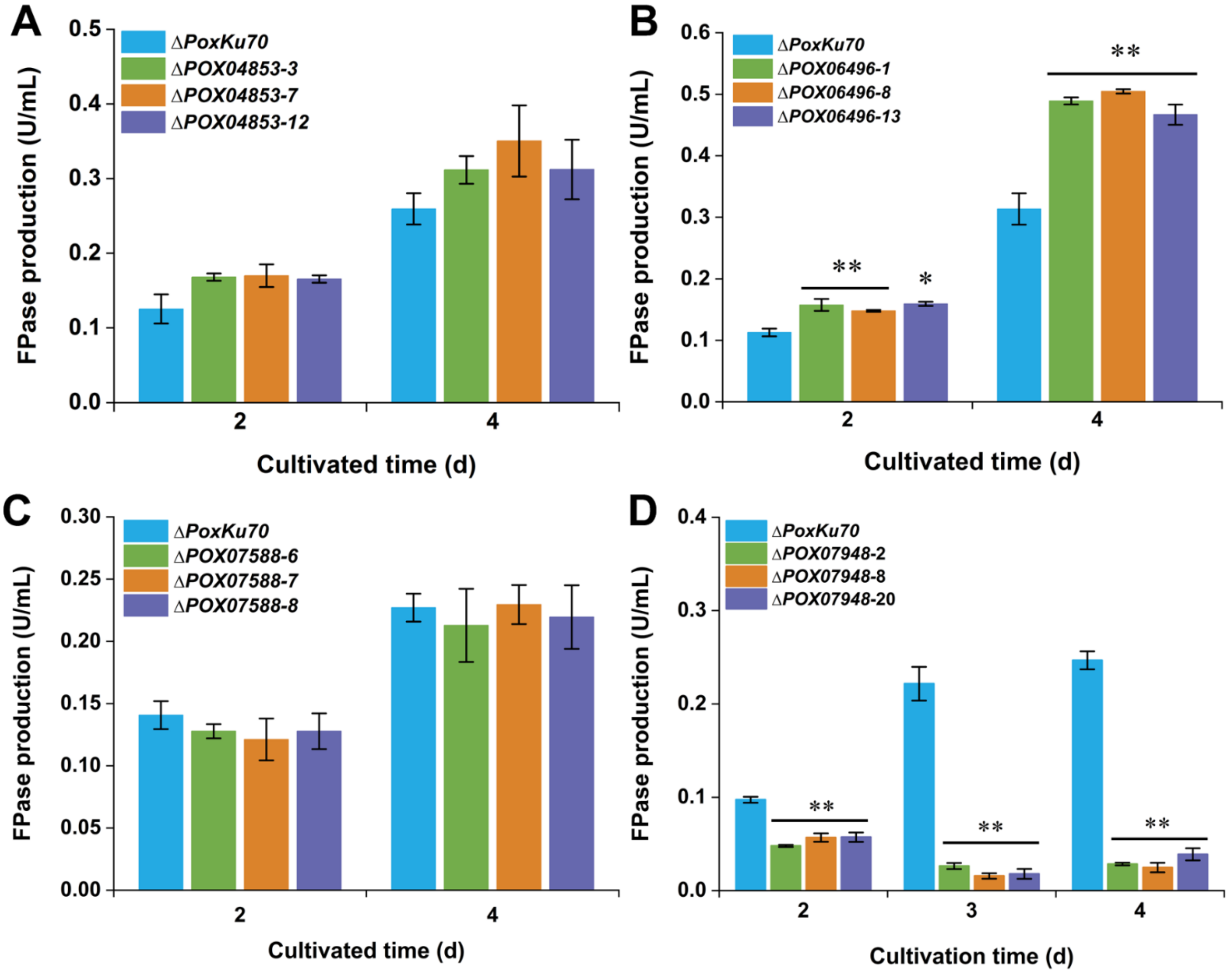


Figure S3. FPase production of the control strain Δ*PoxKu70* and mutants Δ*POX04588*, Δ*POX06496*, Δ*POX07588* and Δ*POX*07948. Enzyme production was measured in MMM using Avicel as the sole carbon source at 28°C for 2–4 days, after a transfer from glucose. MMM, minimum modified medium. The asterisk represents significant differences compared with the control strain (* $P < 0.05$, ** $P < 0.01$, Student's t test).

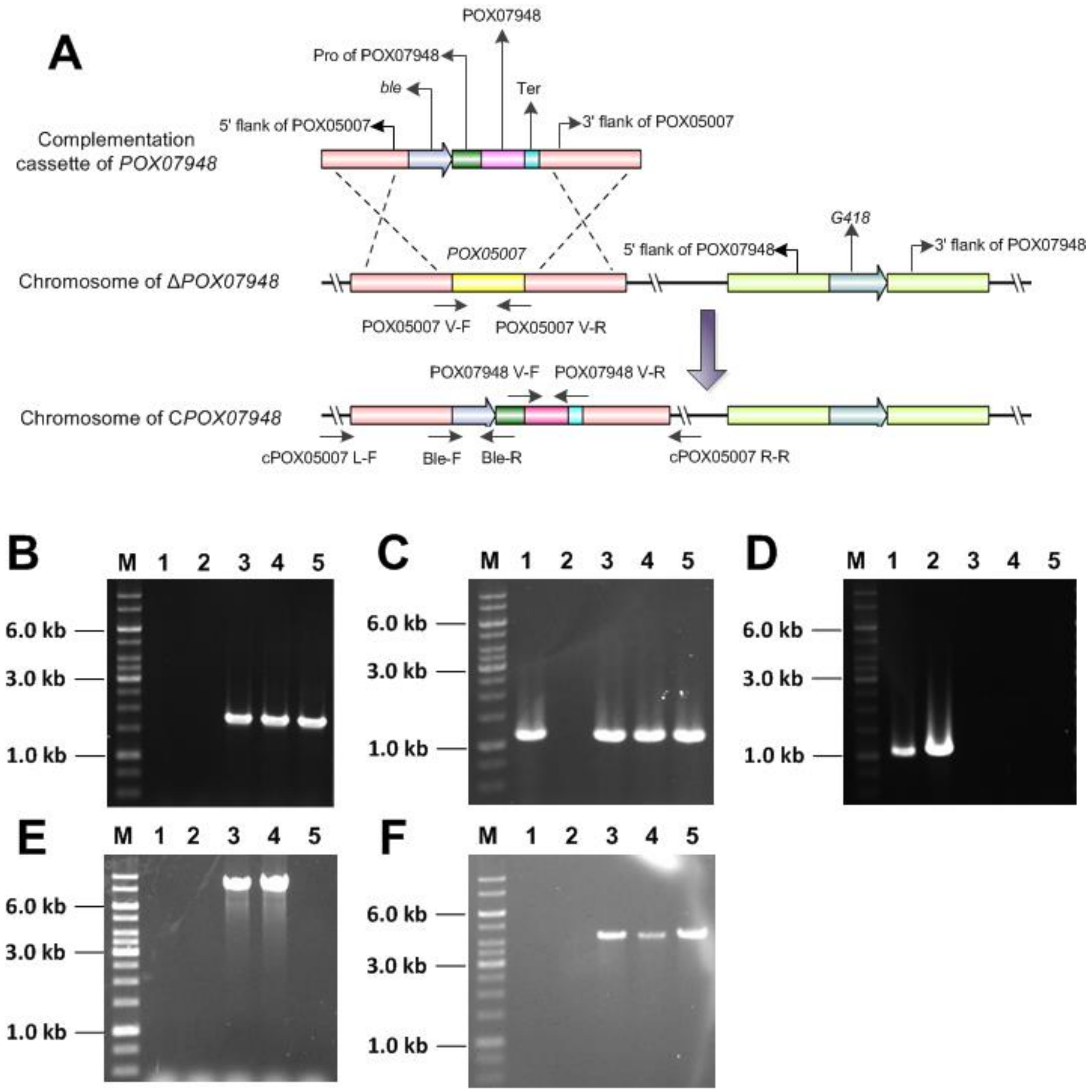


Figure S4. PCR verification of complementary strain C*PoxMKK1*. (A) Schematic diagram showing construction strategy of complementation strain. (**B**−**F**) PCR analysis. M: 1.0 kb maker; Lanes 1: Δ*PoxKu70*. Lanes 2: Δ*PoxMKK1*. Lane 3-5: three transformants for C*PoxMKK1*. (B) PCR amplification was performed with specific primers Ble-F/Ble-R. (**C**) PCR amplification was performed with primers POX07948-V-F/POX07948-V-F-R. (**D**) PCR amplification was performed with primers POX05007V-F/POX05007V-R. (**E**) PCR amplification was performed with primers cPOX05007-L-F/Ble-R. (**F**) PCR amplification was performed with primers Ble-F/cPOX05007-R-R.

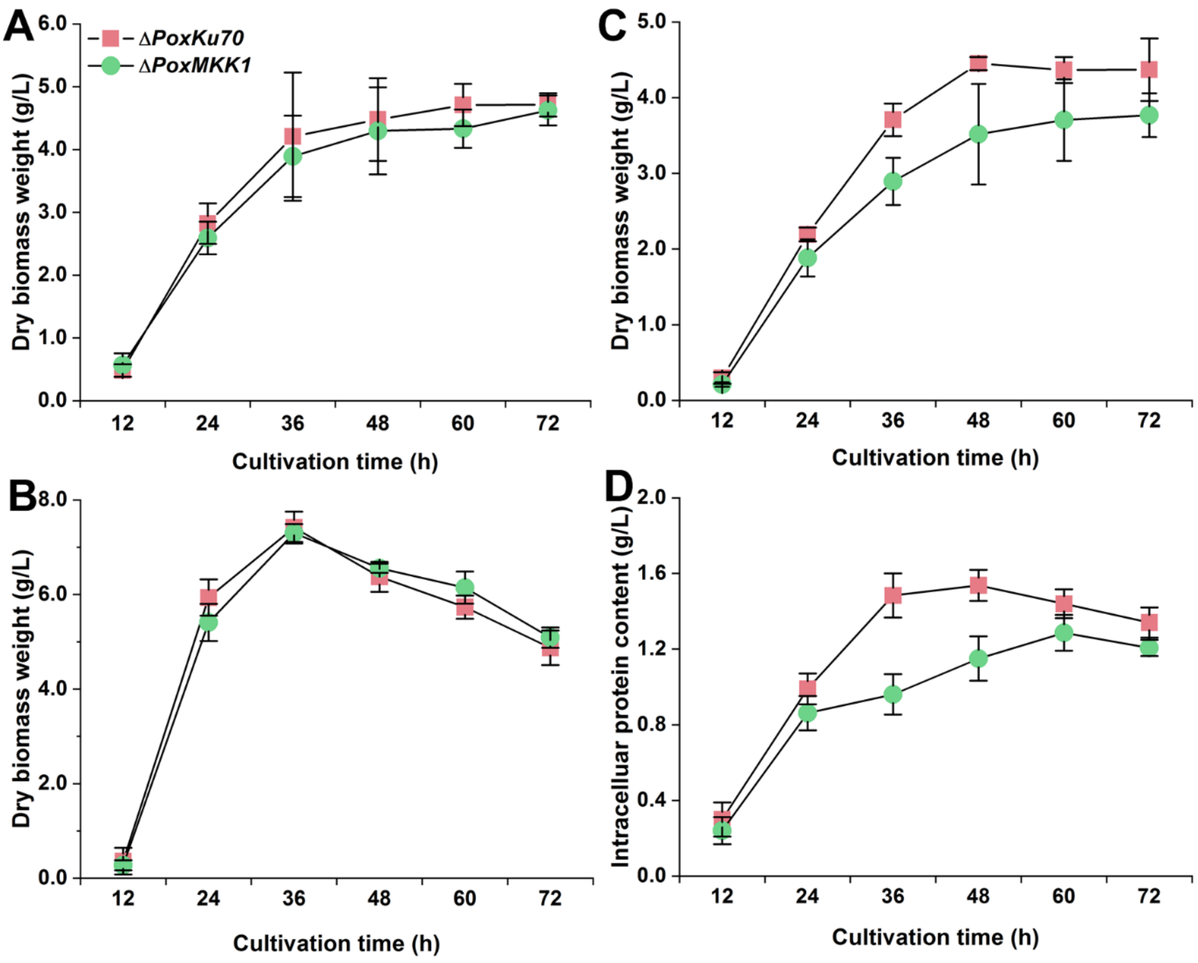


Figure S5. Biomass determination of *P. oxalicum* mutant Δ*PoxMKK1* and the control strain Δ*PoxKu70* in different liquid medium at 28°C with 180 rpm for 72 h. (**A**–**C**) MMM with 1.0% glucose, 1.0% SCS, and 2.0% Avicel. (**D**) complete medium. SCS, soluble corn starch.

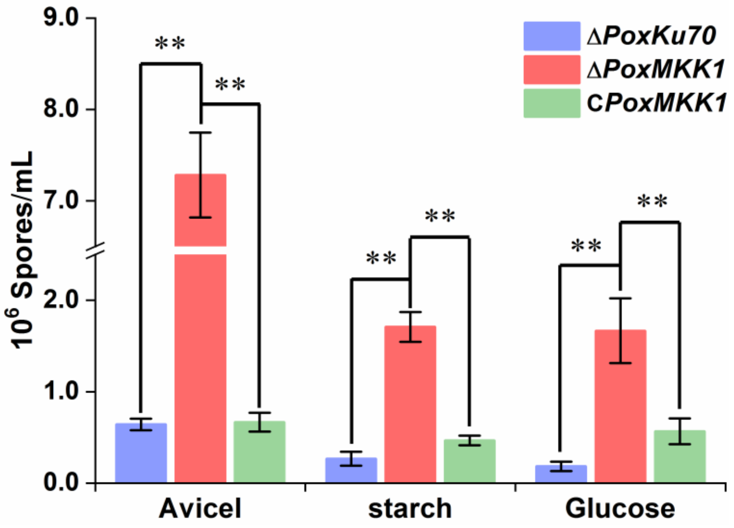


Figure S6. Conidia number of *P. oxalicum* mutant Δ*PoxMKK1*, complementation strain C*PoxMKK1* and the control strain Δ*PoxKu70* in MMM with 1.0% glucose, 2.0% Avicel, and 1.0% SCS and complete medium cultured for 6 days. The asterisk ** indicated significant differences ($P < 0.01$, Student's *t* test) between mutant Δ*PoxMKK1* and the control strain Δ*PoxKu70* or complementary strain C*PoxMKK1*.

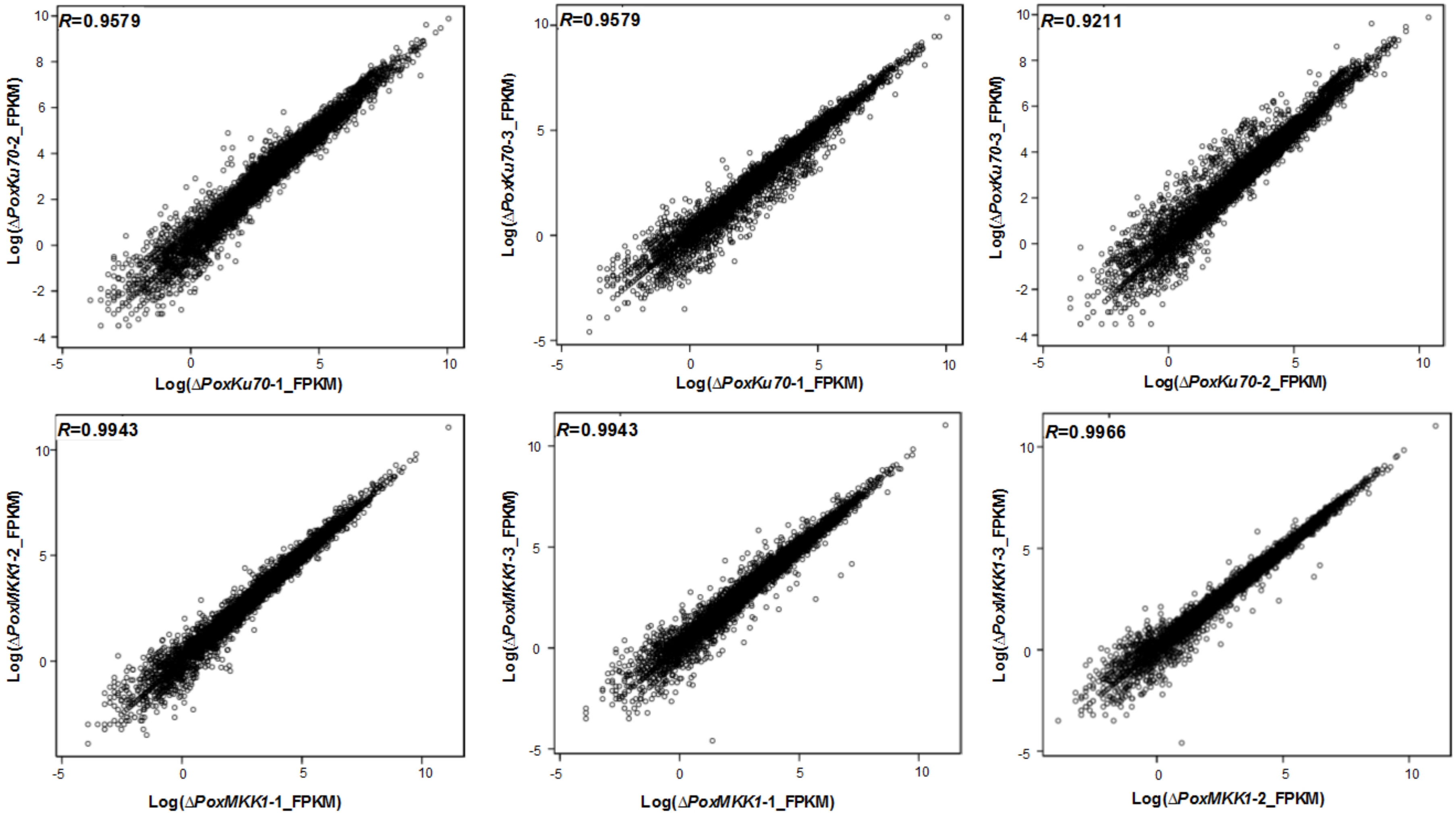


Figure S7. Pearson's correlation analysis of the transcriptomes of *P. oxalicum* mutant strain Δ*PoxMKK1* and the control strain Δ*PoxKu70*. FPKM, fragments per kilobase of exon per million mapped reads.

Journal of Fungi

**Table S1.** Primers used in this study.

| Primer name | Sequence (5' to 3') |
|---|---|
| **Primers for construction of deletion mutants** | |
| POX07948-L-F | GGCTATCTGGTGCCGTTC |
| POX07948-L-R | GGTAATCCTTCTTTCTAGA GGTGCTGATGAAGAGGTGGAC |
| POX07948-R-F | CAATATCATCTTCTGTCGAC GGGCTATCTGCCGAGATGT |
| POX079488-R-R | ATCACGACTGCTCTGTTTG |
| POX07948-V-F | TGCTGGCTGGTCTCTGCTAC |
| POX07948-V-R | TTGTGAGGTTTGTCCGCTTC |
| POX07948-N-F | TCCTCAACCGCATTCTCATC |
| POX07948-N-R | CTACTGCATCGACCTCCCAC |
| POX04853-L-F | CACGAAATGCTCGCCAGT |
| POX04853-L-R | GGTAATCCTTCTTTCTAGA GGAGCATTGACATTTGCTCT |
| POX04853-R-F | CAATATCATCTTCTGTCGAC GACGTTGATCTGTAAAAAGAAAAAAC |
| POX04853-R-R | AACAGACAAAATGGTGAAACTCG |
| POX04853-V-F | TGCTTGACCCATCCAAATCC |
| POX04853-V-R | TCCTCTGTGACGCCTGCC |
| POX04853-N-F | GCACCCTACACTTCCCCTC |
| POX04853-N-R | GCATCTCCTTTTAGAACCCA |
| POX06496-L-F | GTAGGTTAGGAATGGGGTGTTGC |
| POX06496-L-R | GGTAATCCTTCTTTCTAGA ACGCTGTTGAGCGGCGATA |
| POX06496-R-F | CAATATCATCTTCTGTCGAC GATAGCCGAGAAAGTGCTGAAAG |
| POX06496-R-R | TATGAAGAACCACTAACCACGCC |
| POX06496-V-F | TTCGGCTTGGTTTGGTAGGTCAT |
| POX06496-V-R | TGGTAGGGTGCGAGATACGGGTG |
| POX06496-N-F | TTGGTCTTTTGAGTCATCGTTC |
| POX06496-N-R | ATGGGGTTTCTACCTTGTTCTG |
| POX07588-L-F | ACCCACCTTCCTTGACCTC |
| POX07588-L-R | GGTAATCCTTCTTTCTAGA CTTCTCGTAGTCCTGCGTAT |
| POX07588-R-F | CAATATCATCTTCTGTCGAC GATTCCGTTTAGACCGACTC |
| POX07588-R-R | CAAGAAAGGCAAATCCCAT |
| POX07588-V-F | TCTTGGACCTTCTCCCTGTG |
| POX07588-V-R | TCCTGTTTGACGTATTTGGC |
| POX07588-N-F | ACCCACCTTCCTTGACCT |
| POX07588-N-R | CTTCTCCGAATGAAACTTG |
| G418-F | TCTAGAAAGAAGGATTACC |
| G418-R | GTCGACAGAAGATGATATT |
| G418-V-F | GTGAATGCTCCGTAACACCCAAT |
| G418-V-R | CGCTACTGCTTACAAGTGGGCTGAT |
| **Primers for Southern hybridization** | |
| sPOX07948-F | GGAAGAGAGGACCAAGACGA |
| sPOX07948-R | CTGGGTGAGGTGAGAGAAGG |
| **Primers for construction and validation of complementary strain** | |

| | |
|---|---|
| cPOX05007-L-F | GGAACAGGTGACCCCTATGG |
| cPOX05007-L-R | TAGAGGTAATCCTTCTTTCTAGA GATGGACGAGACGGTACGA |
| cBle-F | TCGTACCGTCTCGTCCATC TCTAGAAAGAAGGATTACCTCTA |
| cBle-R | AGTTTATCCGCTGGATTCTCC CTGGATCTCAACAGCGGT |
| cPOX07948-F | GGAGAATCCAGCGGATAAACT |
| cPOX00158-R | CCCATCACACCACTAACAATA |
| cPOX05007-R-F | TATTGTTAGTGGTGTGATGGG ATGTAGATCACCAAGTTGCA |
| cPOX05007-R-R | CGTCAATCACTTCCTGCGAA |
| cPOX05007-N-F | GAACAGGTGACCCCTATGGAAG |
| cPOX05007-N-R | GATATCGCCACTAACGTCG |
| POX05007V-F | GCAAGGTCCCCCAGAGTG |
| POX05007V-R | CCGAAGATGGAGAAGCCA |
| cPOX07948-F | GGAAGAGAGGACCAAGACGA |
| cPOX07948-R | CTGGGTGAGGTGAGAGAAGG |
| Ble-F | TCTAGAAAGAAGGATTACCTCTA |
| Ble-R | AGTTTATCCGCTGGATTCTCC |
| **Primers for RT-qPCR** | |
| qPOX04786-F/Cel6A | TACTACGCTTCCGAGGTTCAGAG |
| qPOX04786-R/Cel6A | GTGTCCAGCCAAACGAAGG |
| qPOX01166-F/Cel5B | CGATACTACGGCAACATCATCAC |
| qPOX01166-R/Cel5B | AGGCACCAGTCCACGAGTTT |
| qPOX06079-F | GGATAAGAACGGAGTCAGTGTCG |
| qPOX06079-R | GCATTGATGTAATCGGTGAAGTA |
| qPOX06835-F/bgl1 | GTGCTGGATGGGAACAGGA |
| qPOX06835-R/ bgl1 | TACGAACGCCGAGAGGAGA |
| qPOX00063-F/Xyn10A | TACCTCCCCATCGCCTTT |
| qPOX00063-R/Xyn10A | CTTGGCACCGTAGGACTGAAC |
| qPOX08484-F | ACAAGCACACGCAGGTCAA |
| qPOX08484-R | CGCTGAAGTGGTTGGCAGT |
| qPOX01960-F/ClrB | CTTCCAGGCGTCTCTCGTTC |
| qPOX01960-R/ClrB | CGCTTGCTGGCTTCGTAAA |
| qPOX00972-F/ClrC | CGGAGCGGCGACGGATT |
| qPOX00972-R/ClrC | TTTGGCGGGCGTGATGG |
| qPOX09352-F/amy13A | CTGACGGCTGCCCAATG |
| qPOX09352-R/amy13A | CCAAATCGCAGTAAATCCC |
| qPOX01356-F/amy15A | TTACTACACCTGGACCCGC |
| qPOX01356-R/amy15A | AGTCAATCAAGGCAATGGC |
| qPOX02412-F/amy15A | TATGTGGATTCCTTCCGCTCTA |
| qPOX02412-R/amy15A | ATGGATTGCCTCCTTGGT |
| qPOX03890-F/amyR | ACCCAGCCAGGGAACCAC |
| qPOX03890-R/amyR | CATTCCGATGCCGTGAGC |
| qPOX06534-F/BrlA | ATCCGAACTCATCGTCAACC |
| qPOX06534-R/BrlA | CCAGGCACCCAGCACACA |
| qPOX09428-F/actin | CTCCATCCAGGCCGTTCTG |
| qPOX09428-R/actin | CATGAGGTAGTCGGTCAAGTCAC |

| Primers for Y2H | |
|---|---|
| PoxMK1BD-F | CATGGAGGCCGAATTC ATGATGCGGCGATGGACAA |
| PoxMK1BD-R | GCAGGTCGACGGATCC TCACCGCATGATCTCCTCGTAG |
| Pox07948AD-F | GCCATGGAGGCCAGTGAA ATGGCAGATCAGTTCAAGG |
| Pox07948AD-R | CCCACCCGGGTGGAATT CTTACGACGAAGGCATCTG |
| AD-F | GGAGTACCCATACGACGTACC |
| AD-R | TATCTACGATTCATCTGCAGC |

**Table S2** List of 1114 differentially expressed genes in *P. oxalicum* mutant Δ*PoxMKK1* compared with the control strain Δ*PoxKu70* in the presence of Avicel.

| Name | Putative TFs | CWDEs | CAZyme family | IPR define | log2 (Δ*PoxMKK1*_FPKM/Δ*Poxku70*_FPKM) | Probability | Up/Down |
|---|---|---|---|---|---|---|---|
| *POX00001* | NA | NA | NA | NA | -1.99 | 0.91 | Down |
| *POX00005* | NA | NA | NA | IPR005828, IPR005829, IPR020846 | -3.54 | 0.96 | Down |
| *POX00006* | NA | β-xylosidase | GH43 | IPR006710, IPR013320, IPR023296 | -1.52 | 0.88 | Down |
| *POX00014* | NA | NA | NA | IPR004834, IPR004835, IPR013616, IPR029044 | 2.95 | 0.93 | Up |
| *POX00029* | NA | NA | NA | IPR002155, IPR016039, IPR020610, IPR020615, IPR020616, IPR020617 | 1.09 | 0.88 | Up |
| *POX00041* | NA | NA | NA | IPR002937, IPR008150, IPR014105, IPR023753 | 1.20 | 0.89 | Up |
| *POX00048* | NA | NA | NA | NA | -1.19 | 0.92 | Down |
| *POX00063* | NA | Endo-β-1'4-xylanase | CBM1; GH10 | IPR000254, IPR001000, IPR013781, IPR017853, IPR031158 | -1.21 | 0.93 | Down |
| *POX00073* | NA | NA | NA | NA | -3.19 | 0.96 | Down |
| *POX00076* | NA | NA | NA | NA | 2.54 | 0.93 | Up |
| *POX00077* | NA | NA | NA | IPR000873, IPR005914, IPR020845 | -1.19 | 0.84 | Down |
| *POX00078* | NA | NA | NA | IPR002129, IPR010107, IPR015421, IPR015424 | -1.93 | 0.95 | Down |
| *POX00081* | NA | NA | NA | NA | 2.99 | 0.92 | Up |
| *POX00089* | NA | NA | GH18 | IPR001223, IPR001579, IPR011583, IPR013781, IPR017853, IPR029070 | 2.62 | 0.97 | Up |
| *POX00105* | NA | NA | GH55 | IPR011050, IPR012334, IPR024535 | 3.75 | 0.97 | Up |
| *POX00111* | NA | NA | NA | NA | 1.99 | 0.93 | Up |
| *POX00112* | NA | NA | NA | NA | 2.39 | 0.89 | Up |
| *POX00120* | NA | NA | NA | IPR001958, IPR011701, IPR020846 | 1.75 | 0.95 | Up |
| *POX00142* | NA | NA | NA | NA | 2.21 | 0.89 | Up |
| *POX00144* | NA | NA | AA7 | IPR006094, IPR012951, IPR016166, IPR016169 | 1.71 | 0.89 | Up |
| *POX00154* | NA | NA | NA | NA | -1.70 | 0.88 | Down |
| *POX00155* | NA | NA | NA | IPR011701, IPR020846 | -1.28 | 0.84 | Down |

| | | | | | | | |
|---|---|---|---|---|---|---|---|
| *POX00158* | NA | NA | NA | IPR000719, IPR002290, IPR003527, IPR008271, IPR011009, IPR017441 | -1.07 | 0.91 | Down |
| *POX00169* | NA | NA | NA | IPR018825, IPR018827 | 1.45 | 0.94 | Up |
| *POX00170* | NA | NA | NA | IPR002925, IPR029058 | -1.51 | 0.94 | Down |
| *POX00196* | NA | NA | NA | NA | 1.72 | 0.86 | Up |
| *POX00202* | NA | NA | NA | IPR007250, IPR016360 | 3.04 | 0.94 | Up |
| *POX00209* | NA | NA | GH17 | IPR013781, IPR017853 | 6.23 | 0.98 | Up |
| *POX00224* | NA | NA | NA | IPR004164, IPR004165, IPR012791, IPR012792, IPR014388 | 1.14 | 0.84 | Up |
| *POX00244* | NA | NA | NA | NA | -6.59 | 0.80 | Down |
| *POX00246* | NA | NA | NA | NA | -1.54 | 0.92 | Down |
| *POX00251* | NA | NA | GH37 | IPR001661, IPR008928, IPR011120, IPR018232 | 1.15 | 0.87 | Up |
| *POX00260* | NA | NA | NA | IPR001461, IPR021109 | -2.38 | 0.95 | Down |
| *POX00270* | NA | NA | NA | NA | 1.18 | 0.92 | Up |
| *POX00271* | NA | NA | NA | IPR022024 | 1.08 | 0.91 | Up |
| *POX00288* | NA | NA | NA | NA | 3.27 | 0.94 | Up |
| *POX00319* | NA | NA | NA | NA | 3.20 | 0.82 | Up |
| *POX00322* | NA | NA | NA | NA | -1.99 | 0.90 | Down |
| *POX00341* | NA | NA | NA | IPR001464, IPR009117, IPR018502 | 1.68 | 0.92 | Up |
| *POX00347* | NA | NA | CBM21 | IPR005036 | -1.10 | 0.89 | Down |
| *POX00351* | NA | NA | NA | NA | 2.42 | 0.91 | Up |
| *POX00353* | NA | NA | NA | NA | 3.31 | 0.96 | Up |
| *POX00361* | NA | NA | NA | IPR002155, IPR016039, IPR020610, IPR020613, IPR020615, IPR020616, IPR020617 | 1.20 | 0.92 | Up |
| *POX00398* | NA | NA | NA | IPR001155, IPR013785 | 1.08 | 0.83 | Up |
| *POX00401* | NA | NA | NA | NA | 1.83 | 0.94 | Up |
| *POX00424* | NA | NA | NA | IPR004839, IPR015421, IPR015422, IPR015424 | -1.56 | 0.91 | Down |
| *POX00437* | NA | NA | NA | IPR013538, IPR015310, IPR023393 | 1.05 | 0.90 | Up |
| *POX00438* | NA | NA | NA | NA | -1.04 | 0.85 | Down |
| *POX00439* | NA | NA | NA | IPR008253 | 5.40 | 0.97 | Up |
| *POX00449* | NA | NA | NA | IPR001849, IPR011993 | -1.45 | 0.93 | Down |

| | | | | | | | |
|---|---|---|---|---|---|---|---|
| *POX00498* | NA | NA | NA | IPR000089, IPR001078, IPR004167, IPR011053, IPR015761, IPR023213 | 2.57 | 0.90 | Up |
| *POX00523* | NA | NA | NA | IPR004045, IPR005442, IPR010987, IPR012336 | -1.18 | 0.87 | Down |
| *POX00540* | NA | NA | NA | IPR027842 | -1.08 | 0.92 | Down |
| *POX00555* | NA | NA | NA | IPR004843, IPR008139, IPR011160, IPR029052 | -5.27 | 0.83 | Down |
| *POX00557* | NA | NA | NA | NA | 1.12 | 0.87 | Up |
| *POX00561* | NA | NA | NA | NA | -1.86 | 0.82 | Down |
| *POX00562* | NA | NA | NA | IPR000073, IPR002410, IPR005944, IPR029058 | -2.37 | 0.94 | Down |
| *POX00571* | NA | NA | NA | NA | -1.47 | 0.94 | Down |
| *POX00605* | NA | NA | NA | IPR004360, IPR029068 | -1.84 | 0.93 | Down |
| *POX00610* | NA | NA | NA | NA | -1.39 | 0.93 | Down |
| *POX00634* | NA | NA | NA | IPR000192, IPR015421, IPR015424 | -1.46 | 0.90 | Down |
| *POX00653* | NA | NA | NA | NA | -1.04 | 0.84 | Down |
| *POX00656* | NA | NA | NA | IPR000719, IPR002290, IPR008271, IPR011009, IPR017441 | -1.67 | 0.92 | Down |
| *POX00665* | NA | NA | NA | NA | -2.44 | 0.97 | Down |
| *POX00666* | NA | NA | NA | NA | -1.92 | 0.85 | Down |
| *POX00700* | NA | NA | NA | IPR003000, IPR026590, IPR026591, IPR029035 | -1.09 | 0.87 | Down |
| *POX00702* | NA | NA | NA | IPR012349, IPR024747 | -2.33 | 0.94 | Down |
| *POX00706* | NA | NA | NA | IPR000719, IPR011009, IPR029498 | -1.06 | 0.82 | Down |
| *POX00713* | NA | NA | NA | IPR002575, IPR011009 | -2.21 | 0.85 | Down |
| *POX00714* | NA | NA | NA | IPR013112, IPR013121, IPR013130, IPR017927 | 1.54 | 0.92 | Up |
| *POX00718* | NA | NA | NA | NA | -1.75 | 0.93 | Down |
| *POX00759* | NA | β-mannosidase | GH2 | IPR006102, IPR008979, IPR013781, IPR013812, IPR017853 | -1.12 | 0.88 | Down |
| *POX00760* | NA | Pectinase | CE8 | IPR011050, IPR012334 | 6.22 | 0.93 | Up |
| *POX00761* | NA | NA | NA | IPR001017, IPR029061 | 2.79 | 0.91 | Up |
| *POX00769* | NA | NA | NA | IPR002872, IPR015659, IPR029041 | 2.75 | 0.94 | Up |
| *POX00775* | NA | NA | NA | IPR031606 | -1.30 | 0.88 | Down |

| | | | | | | | |
|---|---|---|---|---|---|---|---|
| *POX00795* | NA | NA | NA | IPR002129, IPR015421, IPR015422, IPR015424 | -1.54 | 0.93 | Down |
| *POX00834* | NA | NA | NA | NA | 2.35 | 0.85 | Up |
| *POX00835* | NA | NA | NA | IPR001272, IPR008210, IPR013035, IPR015994 | 1.55 | 0.95 | Up |
| *POX00836* | NA | NA | NA | IPR002067, IPR018108, IPR023395 | -1.20 | 0.91 | Down |
| *POX00859* | NA | NA | NA | IPR004360, IPR005956, IPR029068 | 6.02 | 0.95 | Up |
| *POX00860* | NA | NA | NA | NA | 3.98 | 0.91 | Up |
| *POX00863* | NA | NA | NA | IPR004045, IPR004046, IPR005955, IPR010987, IPR012336 | 3.79 | 0.81 | Up |
| *POX00867* | NA | NA | NA | NA | -1.89 | 0.94 | Down |
| *POX00870* | NA | NA | NA | IPR000120, IPR020556, IPR023631 | -2.21 | 0.90 | Down |
| *POX00879* | NA | NA | NA | IPR011021, IPR011022, IPR014756 | -1.00 | 0.85 | Down |
| *POX00884* | NA | NA | NA | IPR018712 | 2.56 | 0.82 | |
| *POX00914* | NA | NA | NA | IPR000210, IPR011333 | -1.76 | 0.92 | Down |
| *POX00917* | NA | NA | NA | IPR023753 | 1.43 | 0.92 | Up |
| *POX00919* | NA | NA | NA | IPR001279 | 4.31 | 0.98 | Up |
| *POX00920* | NA | NA | NA | IPR003807 | 3.03 | 0.95 | Up |
| *POX00923* | NA | β-xylosidase | GH43 | IPR006710, IPR016840, IPR023296 | 4.61 | 0.93 | Up |
| *POX00925* | NA | NA | NA | IPR011042, IPR013658 | 1.42 | 0.84 | Up |
| *POX00936* | Zn2Cys6 | NA | NA | IPR001138 | -1.32 | 0.90 | Down |
| *POX00945* | NA | NA | NA | IPR001128, IPR002401, IPR017972 | -1.11 | 0.91 | Down |
| *POX00960* | NA | NA | NA | IPR003663, IPR005828, IPR005829, IPR020846 | 11.30 | 0.95 | Up |
| *POX00968* | NA | β-glucosidase | GH3 | IPR001764, IPR002772, IPR017853, IPR026891, IPR026892 | -1.99 | 0.90 | Down |
| *POX00970* | NA | NA | GT32 | IPR007577, IPR029044 | 8.83 | 0.91 | Up |
| *POX00978* | NA | NA | NA | IPR000719, IPR002290, IPR011009 | 1.55 | 0.84 | Up |
| *POX00980* | NA | NA | NA | IPR002293, IPR004841 | 1.18 | 0.80 | Up |
| *POX00989* | NA | NA | NA | IPR006771 | 6.34 | 0.99 | Up |
| *POX00991* | NA | NA | NA | IPR000504, IPR012677 | -1.05 | 0.81 | Down |
| *POX00997* | NA | NA | NA | IPR005475, IPR009014, IPR029061 | 1.45 | 0.87 | Up |
| *POX01017* | NA | NA | NA | NA | -1.32 | 0.90 | Down |
| *POX01065* | NA | NA | NA | IPR018108, IPR023395 | 1.60 | 0.94 | Up |

| | | | | | | | |
|---|---|---|---|---|---|---|---|
| *POX01080* | NA | NA | NA | IPR000277, IPR006235, IPR015421, IPR015422, IPR015424 | -1.66 | 0.90 | Down |
| *POX01082* | NA | NA | NA | IPR002293, IPR004762, IPR004840, IPR004841 | -1.20 | 0.85 | Down |
| *POX01099* | NA | NA | CE1 | IPR000801, IPR014186, IPR029058 | -1.50 | 0.93 | Down |
| *POX01103* | NA | NA | NA | NA | 1.55 | 0.81 | Up |
| *POX01117* | NA | Feruloyl | NA | IPR011118, IPR029058, IPR031246 | -1.92 | 0.81 | Down |
| *POX01118* | Zn2Cys6 | NA | NA | IPR001138, IPR021858 | -1.44 | 0.88 | Down |
| *POX01120* | NA | NA | NA | IPR000832, IPR017981 | -1.36 | 0.84 | Down |
| *POX01158* | NA | NA | GH132 | IPR005556 | -1.02 | 0.92 | Down |
| *POX01166* | NA | Endo-β-1'4-glucanase | CBM1; GH5 | IPR000254, IPR001547, IPR013781, IPR017853 | -1.91 | 0.96 | Down |
| *POX01169* | NA | NA | NA | IPR002938, IPR023753 | 2.09 | 0.96 | Up |
| *POX01170* | NA | NA | NA | IPR001128, IPR002403 | 2.45 | 0.97 | Up |
| *POX01171* | NA | NA | NA | IPR002198, IPR002347, IPR016040, IPR020904 | 2.18 | 0.96 | Up |
| *POX01172* | NA | NA | NA | IPR009799, IPR011008 | 2.21 | 0.96 | Up |
| *POX01173* | NA | NA | NA | IPR025337 | 2.13 | 0.96 | Up |
| *POX01174* | NA | NA | NA | IPR008030, IPR016040 | 2.23 | 0.97 | Up |
| *POX01176* | NA | NA | NA | IPR004235, IPR032710 | 1.64 | 0.95 | Up |
| *POX01177* | NA | NA | NA | IPR001227, IPR009081, IPR014030, IPR014031, IPR014043, IPR016035, IPR016036, IPR016039, IPR020801, IPR020806, IPR020807, IPR020841, IPR030918, IPR032088 | 2.07 | 0.96 | Up |
| *POX01178* | NA | NA | NA | IPR001279 | 2.04 | 0.96 | Up |
| *POX01179* | NA | NA | NA | IPR002198, IPR002347, IPR013968, IPR016040, IPR020904 | 1.74 | 0.96 | Up |
| *POX01180* | NA | NA | NA | , IPR032710 | 1.94 | 0.96 | Up |
| *POX01181* | NA | NA | NA | IPR032710 | 1.68 | 0.96 | Up |
| *POX01182* | NA | NA | NA | IPR013216, IPR029063 | 2.47 | 0.97 | Up |
| *POX01183* | Zn2Cys6 | NA | NA | IPR001138, IPR002409, IPR013700 | 1.26 | 0.90 | Up |
| *POX01188* | NA | NA | NA | NA | -3.76 | 0.95 | Down |

| | | | | | | | |
|---|---|---|---|---|---|---|---|
| *POX01189* | NA | NA | GH47 | IPR001382 | -2.78 | 0.96 | Down |
| *POX01190* | NA | NA | NA | IPR000719, IPR002290, IPR011009 | 1.56 | 0.87 | Up |
| *POX01194* | NA | NA | NA | IPR004045, IPR004046, IPR010987, IPR012336 | 3.73 | 0.90 | Up |
| *POX01201* | NA | NA | NA | IPR005828, IPR020846 | -1.43 | 0.89 | Down |
| *POX01218* | NA | Acetyl | CBM1; CE1 | IPR000254, IPR010126, IPR029058 | -2.14 | 0.94 | Down |
| *POX01225* | NA | Polygalacturo nase; | GH28 | IPR000743, IPR011050, IPR012334 | 2.80 | 0.94 | Up |
| *POX01262* | NA | NA | NA | IPR002293 | 2.17 | 0.80 | Up |
| *POX01268* | NA | NA | NA | IPR021134 | -2.32 | 0.95 | Down |
| *POX01317* | NA | NA | NA | IPR003663, IPR005828, IPR005829, IPR020846 | -1.80 | 0.96 | Down |
| *POX01320* | NA | NA | NA | IPR002198, IPR002347, IPR016040 | -2.29 | 0.87 | Down |
| *POX01356* | NA | NA | CBM20; GH15 | IPR000165, IPR002044, IPR008291, IPR008928, IPR011613, IPR012341, IPR013783, IPR013784 | 2.28 | 0.96 | Up |
| *POX01369* | NA | NA | GT41 | IPR011990, IPR013026, IPR019734, IPR027417 | -1.52 | 0.93 | Down |
| *POX01379* | NA | NA | NA | IPR004839, IPR015421, IPR015422, IPR015424 | -3.52 | 0.82 | Down |
| *POX01390* | NA | NA | CE1 | IPR010520, IPR029058 | 3.01 | 0.97 | Up |
| *POX01391* | NA | NA | AA1 | IPR001117, IPR002355, IPR008972, IPR011706, IPR011707 | 2.97 | 0.97 | Up |
| *POX01413* | NA | NA | NA | IPR001338 | 5.97 | 0.98 | Up |
| *POX01419* | NA | NA | NA | NA | -1.44 | 0.93 | Down |
| *POX01430* | NA | NA | NA | IPR001031, IPR001227, IPR006162, IPR009081, IPR014030, IPR014031, IPR014043, IPR016035, IPR016036, IPR016039, IPR018201, IPR020801, IPR020806, IPR020807, IPR020841, IPR029058, IPR030918, IPR032088 | 5.08 | 0.98 | Up |
| *POX01431* | NA | NA | AA1 | IPR001117, IPR002355, IPR008972, IPR011706, IPR011707 | 3.66 | 0.97 | Up |

| | | | | | | | |
|---|---|---|---|---|---|---|---|
| *POX01447* | NA | NA | GH18 | IPR001223, IPR001579, IPR013781, IPR017853 | 5.75 | 0.97 | Up |
| *POX01460* | NA | NA | NA | IPR001251, IPR011074 | -2.13 | 0.94 | Down |
| *POX01469* | NA | NA | NA | IPR003673, IPR023606 | 1.73 | 0.90 | Up |
| *POX01470* | NA | NA | GH18 | IPR001223, IPR001579, IPR013781, IPR017853 | -1.97 | 0.95 | Down |
| *POX01476* | NA | NA | NA | NA | 3.41 | 0.94 | Up |
| *POX01481* | NA | NA | NA | IPR018946, IPR029052 | 1.49 | 0.82 | Up |
| *POX01489* | NA | NA | NA | NA | 2.11 | 0.81 | Up |
| *POX01507* | NA | NA | NA | IPR025256 | 1.26 | 0.84 | Up |
| *POX01513* | NA | NA | NA | NA | 1.31 | 0.90 | Up |
| *POX01524* | NA | Expansin-like | CBM63 | IPR007112, IPR007117, IPR009009 | -1.79 | 0.86 | Down |
| *POX01533* | NA | NA | NA | NA | 1.35 | 0.85 | Up |
| *POX01536* | NA | NA | NA | NA | 2.46 | 0.95 | Up |
| *POX01566* | NA | NA | NA | IPR003663, IPR005828, IPR020846 | 7.59 | 1.00 | Up |
| *POX01576* | NA | NA | NA | IPR012469 | -1.36 | 0.83 | Down |
| *POX01603* | NA | NA | NA | NA | -1.76 | 0.91 | Down |
| *POX01604* | NA | NA | NA | NA | -1.97 | 0.93 | Down |
| *POX01612* | NA | NA | NA | IPR007065 | -2.07 | 0.94 | Down |
| *POX01614* | NA | NA | NA | NA | -2.75 | 0.92 | Down |
| *POX01615* | NA | NA | GT69 | IPR021047 | 4.38 | 0.93 | Up |
| *POX01633* | NA | NA | NA | NA | -1.87 | 0.90 | Down |
| *POX01636* | NA | NA | NA | IPR002110, IPR020683 | -2.29 | 0.96 | Down |
| *POX01646* | NA | β-xylosidase | GH3 | IPR001764, IPR002772, IPR017853, IPR026891, IPR026892 | -1.57 | 0.86 | Down |
| *POX01649* | NA | NA | NA | IPR005828, IPR020846 | 3.65 | 0.94 | Up |
| *POX01651* | NA | NA | NA | IPR001338 | 1.37 | 0.94 | Up |
| *POX01682* | NA | NA | NA | IPR001128, IPR002401, IPR017972 | 1.67 | 0.92 | Up |
| *POX01685* | NA | NA | NA | IPR001509, IPR016040 | -1.14 | 0.88 | Down |
| *POX01690* | NA | NA | NA | IPR000022, IPR011762, IPR011763, IPR029045 | 1.44 | 0.88 | Down |
| *POX01691* | NA | NA | NA | IPR006089, IPR006091, IPR009075, IPR009100, IPR013786 | 1.40 | 0.88 | Up |
| *POX01700* | NA | NA | NA | IPR003663, IPR005828, IPR005829, IPR020846 | -1.24 | 0.90 | Down |

| | | | | | | | |
|---|---|---|---|---|---|---|---|
| *POX01702* | NA | NA | NA | IPR000089, IPR005479, IPR005481, IPR005482, IPR011053, IPR011054, IPR011761, IPR011764, IPR013815, IPR013816, IPR016185 | 1.41 | 0.88 | Up |
| *POX01704* | NA | NA | NA | NA | -5.20 | 0.88 | Down |
| *POX01713* | NA | NA | NA | NA | -1.91 | 0.95 | Down |
| *POX01717* | NA | NA | NA | NA | -1.76 | 0.93 | Down |
| *POX01718* | NA | NA | NA | NA | -2.06 | 0.86 | Down |
| *POX01720* | NA | NA | NA | NA | -2.26 | 0.85 | Down |
| *POX01761* | NA | NA | NA | IPR004136, IPR013785 | 1.50 | 0.93 | Up |
| *POX01762* | NA | NA | NA | NA | 1.24 | 0.91 | Up |
| *POX01763* | NA | NA | NA | NA | -2.30 | 0.93 | Down |
| *POX01764* | NA | NA | NA | IPR001338, IPR019778 | 2.78 | 0.97 | Up |
| *POX01787* | NA | NA | NA | NA | 1.74 | 0.92 | Up |
| *POX01789* | NA | NA | NA | NA | 4.02 | 0.89 | Up |
| *POX01797* | NA | NA | NA | IPR018535 | 1.63 | 0.90 | Up |
| *POX01798* | NA | NA | NA | IPR001395, IPR005399, IPR023210 | -2.05 | 0.94 | Down |
| *POX01819* | NA | NA | NA | NA | 2.25 | 0.93 | Up |
| *POX01825* | NA | NA | GT90 | IPR006598 | -1.34 | 0.86 | Down |
| *POX01826* | NA | NA | NA | IPR011058 | -2.11 | 0.85 | Down |
| *POX01831* | NA | NA | GT3; GT5 | IPR008631 | -1.19 | 0.90 | Down |
| *POX01843* | NA | NA | NA | IPR011701, IPR020846 | 1.66 | 0.89 | Up |
| *POX01851* | NA | NA | NA | IPR005913, IPR016040, IPR029903 | 1.14 | 0.90 | Up |
| *POX01862* | NA | NA | NA | IPR006076, IPR023753 | 1.94 | 0.84 | Up |
| *POX01864* | NA | NA | NA | IPR021838 | 5.12 | 0.94 | Up |
| *POX01867* | NA | NA | NA | IPR008701 | -1.17 | 0.82 | Down |
| *POX01875* | NA | NA | NA | IPR002921, IPR005592, IPR029058 | 1.36 | 0.89 | Up |
| *POX01886* | NA | NA | NA | NA | 5.60 | 0.96 | Up |
| *POX01896* | NA | Endo-β-1'4-glucanase | CBM1; CBM46; GH5 | IPR000254, IPR001547, IPR005102, IPR013781, IPR013783, IPR014756, IPR017853 | -1.12 | 0.92 | Down |
| *POX01897* | NA | NA | NA | IPR003864, IPR022257, IPR027815, IPR032880 | 2.04 | 0.92 | Up |
| *POX01917* | NA | NA | AA7 | IPR006094, IPR012951, IPR016166, IPR016167, IPR016169 | -1.41 | 0.83 | Down |

| | | | | | | | |
|---|---|---|---|---|---|---|---|
| *POX01920* | NA | NA | NA | IPR003663, IPR005828, IPR005829, IPR020846 | -1.45 | 0.91 | Down |
| *POX08747* | NA | NA | NA | IPR017946 | -1.36 | 0.91 | Down |
| *POX08753* | NA | NA | NA | IPR001128, IPR002401, IPR017972 | 1.99 | 0.95 | Up |
| *POX08754* | NA | NA | NA | IPR021838 | 6.78 | 0.95 | Up |
| *POX08755* | NA | NA | NA | NA | 10.31 | 0.97 | Up |
| *POX08758* | NA | NA | AA3; AA8 | IPR000172, IPR007867, IPR012132, IPR023753, IPR027424 | 9.95 | 0.99 | Up |
| *POX08763* | NA | NA | NA | NA | 3.23 | 0.95 | Up |
| *POX08766* | NA | NA | NA | IPR000182, IPR016181 | 1.20 | 0.91 | Up |
| *POX08768* | NA | NA | GH65 | IPR005195, IPR005196, IPR008928, IPR011013, IPR012341 | -1.33 | 0.92 | Down |
| *POX08776* | NA | NA | NA | IPR001128, IPR002401, IPR017972 | 2.28 | 0.95 | Up |
| *POX08780* | NA | NA | NA | IPR003703, IPR029069 | -1.93 | 0.89 | Down |
| *POX08786* | NA | NA | NA | IPR003437, IPR015421, IPR015424, IPR020580, IPR020581 | 1.87 | 0.81 | Up |
| *POX08788* | NA | NA | NA | IPR032710 | -1.82 | 0.92 | Down |
| *POX08789* | NA | NA | NA | IPR000182, IPR016181 | -2.62 | 0.95 | Down |
| *POX08797* | NA | NA | NA | IPR007312 | 3.06 | 0.92 | Up |
| *POX08806* | NA | NA | NA | NA | 2.33 | 0.96 | Up |
| *POX08807* | NA | NA | NA | IPR022703 | 2.95 | 0.95 | Up |
| *POX08813* | NA | NA | CBM20; AA13 | IPR002044, IPR004302, IPR013783, IPR013784 | 2.82 | 0.93 | Up |
| *POX08814* | NA | NA | NA | IPR000209, IPR009020, IPR015366, IPR030400 | 3.13 | 0.92 | Up |
| *POX08819* | NA | NA | NA | IPR011598 | -1.85 | 0.96 | Down |
| *POX08822* | NA | NA | NA | IPR000971, IPR001433, IPR008333, IPR009050, IPR012292, IPR017927, IPR017938 | -2.48 | 0.97 | Down |
| *POX08824* | NA | NA | NA | IPR000425, IPR023271 | 5.98 | 0.92 | Up |
| *POX08827* | NA | NA | GH55 | IPR011050, IPR012334, IPR024429, IPR024535 | 6.91 | 0.93 | Up |
| *POX08828* | NA | NA | NA | IPR000873, IPR020845 | 2.51 | 0.92 | Up |
| *POX08829* | NA | NA | NA | IPR011701, IPR020846 | 2.55 | 0.82 | Up |
| *POX08833* | NA | NA | NA | IPR007632, IPR030142 | -1.29 | 0.86 | Down |

| | | | | | | | |
|---|---|---|---|---|---|---|---|
| *POX08834* | NA | NA | NA | IPR004837 | 1.98 | 0.91 | Up |
| *POX08835* | NA | NA | NA | IPR001202 | -1.15 | 0.93 | Down |
| *POX08844* | NA | NA | NA | IPR001806, IPR003578, IPR003579, IPR005225, IPR020849, IPR027417 | 1.93 | 0.93 | Up |
| *POX08877* | NA | NA | NA | NA | -1.49 | 0.90 | Down |
| *POX08881* | NA | NA | GH105; GH76 | IPR002328, IPR005198, IPR008928, IPR011032, IPR012341, IPR013149, IPR013154, IPR016040, IPR020843 | 3.74 | 0.92 | Up |
| *POX08885* | NA | NA | NA | NA | 6.95 | 1.00 | Up |
| *POX08890* | NA | NA | NA | IPR028018 | -2.50 | 0.94 | Down |
| *POX08897* | NA | Cellulose | CBM1; AA9 | IPR000254, IPR005103 | -2.60 | 0.95 | Down |
| *POX08904* | NA | NA | NA | IPR000073, IPR029058 | -1.68 | 0.92 | Down |
| *POX08906* | NA | NA | NA | NA | -1.88 | 0.96 | Down |
| *POX08912* | NA | NA | NA | IPR008754, IPR024079 | 5.88 | 0.99 | Up |
| *POX08929* | NA | NA | NA | IPR013216, IPR029063 | -1.34 | 0.83 | Down |
| *POX08943* | NA | NA | NA | IPR001260, IPR018375 | -1.41 | 0.91 | Down |
| *POX08946* | NA | NA | NA | NA | -2.73 | 0.97 | Down |
| *POX08954* | NA | NA | NA | IPR008979 | 1.07 | 0.89 | Up |
| *POX08970* | NA | NA | NA | IPR002293, IPR004840 | -1.72 | 0.81 | Down |
| *POX09006* | NA | NA | NA | IPR004045, IPR010987, IPR012336 | -1.21 | 0.86 | Down |
| *POX09025* | NA | NA | NA | IPR000898 | -2.51 | 0.96 | Down |
| *POX09026* | NA | NA | NA | IPR000192, IPR010111, IPR015421, IPR015422, IPR015424 | -1.04 | 0.89 | Down |
| *POX09040* | NA | NA | NA | NA | -2.82 | 0.96 | Down |
| *POX09081* | NA | NA | NA | IPR000719, IPR002290, IPR003527, IPR008271, IPR011009 | 1.55 | 0.85 | Up |
| *POX09083* | Zn2Cys6 | NA | NA | IPR001138, IPR007219 | 1.16 | 0.88 | Up |
| *POX09084* | NA | NA | NA | IPR005828, IPR020846 | -2.42 | 0.94 | Down |
| *POX09085* | NA | α-galactosidase | CBM1; GH27; GH36 | IPR000254, IPR002241, IPR006215, IPR013780, IPR013785, IPR017853 | -3.68 | 0.94 | Down |
| *POX09101* | NA | NA | NA | NA | -1.75 | 0.94 | Down |
| *POX09110* | NA | NA | CBM24; GH71 | IPR005197 | 4.57 | 0.95 | Up |
| *POX09117* | NA | NA | NA | IPR013022 | 1.83 | 0.81 | Up |
| *POX09120* | NA | NA | GH16 | IPR000757, IPR013320 | 1.38 | 0.85 | Up |
| *POX09124* | Zn2Cys6 | NA | NA | IPR001138 | 2.54 | 0.94 | Up |
| *POX09137* | NA | Feruloyl | CBM1; CE1 | IPR000254, IPR001375, IPR029058 | -1.30 | 0.90 | Down |

| | | | | | | | |
|---|---|---|---|---|---|---|---|
| *POX09138* | NA | NA | NA | IPR001763 | 4.87 | 0.98 | Up |
| *POX09139* | Zn2Cys6 | NA | NA | IPR007219 | 2.49 | 0.92 | Up |
| *POX09154* | NA | β-xylosidase | GH43 | IPR006710, IPR016840, IPR023296 | 2.14 | 0.88 | Up |
| *POX09164* | NA | NA | NA | NA | 5.05 | 0.89 | Up |
| *POX09166* | NA | NA | NA | NA | -3.28 | 0.95 | Down |
| *POX09167* | NA | NA | NA | IPR002498, IPR016034, IPR023610, IPR027483, IPR027484 | 1.05 | 0.90 | Up |
| *POX09169* | NA | NA | NA | IPR003439, IPR003593, IPR013525, IPR017871, IPR027417 | 2.42 | 0.96 | Up |
| *POX09181* | NA | NA | NA | IPR021740 | 1.96 | 0.94 | Up |
| *POX09187* | HMG | NA | NA | IPR009071 | -1.23 | 0.84 | Down |
| *POX09195* | NA | NA | NA | IPR001128, IPR010255, IPR017972, IPR019791 | 1.43 | 0.85 | Up |
| *POX09199* | NA | NA | GT34 | IPR008630 | 1.22 | 0.84 | Up |
| *POX09204* | NA | NA | NA | NA | 4.43 | 0.98 | Up |
| *POX09217* | NA | NA | NA | NA | 1.04 | 0.89 | Up |
| *POX09222* | NA | NA | NA | IPR023753 | 2.07 | 0.91 | Up |
| *POX09233* | NA | NA | AA3; AA8 | IPR000172, IPR007867, IPR012132, IPR023753 | 2.93 | 0.96 | Up |
| *POX09240* | NA | NA | NA | IPR005804 | 3.41 | 0.89 | Up |
| *POX09284* | NA | NA | NA | NA | -2.82 | 0.93 | Down |
| *POX09287* | NA | NA | NA | NA | -1.10 | 0.93 | Down |
| *POX09289* | NA | NA | NA | IPR022234 | -1.98 | 0.96 | Down |
| *POX09301* | NA | NA | NA | NA | -1.46 | 0.92 | Down |
| *POX09330* | NA | NA | NA | IPR002110, IPR020683 | -1.34 | 0.90 | Down |
| *POX09332* | NA | NA | NA | IPR001227, IPR002539, IPR003965, IPR013565, IPR013785, IPR014043, IPR016035, IPR016452, IPR020801, IPR029069 | -1.19 | 0.90 | Down |
| *POX09333* | NA | NA | NA | IPR029069 | -1.27 | 0.85 | Down |
| *POX09334* | NA | NA | NA | IPR003439, IPR003593, IPR011527, IPR017871, IPR027417 | -1.71 | 0.91 | Down |
| *POX09335* | NA | NA | NA | IPR001227, IPR014030, IPR014031, IPR016035, IPR016039, IPR016040, IPR018201 | -1.54 | 0.90 | Down |
| *POX09336* | NA | NA | NA | IPR001128, IPR002401, IPR017972 | -1.59 | 0.93 | Down |

| | | | | | | | |
|---|---|---|---|---|---|---|---|
| *POX09337* | NA | NA | NA | IPR001544, IPR005786 | -1.18 | 0.91 | Down |
| *POX09340* | NA | NA | NA | IPR000873, IPR001242, IPR006162, IPR009081, IPR010071, IPR020806, IPR020845, IPR025110 | -1.50 | 0.83 | Down |
| *POX09345* | NA | NA | NA | IPR018809 | 1.19 | 0.93 | Up |
| *POX09351* | NA | NA | NA | IPR002085, IPR002328, IPR011032, IPR013149, IPR013154, IPR016040, IPR020843 | 1.72 | 0.84 | Up |
| *POX09352* | NA | NA | CBM20; GH13 | IPR002044, IPR006047, IPR006589, IPR013780, IPR013781, IPR013783, IPR013784, IPR015340, IPR015902, IPR017853 | 1.74 | 0.92 | Up |
| *POX09354* | NA | NA | NA | NA | -5.93 | 0.96 | Down |
| *POX09355* | NA | NA | NA | IPR001128, IPR002401 | -5.30 | 0.98 | Down |
| *POX09357* | NA | NA | NA | IPR006905, IPR023753 | -2.20 | 0.82 | Down |
| *POX09358* | NA | NA | NA | IPR004045, IPR004046, IPR010987, IPR012336 | -7.00 | 1.00 | Down |
| *POX09359* | NA | NA | NA | IPR007817 | -2.21 | 0.81 | Down |
| *POX09360* | NA | NA | NA | NA | -2.19 | 0.85 | Down |
| *POX09361* | NA | NA | NA | IPR002818, IPR029062 | -4.70 | 0.98 | Down |
| *POX09365* | NA | NA | NA | IPR011701, IPR020846 | -3.79 | 0.98 | Down |
| *POX09367* | NA | NA | NA | IPR000873, IPR010071, IPR013120, IPR016040, IPR020845, IPR025110 | 10.28 | 0.97 | Up |
| *POX09368* | NA | NA | NA | IPR002129, IPR015421, IPR015424 | 9.97 | 0.99 | Up |
| *POX09375* | NA | NA | NA | NA | -1.70 | 0.91 | Down |
| *POX09377* | NA | NA | NA | IPR009784, IPR013320 | -1.29 | 0.91 | Down |
| *POX09381* | NA | NA | NA | IPR006035, IPR020855, IPR023696 | 1.88 | 0.86 | Up |
| *POX09387* | NA | NA | GH18 | IPR001223, IPR013781, IPR017853 | 2.18 | 0.82 | Up |
| *POX09389* | NA | NA | NA | IPR002085, IPR011032, IPR016040, IPR020843 | -1.29 | 0.87 | Down |
| *POX09405* | NA | NA | NA | NA | 6.88 | 0.99 | Up |
| *POX09406* | NA | NA | NA | NA | 11.87 | 0.99 | Up |
| *POX09412* | NA | NA | NA | IPR000073, IPR029058 | 1.09 | 0.81 | Up |
| *POX09413* | NA | NA | NA | IPR001810 | 2.63 | 0.95 | Up |
| *POX09419* | NA | NA | NA | NA | 5.27 | 0.96 | Up |

| | | | | | | | |
|---|---|---|---|---|---|---|---|
| *POX09440* | NA | NA | NA | IPR006139, IPR006140, IPR016040, IPR029752, IPR029753 | -2.87 | 0.97 | Down |
| *POX09463* | NA | NA | NA | IPR005630, IPR008949 | 2.47 | 0.89 | Up |
| *POX09464* | NA | NA | NA | IPR001128, IPR002401 | 2.22 | 0.89 | Up |
| *POX09465* | NA | NA | NA | IPR029063 | 1.75 | 0.86 | Up |
| *POX09467* | NA | NA | NA | IPR000873, IPR001242, IPR006162, IPR009081, IPR010071, IPR020806, IPR020845 | 5.28 | 0.93 | Up |
| *POX09468* | NA | NA | NA | IPR001128, IPR017972 | 3.73 | 0.95 | Up |
| *POX09469* | Winged helix repressor DNA-binding | NA | NA | IPR001077, IPR011991, IPR016461, IPR029063 | 1.14 | 0.91 | Up |
| *POX09470* | NA | NA | NA | IPR002938, IPR003953, IPR023753 | 2.91 | 0.97 | Up |
| *POX09471* | NA | NA | NA | IPR005829, IPR011701, IPR020846 | 4.09 | 0.95 | Up |
| *POX09472* | NA | NA | NA | IPR012148, IPR017795 | 2.20 | 0.96 | Up |
| *POX09473* | NA | NA | NA | IPR013216, IPR029063 | 1.91 | 0.95 | Up |
| *POX09474* | NA | NA | NA | IPR001128, IPR002403, IPR017972 | 2.62 | 0.92 | Up |
| *POX09477* | NA | NA | NA | IPR009737, IPR012336, IPR030108 | 1.16 | 0.83 | Up |
| *POX09521* | NA | NA | NA | IPR006694 | -1.11 | 0.90 | Down |
| *POX09523* | NA | NA | NA | IPR001461, IPR001969, IPR021109 | -1.22 | 0.91 | Down |
| *POX09546* | NA | NA | NA | IPR001680, IPR015943, IPR017986, IPR019775, IPR020472 | -2.44 | 0.95 | Down |
| *POX09559* | NA | NA | NA | IPR002110, IPR020683 | -1.28 | 0.89 | Down |
| *POX09563* | NA | NA | NA | NA | -3.58 | 0.91 | Down |
| *POX09578* | NA | NA | CE10; CE1 | IPR013094, IPR029058 | -1.70 | 0.87 | Down |
| *POX09615* | NA | NA | NA | NA | -4.44 | 0.81 | Down |
| *POX09620* | NA | NA | NA | NA | 1.71 | 0.88 | Up |
| *POX09621* | NA | NA | GH18 | IPR001223, IPR001579, IPR011583, IPR013781, IPR017853, IPR029070 | 7.06 | 0.95 | Up |
| *POX09677* | NA | NA | NA | NA | -1.44 | 0.84 | Down |
| *POX09678* | NA | NA | NA | NA | 5.37 | 0.86 | Up |
| *POX09684* | NA | NA | NA | IPR000953, IPR016197, IPR023780 | 1.28 | 0.89 | Up |
| *POX09687* | NA | NA | NA | NA | 1.85 | 0.87 | Up |
| *POX09688* | NA | NA | NA | NA | 3.55 | 0.86 | Up |

| | | | | | | | |
|---|---|---|---|---|---|---|---|
| *POX09690* | NA | NA | NA | NA | 3.60 | 0.84 | Up |
| *POX09711* | NA | NA | NA | NA | 3.64 | 0.86 | Up |
| *POX09716* | NA | NA | NA | NA | -2.31 | 0.81 | Down |
| *POX09717* | NA | NA | NA | NA | -3.19 | 0.94 | Down |
| *POX09718* | NA | NA | NA | NA | -3.41 | 0.82 | Down |
| *POX09719* | NA | NA | NA | NA | -5.33 | 0.93 | Down |
| *POX09759* | NA | NA | NA | NA | -1.04 | 0.92 | Down |
| *POX09766* | NA | NA | NA | IPR016491, IPR027417, IPR030379 | -1.15 | 0.92 | Down |
| *POX09776* | NA | NA | NA | IPR001680, IPR007111, IPR015943, IPR017986, IPR019775, IPR020472, IPR027417, IPR029498 | -2.71 | 0.94 | Down |
| *POX09786* | NA | NA | NA | NA | -7.36 | 0.88 | Down |
| *POX09798* | NA | NA | NA | IPR021264 | -5.07 | 0.90 | Down |
| *POX01926* | NA | NA | NA | NA | 1.50 | 0.91 | Up |
| *POX01927* | NA | NA | NA | IPR008701 | 1.30 | 0.87 | Up |
| *POX01929* | NA | NA | NA | IPR001563, IPR018202, IPR029058 | 5.19 | 0.94 | Up |
| *POX01937* | NA | β-1'4-mannanase | CBM1; GH5 | IPR000254, IPR001547, IPR013781, IPR017853 | -1.36 | 0.94 | Down |
| *POX01940* | NA | NA | NA | IPR002085, IPR002328, IPR011032, IPR013149, IPR013154, IPR016040, IPR020843 | 4.19 | 0.90 | Up |
| *POX01946* | NA | NA | NA | IPR000250, IPR013320 | 5.26 | 0.95 | |
| *POX01960* | Zn2Cys6 | NA | NA | IPR001138, IPR007219 | -1.34 | 0.89 | Down |
| *POX01961* | NA | β-mannosidase | GH2 | IPR006102, IPR006104, IPR008979, IPR013781, IPR013812, IPR017853 | -1.48 | 0.93 | Down |
| *POX01979* | NA | NA | NA | IPR001128, IPR002403 | 6.05 | 0.81 | Up |
| *POX01981* | NA | NA | NA | IPR000109, IPR020846 | 2.69 | 0.92 | Up |
| *POX01986* | NA | NA | AA7 | IPR006094, IPR016166, IPR016169 | -2.70 | 0.91 | Down |
| *POX01987* | NA | NA | NA | NA | -3.29 | 0.90 | Down |
| *POX01989* | NA | NA | NA | IPR011701, IPR020846 | 1.61 | 0.82 | Up |
| *POX01998* | NA | NA | NA | IPR013320 | 7.45 | 0.96 | Up |
| *POX02006* | NA | NA | NA | IPR001447 | -1.94 | 0.85 | Down |
| *POX02008* | NA | NA | NA | NA | -2.50 | 0.97 | Down |
| *POX02009* | NA | NA | NA | NA | -2.95 | 0.96 | Down |
| *POX02010* | NA | NA | NA | NA | 8.97 | 0.97 | Up |

| | | | | | | | |
|---|---|---|---|---|---|---|---|
| *POX02011* | NA | NA | NA | IPR000210, IPR011333 | -2.42 | 0.92 | Down |
| *POX02024* | NA | Polygalacturo nase; | GH28 | IPR000743, IPR011050, IPR012334 | -3.25 | 0.85 | Down |
| *POX02026* | NA | NA | NA | NA | -3.81 | 0.92 | Down |
| *POX02044* | NA | NA | NA | IPR011021 | -1.07 | 0.92 | Down |
| *POX02048* | NA | NA | NA | IPR000898 | 1.32 | 0.83 | Down |
| *POX02059* | Zn2Cys6 | NA | NA | IPR001138, IPR002409, IPR021858 | -1.16 | 0.81 | Down |
| *POX02066* | NA | NA | NA | IPR017795 | -2.34 | 0.96 | Down |
| *POX02067* | NA | NA | NA | NA | -1.31 | 0.93 | Down |
| *POX02068* | NA | NA | NA | IPR001128, IPR002401, IPR017972 | -1.90 | 0.94 | Down |
| *POX02069* | NA | NA | NA | IPR000898, IPR001199, IPR018506 | -1.65 | 0.93 | Down |
| *POX02080* | NA | NA | NA | NA | 1.82 | 0.92 | Up |
| *POX02101* | NA | NA | NA | IPR000836, IPR005764, IPR029057 | -1.20 | 0.92 | Down |
| *POX02111* | NA | NA | NA | IPR025676 | -1.17 | 0.83 | Down |
| *POX02123* | NA | NA | NA | NA | 1.84 | 0.92 | Up |
| *POX02133* | NA | NA | NA | NA | -1.90 | 0.94 | Down |
| *POX02151* | NA | NA | NA | IPR001810 | -1.05 | 0.85 | Down |
| *POX02167* | NA | NA | NA | NA | 2.09 | 0.90 | Up |
| *POX02197* | NA | NA | NA | NA | -1.93 | 0.89 | Down |
| *POX02210* | NA | NA | NA | IPR002048, IPR011992, IPR018247 | 2.09 | 0.89 | Up |
| *POX02211* | NA | NA | NA | IPR015075, IPR023393 | 1.23 | 0.92 | Up |
| *POX02214* | NA | NA | NA | IPR006108, IPR006176, IPR006180, IPR008927, IPR013328, IPR016040, IPR022694 | 1.25 | 0.90 | Up |
| *POX02220* | NA | NA | NA | IPR001374, IPR025952 | 1.63 | 0.92 | Up |
| *POX02221* | NA | NA | NA | NA | 1.15 | 0.84 | Up |
| *POX02229* | NA | NA | NA | IPR016624 | -1.50 | 0.89 | Down |
| *POX02231* | NA | NA | NA | IPR013920 | -1.96 | 0.96 | Down |
| *POX02243* | NA | NA | NA | NA | -1.40 | 0.91 | Down |
| *POX02282* | NA | NA | NA | NA | 2.25 | 0.96 | Up |
| *POX02283* | NA | NA | NA | IPR008030, IPR016040 | 1.91 | 0.88 | Up |
| *POX02291* | NA | NA | NA | NA | 2.00 | 0.93 | Up |
| *POX02308* | NA | Cellulose | AA9 | IPR005103 | -2.29 | 0.96 | Down |
| *POX02319* | NA | NA | NA | NA | -1.93 | 0.91 | Down |
| *POX02336* | NA | NA | NA | IPR008253 | 1.70 | 0.94 | Up |

| | | | | | | | |
|---|---|---|---|---|---|---|---|
| *POX02345* | NA | α-galactosidase | GH36 | IPR001412, IPR002305, IPR002307, IPR002942, IPR008811, IPR013785, IPR014729, IPR017853, IPR032005 | -1.18 | 0.84 | Down |
| *POX02346* | NA | NA | NA | IPR001650, IPR011545, IPR014001, IPR027417 | -4.43 | 0.92 | Down |
| *POX02352* | NA | NA | NA | NA | 3.46 | 0.96 | Up |
| *POX02353* | NA | NA | AA7 | IPR006094, IPR012951, IPR016166, IPR016167, IPR016169 | 3.31 | 0.90 | Up |
| *POX02360* | NA | NA | NA | NA | -2.19 | 0.96 | Down |
| *POX02361* | NA | NA | NA | NA | -1.42 | 0.91 | Down |
| *POX02367* | NA | NA | NA | IPR002641, IPR016035, IPR021771 | 2.31 | 0.93 | Up |
| *POX02383* | NA | NA | NA | IPR016040 | 1.41 | 0.87 | Up |
| *POX02395* | NA | NA | NA | IPR000560, IPR029033 | -1.62 | 0.91 | Down |
| *POX02397* | NA | NA | NA | NA | 3.28 | 0.91 | Up |
| *POX02414* | NA | NA | NA | NA | 2.45 | 0.96 | Up |
| *POX02415* | NA | NA | NA | IPR002293, IPR004840 | 2.00 | 0.89 | Up |
| *POX02423* | NA | NA | GH64 | IPR032477 | 5.60 | 0.98 | Up |
| *POX02424* | NA | NA | NA | NA | 1.11 | 0.89 | Up |
| *POX02429* | NA | NA | NA | IPR006073, IPR012971, IPR023179, IPR024929, IPR027417, IPR030378 | -1.18 | 0.88 | Down |
| *POX02448* | NA | NA | NA | NA | -3.84 | 0.89 | Down |
| *POX02452* | C2H2 zinc finger | NA | NA | IPR007087, IPR013087, IPR015880 | -2.47 | 0.95 | Down |
| *POX02461* | NA | NA | NA | IPR000192, IPR005302, IPR005303, IPR015421, IPR015422, IPR015424, IPR028886 | 1.83 | 0.92 | Up |
| *POX02473* | NA | NA | NA | IPR011701, IPR020846 | 2.78 | 0.95 | Up |
| *POX02474* | NA | NA | NA | NA | 2.66 | 0.93 | Up |
| *POX02475* | NA | NA | NA | NA | 7.41 | 1.00 | Up |
| *POX02476* | NA | NA | NA | NA | 2.06 | 0.93 | Up |
| *POX02478* | NA | NA | NA | NA | -1.14 | 0.81 | Down |
| *POX02480* | NA | NA | NA | IPR011021, IPR011022, IPR014756 | -1.18 | 0.88 | Down |
| *POX02483* | NA | NA | NA | NA | -1.05 | 0.91 | Down |
| *POX02485* | NA | NA | NA | IPR001660, IPR001849, IPR011993, IPR013761 | -1.82 | 0.94 | Down |

| | | | | | | | |
|---|---|---|---|---|---|---|---|
| *POX02488* | NA | NA | NA | NA | 1.20 | 0.86 | Up |
| *POX02502* | NA | NA | NA | NA | -3.66 | 0.81 | Down |
| *POX02519* | NA | NA | NA | IPR028241 | 1.40 | 0.89 | Up |
| *POX02553* | NA | NA | NA | IPR004813 | -1.94 | 0.93 | Down |
| *POX02564* | NA | NA | NA | NA | -1.22 | 0.87 | Down |
| *POX02577* | NA | NA | NA | NA | 1.33 | 0.91 | Up |
| *POX02578* | NA | NA | NA | NA | 1.07 | 0.88 | Up |
| *POX02594* | NA | NA | NA | NA | -1.78 | 0.95 | Down |
| *POX02595* | NA | NA | NA | NA | -3.33 | 0.91 | Down |
| *POX02597* | NA | NA | NA | NA | -2.10 | 0.84 | Down |
| *POX02600* | NA | NA | NA | IPR022036 | 3.89 | 0.93 | Up |
| *POX02610* | NA | NA | NA | NA | 2.04 | 0.85 | Up |
| *POX02623* | NA | NA | NA | IPR000132, IPR003010 | -1.69 | 0.84 | Down |
| *POX02628* | NA | NA | NA | IPR001563, IPR018202, IPR029058 | -2.16 | 0.95 | Down |
| *POX02646* | NA | NA | GH76 | IPR005198, IPR008928, IPR012341 | 1.72 | 0.90 | Up |
| *POX02682* | Myb | NA | NA | IPR001005, IPR009057, IPR017877, IPR017930 | -1.58 | 0.91 | Down |
| *POX02691* | NA | NA | NA | IPR001395, IPR029058, IPR020471, IPR023210 | -1.76 | 0.86 | Down |
| *POX02693* | NA | NA | NA | IPR000073,, IPR018170 | -2.21 | 0.85 | Down |
| *POX02694* | NA | NA | NA | IPR000627, IPR007535, IPR015889 | -2.40 | 0.87 | Down |
| *POX02709* | NA | NA | NA | NA | 7.74 | 0.88 | Up |
| *POX02710* | NA | NA | GH71 | IPR005197 | -2.93 | 0.93 | Down |
| *POX02721* | NA | NA | NA | IPR000791 | -1.22 | 0.93 | Down |
| *POX02739* | NA | NA | CE16 | IPR001087, IPR013830 | -2.37 | 0.95 | Down |
| *POX02741* | NA | NA | NA | IPR009571 | 1.51 | 0.93 | Up |
| *POX02754* | NA | NA | NA | NA | -1.74 | 0.91 | Down |
| *POX02755* | NA | NA | NA | IPR003579, IPR005225, IPR006689, IPR024156, IPR027417 | -1.06 | 0.89 | Down |
| *POX02756* | NA | NA | NA | IPR021331 | 3.46 | 0.86 | Up |
| *POX02768* | C2H2 zinc finger | NA | NA | IPR007087, IPR013087, IPR015880 | 1.01 | 0.85 | Up |
| *POX02774* | NA | NA | NA | IPR002220, IPR013785 | -1.49 | 0.89 | Down |
| *POX02792* | NA | NA | NA | IPR005593, IPR009014, IPR018969, IPR018970, IPR019789, IPR019790, IPR029061 | -1.02 | 0.82 | Down |

| | | | | | | | |
|---|---|---|---|---|---|---|---|
| *POX02798* | NA | NA | NA | IPR013112, IPR013121, IPR013130, IPR017927, IPR017938 | 2.40 | 0.92 | Up |
| *POX02830* | NA | NA | NA | IPR000873, IPR020845, IPR025110 | -2.15 | 0.86 | Down |
| *POX02831* | NA | NA | NA | IPR002655, IPR009075, IPR009100, IPR012258, IPR013786, IPR029320 | -1.96 | 0.86 | Down |
| *POX02832* | NA | NA | NA | IPR008183, IPR011013, IPR014718 | -1.60 | 0.93 | Down |
| *POX02847* | NA | NA | NA | NA | -2.93 | 0.96 | Down |
| *POX02860* | NA | NA | NA | NA | 9.22 | 1.00 | Up |
| *POX02861* | NA | NA | NA | IPR007603 | 2.10 | 0.92 | Up |
| *POX02867* | NA | NA | NA | IPR011701, IPR020846 | 6.84 | 0.84 | Up |
| *POX02875* | NA | NA | NA | IPR001757, IPR006121, IPR006122, IPR008250, IPR017969, IPR018303, IPR023214, IPR023299, IPR027256 | 3.25 | 0.91 | Up |
| *POX02905* | NA | NA | NA | IPR004045, IPR004046, IPR010987, IPR012336 | -1.73 | 0.92 | Down |
| *POX02928* | NA | NA | NA | IPR000352 | 1.39 | 0.84 | Up |
| *POX02972* | NA | NA | NA | NA | -2.08 | 0.90 | Down |
| *POX02977* | NA | NA | CE9 | IPR002195, IPR006680, IPR011059, IPR017593, IPR032466 | -1.55 | 0.81 | Down |
| *POX02985* | NA | NA | NA | IPR001128, IPR010255, IPR019791 | 1.47 | 0.92 | Up |
| *POX02986* | NA | NA | NA | IPR018712 | 1.63 | 0.90 | Up |
| *POX02999* | NA | NA | NA | NA | -2.83 | 0.96 | Down |
| *POX03005* | NA | NA | CE5 | IPR000675, IPR011150, IPR029058 | -2.23 | 0.94 | Down |
| *POX03020* | NA | NA | NA | NA | 3.34 | 0.93 | Up |
| *POX03021* | NA | NA | GH18 | IPR001223, IPR001579, IPR011583, IPR013781, IPR017853, IPR029070 | 1.82 | 0.94 | Up |
| *POX03025* | NA | NA | GH109 | IPR000683, IPR004104, IPR016040 | -1.24 | 0.91 | Down |
| *POX03042* | NA | NA | NA | NA | 1.02 | 0.87 | Up |
| *POX03044* | NA | NA | NA | IPR002198, IPR002347, IPR016040 | -1.00 | 0.86 | Down |
| *POX03048* | NA | NA | NA | IPR011766, IPR012000, IPR012001, IPR012110, IPR029035, IPR029061 | -1.05 | 0.91 | Down |
| *POX03060* | NA | NA | NA | IPR004877, IPR006593 | 1.42 | 0.94 | Up |
| *POX03062* | NA | β-glucosidase | GH1 | IPR001360, IPR013781, IPR017853 | -1.06 | 0.88 | Down |
| *POX03068* | NA | NA | NA | IPR019371 | -1.81 | 0.92 | Down |
| *POX03085* | NA | NA | NA | IPR006353, IPR006357, IPR023214 | -1.63 | 0.95 | Down |

Journal of Fungi

MDPI

| | | | | | | | |
|---|---|---|---|---|---|---|---|
| *POX03086* | NA | NA | NA | IPR009000, IPR012947, IPR018163, IPR018164 | -2.00 | 0.92 | Down |
| *POX03095* | NA | NA | NA | IPR023753, IPR025700 | -1.75 | 0.95 | Down |
| *POX03104* | NA | NA | NA | IPR011701, IPR020846 | -1.32 | 0.93 | Down |
| *POX03110* | NA | NA | NA | NA | 1.39 | 0.83 | Up |
| *POX03124* | NA | NA | NA | NA | 1.15 | 0.85 | Up |
| *POX03135* | NA | NA | NA | IPR002182, IPR027417, IPR029058 | 1.02 | 0.82 | Up |
| *POX03139* | NA | NA | NA | NA | 1.14 | 0.83 | Up |
| *POX03147* | NA | NA | NA | IPR031352, IPR031353 | -1.27 | 0.89 | Down |
| *POX03148* | NA | NA | NA | IPR031352, IPR031353 | -1.38 | 0.82 | Down |
| *POX03155* | NA | NA | NA | IPR000120, IPR023631 | -1.46 | 0.84 | Down |
| *POX03170* | NA | NA | NA | NA | 1.20 | 0.84 | Up |
| *POX03195* | NA | NA | NA | IPR015157 | -1.03 | 0.90 | Down |
| *POX03202* | NA | NA | NA | IPR000182, IPR016181 | -1.17 | 0.91 | Down |
| *POX03234* | NA | NA | NA | IPR008217 | 1.08 | 0.83 | Up |
| *POX03249* | NA | NA | NA | IPR004843, IPR029052 | 3.69 | 0.93 | Up |
| *POX03250* | NA | NA | NA | NA | 6.63 | 1.00 | Up |
| *POX03255* | NA | NA | NA | IPR005828, IPR020846 | -2.41 | 0.85 | Down |
| *POX03269* | NA | NA | GT62 | IPR005109 | 1.26 | 0.84 | Up |
| *POX03279* | NA | NA | NA | IPR001757, IPR004014, IPR006068, IPR006414, IPR008250, IPR018303, IPR023214, IPR023298, IPR023299 | 1.48 | 0.82 | Up |
| *POX03281* | NA | NA | NA | IPR013112, IPR013121, IPR013130, IPR017927 | 2.29 | 0.83 | Up |
| *POX03284* | NA | NA | NA | IPR002085, IPR011032, IPR013149, IPR013154, IPR016040, IPR020843 | 1.90 | 0.93 | Up |
| *POX03293* | NA | NA | NA | IPR000138, IPR000891, IPR013785 | 1.68 | 0.92 | Up |
| *POX03296* | NA | NA | NA | IPR006694 | 1.28 | 0.80 | Up |
| *POX03297* | NA | NA | NA | IPR008914 | -2.93 | 0.91 | Down |
| *POX03300* | NA | NA | NA | NA | 1.73 | 0.95 | Up |
| *POX03328* | NA | NA | NA | NA | 1.17 | 0.83 | Up |
| *POX03343* | NA | NA | NA | IPR000073, IPR029058 | 1.38 | 0.87 | Up |
| *POX03344* | NA | NA | NA | IPR000639, IPR010497, IPR016292, IPR029058 | 1.21 | 0.91 | Up |
| *POX03345* | NA | NA | NA | NA | -1.83 | 0.87 | Down |
| *POX03347* | NA | NA | NA | IPR004991, IPR023307 | 1.21 | 0.92 | Up |

| | | | | | | | |
|---|---|---|---|---|---|---|---|
| *POX03368* | NA | NA | AA7 | IPR006094, IPR012951, IPR016166, IPR016169 | -1.03 | 0.83 | Down |
| *POX03369* | NA | NA | NA | IPR016040 | -1.51 | 0.87 | Down |
| *POX03379* | NA | NA | NA | IPR004695 | 6.68 | 0.99 | Up |
| *POX03380* | Zn2Cys6 | NA | NA | IPR007219 | 2.76 | 0.93 | Up |
| *POX03381* | NA | NA | NA | IPR010300, IPR011051, IPR014710 | 3.45 | 0.96 | Up |
| *POX03389* | NA | NA | NA | NA | 1.49 | 0.94 | Up |
| *POX03391* | NA | NA | NA | NA | 1.36 | 0.87 | Up |
| *POX03392* | NA | NA | NA | IPR021740 | 1.34 | 0.89 | Up |
| *POX03393* | NA | NA | NA | NA | 1.59 | 0.85 | Up |
| *POX03395* | NA | NA | NA | IPR014870, IPR032514 | 4.32 | 0.94 | Up |
| *POX03398* | NA | NA | NA | IPR006644, IPR013783, IPR015919 | 2.51 | 0.90 | Up |
| *POX03402* | NA | NA | NA | IPR011045, IPR015943, IPR019405 | 1.41 | 0.91 | Up |
| *POX03407* | bZIP | NA | NA | IPR004827 | 4.27 | 0.80 | Up |
| *POX03411* | NA | NA | CE10 | IPR013094, IPR029058 | 1.66 | 0.90 | Up |
| *POX03412* | NA | NA | NA | IPR004235, IPR032710 | 3.00 | 0.97 | Up |
| *POX03413* | NA | NA | NA | IPR002198, IPR002347, IPR013968, IPR016040, IPR020904 | 3.16 | 0.97 | Up |
| *POX03414* | NA | NA | NA | IPR003807 | 2.25 | 0.86 | Up |
| *POX03420* | NA | Exo-α-L-1'5-arabinanase | GH33; GH74; GH93 | IPR011040 | 1.82 | 0.82 | Up |
| *POX03421* | NA | NA | NA | IPR002198, IPR016040 | 3.74 | 0.96 | Up |
| *POX03426* | NA | NA | NA | IPR006838 | 3.03 | 0.95 | Up |
| *POX03429* | NA | NA | NA | NA | 1.21 | 0.88 | Up |
| *POX03430* | NA | Endo-β-1'4-xylanase | GH11 | IPR001137, IPR013319, IPR013320, IPR018208 | 1.57 | 0.95 | Up |
| *POX03434* | NA | NA | NA | NA | -1.25 | 0.83 | Down |
| *POX03436* | NA | NA | NA | NA | -1.25 | 0.82 | Down |
| *POX03453* | NA | NA | NA | IPR000572, IPR001199, IPR005066, IPR008335, IPR014756 | 2.57 | 0.92 | Up |
| *POX03454* | NA | NA | NA | IPR001395, IPR005399, IPR023210 | 1.07 | 0.84 | Up |
| *POX03459* | NA | NA | NA | IPR001425, IPR018229, IPR029730 | 1.47 | 0.90 | Up |

| | | | | | | | |
|---|---|---|---|---|---|---|---|
| *POX03460* | NA | NA | NA | IPR001757, IPR004014, IPR006068, IPR008250, IPR018303, IPR023214, IPR023298, IPR023299 | 4.26 | 0.94 | Up |
| *POX09801* | NA | NA | GH43 | IPR006710, IPR008979, IPR023296 | -1.86 | 0.83 | Down |
| *POX09803* | NA | NA | NA | NA | 11.25 | 0.95 | Up |
| *POX09812* | NA | NA | NA | IPR001680, IPR015943, IPR017986, IPR019775, IPR020472 | -2.01 | 0.86 | Down |
| *POX09819* | NA | NA | NA | IPR027417 | -3.67 | 0.91 | Down |
| *POX09822* | NA | NA | NA | IPR007312 | -1.51 | 0.94 | Down |
| *POX03464* | NA | NA | NA | NA | -1.78 | 0.92 | Down |
| *POX03465* | NA | NA | NA | NA | -1.98 | 0.94 | Down |
| *POX03475* | NA | NA | NA | IPR011990, IPR027417 | 4.36 | 0.95 | Up |
| *POX03483* | NA | NA | NA | IPR000022, IPR000089, IPR001882, IPR005479, IPR005481, IPR005482, IPR011053, IPR011054, IPR011761, IPR011762, IPR011763, IPR011764, IPR013537, IPR013815, IPR013816, IPR016185, IPR029045 | 1.02 | 0.85 | Up |
| *POX03496* | NA | NA | NA | IPR011042 | 4.25 | 0.98 | Up |
| *POX03505* | NA | NA | NA | IPR002933, IPR011650, IPR017439 | -3.76 | 0.95 | Down |
| *POX03511* | NA | NA | NA | IPR012349, IPR024624 | 1.43 | 0.91 | Up |
| *POX03521* | NA | NA | NA | IPR023214 | -1.83 | 0.94 | Down |
| *POX03523* | NA | NA | AA3; AA8 | IPR000172, IPR007867, IPR012132, IPR023753 | -1.22 | 0.83 | Down |
| *POX03532* | NA | NA | NA | IPR002198, IPR002347, IPR016040, IPR020904 | 1.61 | 0.93 | Up |
| *POX03572* | NA | NA | NA | IPR008427, IPR014005 | 3.41 | 0.97 | Up |
| *POX03603* | NA | NA | NA | PR002918, IPR029058 | 1.58 | 0.93 | Up |
| *POX03606* | NA | NA | NA | NA | 2.96 | 0.94 | Up |
| *POX03617* | NA | NA | NA | IPR011701, IPR020846 | -1.00 | 0.87 | Down |
| *POX03625* | NA | NA | NA | IPR002293 | -1.07 | 0.86 | Down |
| *POX03632* | NA | NA | NA | NA | 1.89 | 0.95 | Up |
| *POX03638* | NA | NA | NA | IPR021487 | 2.35 | 0.90 | Up |
| *POX03641* | NA | β-glucosidase | GH3 | IPR001764, IPR002772, IPR011658, IPR017853, IPR019800, IPR026891, IPR026892 | -2.09 | 0.95 | Down |

| | | | | | | | |
|---|---|---|---|---|---|---|---|
| *POX03645* | NA | NA | NA | IPR013057 | 5.12 | 0.88 | Up |
| *POX03669* | NA | NA | NA | IPR021858 | 4.86 | 0.92 | Up |
| *POX03675* | NA | NA | NA | IPR008146, IPR008147, IPR014746 | 1.53 | 0.89 | Up |
| *POX03676* | NA | NA | NA | IPR002198, IPR002347, IPR016040 | 1.19 | 0.90 | Up |
| *POX03711* | NA | Acetyl | CE5 | IPR000675, IPR029058 | -1.38 | 0.93 | Down |
| *POX03741* | NA | NA | GH13 | IPR006047, IPR006589, IPR013780, IPR013781, IPR015902, IPR017853, IPR022567 | -1.28 | 0.84 | Down |
| *POX03742* | NA | NA | NA | IPR003663, IPR005828, IPR005829, IPR020846 | -2.03 | 0.91 | Down |
| *POX03743* | NA | NA | NA | NA | 10.37 | 1.00 | Up |
| *POX03744* | NA | NA | NA | NA | 10.27 | 0.99 | Up |
| *POX03777* | NA | NA | NA | IPR000277, IPR015421, IPR015422, IPR015424 | 1.86 | 0.89 | Up |
| *POX03782* | NA | NA | NA | IPR006771 | -2.79 | 0.97 | Down |
| *POX03798* | NA | NA | NA | NA | 5.80 | 0.87 | Up |
| *POX03804* | NA | NA | NA | IPR006328, IPR006439, IPR023214 | -1.52 | 0.89 | Down |
| *POX03806* | NA | NA | NA | IPR001199, IPR018506 | 1.04 | 0.91 | Up |
| *POX03808* | NA | NA | NA | NA | 1.06 | 0.87 | Up |
| *POX03814* | NA | NA | NA | NA | 1.14 | 0.82 | Up |
| *POX03815* | NA | NA | NA | NA | 1.13 | 0.82 | Up |
| *POX03820* | NA | NA | NA | NA | -1.58 | 0.82 | Down |
| *POX03828* | NA | NA | NA | IPR001683, IPR003114, IPR013937, IPR013996 | 1.12 | 0.83 | Up |
| *POX03829* | NA | NA | NA | IPR003736, IPR006683, IPR029069 | 1.26 | 0.80 | Up |
| *POX03833* | NA | NA | NA | IPR004299, IPR014371 | 1.40 | 0.92 | Up |
| *POX03844* | NA | NA | NA | IPR000183, IPR002433, IPR009006, IPR022643, IPR022644, IPR022653 | 2.23 | 0.93 | Up |
| *POX03849* | NA | NA | NA | NA | 6.28 | 0.98 | Up |
| *POX03857* | NA | NA | NA | NA | -1.33 | 0.87 | Down |
| *POX03860* | NA | NA | NA | NA | -2.20 | 0.89 | Down |
| *POX03862* | NA | NA | NA | IPR008030, IPR016040 | -1.18 | 0.91 | Down |
| *POX03863* | NA | NA | NA | IPR002085, IPR011032, IPR016040, IPR020843 | -1.56 | 0.90 | Down |
| *POX03871* | NA | NA | NA | IPR011598 | -2.50 | 0.93 | Down |

| | | | | | | | |
|---|---|---|---|---|---|---|---|
| *POX03873* | Zn2Cys6 | NA | NA | IPR001138 | -2.86 | 0.94 | Down |
| *POX03889* | NA | NA | GH31 | IPR000322, IPR011013, IPR017853, IPR030458, IPR030459, IPR031727 | 1.25 | 0.85 | Up |
| *POX03898* | NA | NA | NA | IPR001128, IPR002403, IPR017972 | 6.00 | 0.96 | Up |
| *POX03902* | NA | NA | NA | NA | 4.86 | 0.87 | Up |
| *POX03912* | NA | NA | NA | IPR005814, IPR015421, IPR015422, IPR015424 | -2.14 | 0.90 | Down |
| *POX03927* | NA | NA | NA | IPR003480, IPR023213 | -1.92 | 0.83 | Down |
| *POX03929* | NA | NA | NA | IPR027417 | -1.92 | 0.93 | Down |
| *POX03930* | NA | NA | NA | IPR007568 | -2.42 | 0.95 | Down |
| *POX03931* | NA | NA | NA | IPR000542, IPR001227, IPR009081, IPR011032, IPR013217, IPR013968, IPR014030, IPR014031, IPR014043, IPR016035, IPR016036, IPR016039, IPR016040, IPR020801, IPR020806, IPR020807, IPR020841, IPR020843, IPR029063, IPR032821 | -2.84 | 0.80 | Down |
| *POX03946* | NA | NA | NA | NA | 3.85 | 0.88 | Up |
| *POX03952* | NA | NA | NA | IPR018392 | 7.21 | 0.97 | Up |
| *POX03956* | NA | NA | NA | IPR000269, IPR015798, IPR015800, IPR015802, IPR016182 | 2.08 | 0.83 | Up |
| *POX03960* | NA | NA | NA | IPR004843, IPR006186, IPR029052, IPR031675 | 2.60 | 0.88 | Up |
| *POX03973* | NA | NA | NA | IPR021838 | -1.54 | 0.85 | Down |
| *POX03976* | NA | NA | NA | NA | 5.45 | 0.99 | Up |
| *POX04020* | NA | NA | NA | NA | 6.38 | 0.92 | Up |
| *POX04025* | NA | NA | NA | NA | -2.16 | 0.81 | Down |
| *POX04036* | NA | NA | NA | IPR009571 | 2.49 | 0.94 | Up |
| *POX04050* | NA | NA | NA | IPR001544, IPR005786, IPR018300 | 1.71 | 0.91 | Up |
| *POX04067* | NA | NA | NA | NA | 1.83 | 0.87 | Up |
| *POX04087* | NA | NA | NA | NA | 1.55 | 0.92 | Up |
| *POX04103* | NA | NA | NA | IPR025509 | 2.36 | 0.91 | Up |
| *POX04104* | NA | NA | NA | IPR002524, IPR027469 | 3.53 | 0.88 | Up |
| *POX04109* | NA | NA | NA | IPR006652, IPR011043, IPR015915 | 1.41 | 0.92 | Up |

| | | | | | | | |
|---|---|---|---|---|---|---|---|
| *POX04132* | NA | NA | NA | IPR002048, IPR006685, IPR010920, IPR011992, IPR018247 | 1.46 | 0.87 | Up |
| *POX04135* | NA | NA | NA | IPR012816 | 1.22 | 0.87 | Up |
| *POX04137* | NA | Endo-β-1'4-glucanase | CBM1; GH5 | IPR000254, IPR001547, IPR013781, IPR017853 | -1.87 | 0.90 | Down |
| *POX04147* | NA | NA | NA | IPR001466, IPR012338 | 5.44 | 0.92 | Up |
| *POX04193* | Zn2Cys6 | NA | NA | IPR007219 | 2.34 | 0.87 | Up |
| *POX04195* | NA | NA | NA | NA | -1.42 | 0.92 | Down |
| *POX04197* | NA | NA | NA | IPR008862 | 1.74 | 0.81 | Up |
| *POX04214* | NA | NA | NA | IPR002376, IPR023753 | 1.96 | 0.89 | Up |
| *POX04215* | NA | NA | NA | IPR013901 | 2.90 | 0.97 | Up |
| *POX04224* | NA | NA | NA | IPR001128, IPR002403, IPR017972 | 4.43 | 0.94 | Up |
| *POX04225* | NA | NA | NA | IPR004839, IPR015421, IPR015422, IPR015424 | 3.22 | 0.85 | Up |
| *POX04226* | NA | NA | NA | IPR002938, IPR023753 | 3.55 | 0.87 | Up |
| *POX04227* | Winged helix repressor DNA-binding | NA | NA | IPR001077, IPR011991, IPR016461, IPR029063 | 3.56 | 0.89 | Up |
| *POX04230* | NA | NA | NA | IPR001461, IPR001969, IPR021109 | -1.10 | 0.87 | Down |
| *POX04240* | NA | NA | NA | IPR032710 | 2.46 | 0.89 | Up |
| *POX04244* | NA | NA | GT71 | IPR022751, IPR029044 | 3.03 | 0.91 | Up |
| *POX04258* | NA | NA | NA | IPR001905, IPR018047, IPR024041, IPR029020 | -1.64 | 0.87 | Down |
| *POX04274* | NA | Endo-β-1'4-xylanase | GH30 | IPR001139, IPR013780, IPR013781, IPR017853 | -2.04 | 0.89 | Down |
| *POX04276* | NA | NA | NA | IPR003663, IPR005828, IPR005829, IPR020846 | -2.49 | 0.95 | Down |
| *POX04278* | NA | NA | NA | NA | -1.48 | 0.93 | Down |
| *POX04280* | NA | NA | NA | IPR001313, IPR011989, IPR016024 | 1.12 | 0.85 | Up |
| *POX04338* | NA | NA | NA | NA | 1.01 | 0.85 | Up |
| *POX04344* | NA | NA | NA | IPR002293 | -2.23 | 0.93 | Down |
| *POX04345* | NA | NA | NA | NA | -2.21 | 0.94 | Down |
| *POX04350* | NA | NA | NA | IPR011701, IPR020846 | 4.30 | 0.91 | Up |
| *POX04352* | NA | NA | NA | IPR028116 | 1.74 | 0.87 | Up |

| | | | | | | | |
|---|---|---|---|---|---|---|---|
| *POX04356* | NA | NA | NA | IPR002020, IPR016142, IPR019810 | 1.08 | 0.89 | Up |
| *POX04360* | NA | NA | NA | IPR001465, IPR006252, IPR011076, IPR019830 | 1.81 | 0.92 | Up |
| *POX04369* | NA | NA | NA | IPR003663, IPR005828, IPR005829, IPR020846 | 3.23 | 0.87 | Up |
| *POX04373* | NA | NA | NA | IPR001279 | -2.71 | 0.91 | Down |
| *POX04387* | NA | NA | NA | IPR001227, IPR006162, IPR009081, IPR011032, IPR013149, IPR013217, IPR013968, IPR014030, IPR014031, IPR014043, IPR016035, IPR016036, IPR016039, IPR016040, IPR018201, IPR020801, IPR020806, IPR020807, IPR020841, IPR020843, IPR029063, IPR032821 | 1.73 | 0.85 | Up |
| *POX04388* | NA | NA | NA | IPR005645, IPR029058 | 1.78 | 0.94 | Up |
| *POX04389* | NA | NA | NA | IPR011701, IPR020846 | 2.95 | 0.95 | Up |
| *POX04390* | NA | NA | CBM43; GH72 | IPR004886, IPR012946, IPR013781, IPR017853 | -1.46 | 0.94 | Down |
| *POX04391* | NA | NA | NA | IPR006254, IPR015813, IPR018523 | 1.10 | 0.86 | Up |
| *POX04410* | NA | NA | NA | NA | 9.24 | 0.99 | Up |
| *POX04411* | NA | NA | NA | NA | 2.74 | 0.89 | Up |
| *POX04420* | C2H2 zinc finger | NA | NA | IPR007087, IPR013087, IPR015880 | -1.91 | 0.94 | Down |
| *POX04456* | NA | NA | NA | IPR005151, IPR029045 | 1.36 | 0.82 | Up |
| *POX04463* | NA | NA | NA | IPR000719, IPR002290, IPR008271, IPR011009, IPR017441, IPR031850 | 7.19 | 0.97 | Up |
| *POX04467* | NA | NA | NA | IPR012808 | 1.23 | 0.82 | Up |
| *POX04478* | NA | NA | NA | IPR011701, IPR020846 | 1.47 | 0.88 | Up |
| *POX04480* | NA | NA | NA | IPR001128, IPR002401, IPR017972 | 2.97 | 0.92 | Up |
| *POX04515* | NA | NA | NA | NA | -1.24 | 0.91 | Down |
| *POX04519* | NA | NA | NA | NA | -1.11 | 0.88 | Down |
| *POX04520* | C2H2 zinc finger | NA | NA | IPR007087, IPR013087, IPR015880 | -1.11 | 0.89 | Down |
| *POX04522* | NA | NA | NA | IPR023753 | -1.14 | 0.89 | Down |
| *POX04523* | NA | NA | NA | NA | -1.29 | 0.88 | Down |
| *POX04524* | NA | NA | NA | NA | -3.10 | 0.97 | Down |

Journal of Fungi

| | | | | | | | |
|---|---|---|---|---|---|---|---|
| *POX04529* | NA | NA | NA | IPR003663, IPR005828, IPR005829, IPR020846 | 1.68 | 0.87 | Up |
| *POX04530* | NA | NA | NA | IPR000182, IPR016181 | 2.29 | 0.88 | Up |
| *POX04533* | NA | NA | NA | IPR003663, IPR005828, IPR005829, IPR020846 | 2.03 | 0.88 | Up |
| *POX04536* | NA | NA | CBM24; GH71 | IPR005197 | 2.02 | 0.91 | Up |
| *POX09829* | NA | NA | NA | NA | -2.63 | 0.92 | Down |
| *POX04559* | NA | NA | NA | IPR003378, IPR003609 | 2.06 | 0.93 | Up |
| *POX04560* | NA | NA | NA | IPR011701, IPR020846 | 1.62 | 0.90 | Up |
| *POX04561* | NA | NA | NA | IPR001077, IPR016461, IPR029063 | 2.39 | 0.94 | Up |
| *POX04562* | NA | NA | NA | NA | 3.09 | 0.95 | Up |
| *POX04564* | NA | NA | NA | IPR011701, IPR020846 | -2.29 | 0.93 | Down |
| *POX04565* | NA | NA | NA | NA | -2.89 | 0.92 | Down |
| *POX04566* | NA | NA | NA | IPR023753 | -1.48 | 0.83 | Down |
| *POX04568* | NA | NA | NA | IPR006153 | 3.59 | 0.94 | Up |
| *POX04569* | NA | NA | NA | IPR001129, IPR023352 | 3.99 | 0.95 | Up |
| *POX04573* | NA | NA | NA | IPR001128, IPR002403 | 1.14 | 0.87 | Up |
| *POX04574* | NA | NA | NA | IPR002198, IPR002347, IPR016040, IPR020904 | 1.78 | 0.92 | Up |
| *POX04577* | NA | NA | NA | NA | 4.35 | 0.94 | Up |
| *POX04589* | NA | NA | NA | NA | 4.99 | 0.98 | Up |
| *POX04590* | Zn2Cys6 | NA | NA | IPR001138, IPR007219 | 1.26 | 0.89 | Up |
| *POX04592* | NA | NA | NA | IPR001395, IPR018170, IPR020471, IPR023210 | 5.24 | 0.95 | Up |
| *POX04606* | NA | NA | NA | IPR013057 | -2.69 | 0.96 | Down |
| *POX04628* | NA | NA | NA | NA | -1.33 | 0.90 | Down |
| *POX04631* | NA | NA | NA | IPR029062 | 6.06 | 0.95 | Up |
| *POX04653* | NA | NA | NA | NA | 2.64 | 0.83 | Up |
| *POX04681* | NA | NA | NA | IPR011701, IPR020846 | 1.43 | 0.83 | Up |
| *POX04686* | NA | NA | GH128 | IPR017853, IPR024655 | -2.57 | 0.96 | Down |
| *POX04693* | NA | NA | NA | NA | 8.51 | 1.00 | Up |
| *POX04700* | NA | NA | NA | IPR001789, IPR003594, IPR003661, IPR004358, IPR005467, IPR011006, IPR029016 | -1.14 | 0.87 | Down |
| *POX04714* | NA | NA | NA | IPR002198, IPR002347, IPR016040 | 2.81 | 0.92 | Up |
| *POX04725* | NA | NA | NA | IPR011598 | 1.38 | 0.92 | Up |

| | | | | | | | |
|---|---|---|---|---|---|---|---|
| *POX04726* | NA | NA | NA | NA | 2.91 | 0.97 | Up |
| *POX04764* | NA | NA | NA | NA | -1.18 | 0.89 | Down |
| *POX04769* | C2H2 zinc finger | NA | NA | IPR007087, IPR013087, IPR015880 | -1.18 | 0.90 | Down |
| *POX04779* | NA | NA | NA | IPR001248, IPR026030 | -1.74 | 0.82 | Down |
| *POX04786* | NA | Cellobiohydrolase | CBM1; GH6 | IPR000254, IPR001524, IPR016288 | -1.40 | 0.94 | Down |
| *POX04795* | C2H2 zinc finger | NA | NA | IPR007087, IPR013087, IPR015880 | -1.69 | 0.89 | Down |
| *POX04800* | NA | NA | NA | NA | -2.54 | 0.93 | Down |
| *POX04809* | NA | NA | NA | NA | 5.63 | 0.99 | Up |
| *POX04837* | NA | NA | NA | IPR013057 | 4.38 | 0.87 | Up |
| *POX04838* | NA | NA | NA | IPR001753, IPR029045 | 1.51 | 0.87 | Up |
| *POX04856* | NA | NA | NA | NA | 1.64 | 0.82 | Up |
| *POX04870* | NA | NA | NA | IPR013933 | 1.09 | 0.84 | Up |
| *POX04894* | NA | NA | NA | IPR013948 | 1.06 | 0.80 | Up |
| *POX04895* | NA | NA | GH92 | IPR005887, IPR008928, IPR012939, IPR014718 | 1.96 | 0.93 | Up |
| *POX04907* | NA | NA | NA | IPR013022 | 4.39 | 0.82 | Up |
| *POX04914* | NA | NA | NA | NA | 4.80 | 0.83 | Up |
| *POX04916* | NA | NA | NA | IPR011701, IPR020846 | 6.90 | 0.97 | Up |
| *POX04927* | NA | NA | NA | NA | 6.05 | 0.98 | Up |
| *POX04952* | NA | NA | NA | NA | 4.88 | 0.96 | Up |
| *POX05007* | NA | NA | NA | IPR001461, IPR001969, IPR021109 | -3.32 | 0.96 | Down |
| *POX05015* | NA | NA | NA | IPR001185 | -1.90 | 0.81 | Down |
| *POX05016* | NA | NA | NA | NA | -1.98 | 0.93 | Down |
| *POX05017* | NA | NA | NA | IPR006461 | -1.76 | 0.94 | Down |
| *POX05046* | NA | NA | NA | IPR029032 | 1.19 | 0.90 | Up |
| *POX05055* | NA | NA | NA | IPR000917, IPR017849, IPR017850, IPR024607 | -1.03 | 0.87 | Down |
| *POX05062* | NA | NA | NA | NA | -1.42 | 0.92 | Down |
| *POX05081* | NA | NA | NA | NA | -1.48 | 0.94 | Down |
| *POX05089* | NA | NA | NA | NA | 3.07 | 0.86 | Up |
| *POX05090* | NA | NA | NA | NA | 1.68 | 0.91 | Up |
| *POX05092* | NA | NA | NA | IPR000873, IPR025110 | 3.11 | 0.95 | Up |
| *POX05095* | NA | NA | NA | NA | 3.87 | 0.95 | Up |

| | | | | | | | |
|---|---|---|---|---|---|---|---|
| *POX05111* | NA | NA | NA | NA | -1.18 | 0.88 | Down |
| *POX05128* | NA | NA | NA | IPR018020 | 1.21 | 0.91 | Up |
| *POX05129* | NA | NA | NA | IPR001680, IPR015943, IPR017986 | 1.70 | 0.90 | Up |
| *POX05132* | NA | NA | CE5 | IPR000675, IPR011150, IPR029058 | 2.53 | 0.96 | Up |
| *POX05139* | NA | NA | NA | IPR000620, IPR025016 | 1.68 | 0.90 | Up |
| *POX05190* | C2H2 zinc finger | NA | NA | IPR007087, IPR013087, IPR015880 | -2.45 | 0.91 | Down |
| *POX05218* | NA | NA | NA | NA | -1.23 | 0.80 | Down |
| *POX05220* | NA | NA | NA | NA | -3.14 | 0.87 | Down |
| *POX05224* | NA | NA | NA | IPR008493, IPR031318 | 1.15 | 0.87 | Up |
| *POX05232* | NA | NA | NA | IPR005123, IPR026992, IPR027443 | -1.36 | 0.86 | Down |
| *POX05233* | NA | NA | NA | NA | -1.45 | 0.90 | Down |
| *POX05237* | NA | NA | NA | NA | -1.66 | 0.89 | Down |
| *POX05248* | NA | NA | NA | IPR006076, IPR006181, IPR016040, IPR023209 | -1.34 | 0.87 | Down |
| *POX05256* | NA | NA | NA | IPR000407 | -1.65 | 0.92 | Down |
| *POX05299* | NA | NA | NA | IPR001806, IPR003578, IPR003579, IPR005225, IPR020849, IPR027417 | 1.13 | 0.85 | Up |
| *POX05301* | NA | NA | NA | NA | -1.89 | 0.95 | Down |
| *POX05306* | NA | NA | NA | NA | -6.44 | 0.99 | Down |
| *POX05307* | NA | NA | NA | NA | -2.12 | 0.93 | Down |
| *POX05320* | NA | NA | NA | IPR002198, IPR002347, IPR016040, IPR020904 | -1.51 | 0.88 | Down |
| *POX05322* | NA | NA | NA | IPR001466, IPR009090, IPR012338, IPR012856, IPR027279 | -1.31 | 0.85 | Down |
| *POX05335* | NA | NA | NA | NA | 2.16 | 0.96 | Up |
| *POX05336* | NA | NA | NA | NA | 2.01 | 0.93 | Up |
| *POX05343* | NA | NA | NA | IPR011701, IPR020846 | 1.39 | 0.86 | Up |
| *POX05344* | NA | NA | NA | IPR002198, IPR002347, IPR016040, IPR020904 | 4.31 | 0.90 | Up |
| *POX05356* | NA | NA | NA | NA | 3.79 | 0.95 | Up |
| *POX05357* | NA | NA | NA | IPR010816 | 1.34 | 0.86 | Up |
| *POX05370* | NA | NA | NA | IPR000182, IPR016181 | 3.01 | 0.93 | Up |
| *POX05372* | NA | NA | GT62 | IPR005109, IPR029044 | 1.58 | 0.88 | Up |
| *POX05378* | NA | NA | NA | NA | -2.08 | 0.96 | Down |
| *POX05384* | NA | NA | NA | NA | 1.62 | 0.85 | Up |

| | | | | | | | |
|---|---|---|---|---|---|---|---|
| *POX05391* | NA | NA | NA | IPR002669 | 1.01 | 0.80 | Up |
| *POX05399* | NA | NA | NA | IPR004367, IPR006671, IPR013763 | 1.31 | 0.88 | Up |
| *POX05420* | NA | NA | NA | IPR009449 | 1.29 | 0.84 | Up |
| *POX05436* | Lambda repressor-like, DNA-binding | NA | NA | IPR003712, IPR008076, IPR010982 | -1.47 | 0.94 | Down |
| *POX05482* | NA | NA | NA | IPR000454, IPR002379, IPR020537 | 1.67 | 0.95 | Up |
| *POX05483* | NA | NA | NA | IPR009784 | 1.93 | 0.87 | Up |
| *POX05492* | NA | NA | NA | IPR002067, IPR018108, IPR023395 | 1.76 | 0.89 | Up |
| *POX05510* | NA | NA | NA | NA | -1.92 | 0.82 | Down |
| *POX05511* | NA | NA | NA | IPR025870 | -2.19 | 0.89 | Down |
| *POX05512* | NA | NA | NA | NA | -2.79 | 0.97 | Down |
| *POX05537* | NA | NA | CBM50 | IPR018392 | 6.76 | 0.98 | Up |
| *POX05540* | NA | β-xylosidase | GH62 | IPR005193, IPR023296 | -1.21 | 0.80 | Down |
| *POX05569* | NA | NA | NA | IPR001461, IPR001969, IPR021109 | 5.39 | 0.97 | Up |
| *POX05570* | NA | Endo-β-1'4-glucanase | CBM1; GH45 | IPR000254, IPR000334, IPR007112, IPR009009 | -1.59 | 0.95 | Down |
| *POX05575* | NA | NA | NA | IPR000250, IPR013320 | 1.09 | 0.91 | Up |
| *POX05578* | NA | NA | NA | IPR001384, IPR024079 | 1.63 | 0.91 | Up |
| *POX05580* | NA | Polygalacturonase; | GH28 | IPR000743, IPR006626, IPR011050, IPR012334 | -2.26 | 0.91 | Down |
| *POX05581* | NA | NA | NA | IPR023753 | 2.35 | 0.92 | Up |
| *POX05585* | NA | NA | NA | IPR011250, IPR020915 | 1.65 | 0.83 | Up |
| *POX05599* | NA | NA | NA | IPR015590, IPR016160, IPR016161, IPR016162, IPR016163, IPR029510 | -1.13 | 0.92 | Down |
| *POX05636* | NA | NA | NA | IPR006115, IPR008927, IPR013328, IPR015815, IPR016040, IPR029154 | 1.02 | 0.82 | Up |
| *POX05645* | NA | NA | AA11 | NA | 7.33 | 0.99 | Up |
| *POX05654* | NA | NA | NA | IPR021460 | 1.04 | 0.87 | Up |
| *POX05655* | NA | NA | NA | NA | 1.16 | 0.84 | Up |

| | | | | | | | |
|---|---|---|---|---|---|---|---|
| *POX05692* | p53-like transcription factor | NA | NA | IPR008967, IPR024061 | 1.46 | 0.92 | Up |
| *POX05693* | NA | NA | NA | NA | 2.32 | 0.88 | Up |
| *POX05696* | NA | NA | NA | IPR004547, IPR006148, IPR018321 | 2.37 | 0.94 | Up |
| *POX05700* | NA | NA | CE9 | IPR003764, IPR006680, IPR011059, IPR032466 | 1.11 | 0.87 | Up |
| *POX05701* | NA | NA | NA | IPR011701, IPR020846 | 1.54 | 0.86 | Up |
| *POX05703* | NA | NA | NA | IPR018306 | -5.33 | 0.96 | Down |
| *POX05717* | NA | NA | NA | IPR027417 | 1.40 | 0.91 | Up |
| *POX05726* | C2H2 zinc finger | NA | NA | IPR007087, IPR013087, IPR015880 | 2.85 | 0.97 | Up |
| *POX05728* | NA | NA | NA | NA | -1.17 | 0.90 | Down |
| *POX05732* | NA | NA | NA | IPR001171, IPR018083 | -2.07 | 0.92 | Down |
| *POX05735* | NA | NA | NA | IPR011701, IPR020846 | 1.44 | 0.83 | Up |
| *POX05736* | NA | NA | NA | NA | 2.69 | 0.89 | Up |
| *POX05737* | NA | NA | NA | IPR000277, IPR015421, IPR015422, IPR015424 | 1.35 | 0.88 | Up |
| *POX05740* | NA | NA | NA | IPR018466 | -1.60 | 0.95 | Down |
| *POX05741* | NA | NA | NA | NA | 1.71 | 0.92 | Up |
| *POX05742* | NA | NA | NA | IPR023753 | 4.64 | 0.97 | Up |
| *POX05756* | NA | NA | NA | IPR001106, IPR005922, IPR008948, IPR022313, IPR024083 | 5.53 | 0.89 | Up |
| *POX05767* | NA | NA | NA | NA | -1.58 | 0.95 | Down |
| *POX05776* | NA | NA | NA | IPR006683, IPR029069 | 2.51 | 0.92 | Up |
| *POX05787* | NA | NA | NA | IPR001312, IPR022672, IPR022673 | 1.58 | 0.89 | Up |
| *POX05823* | NA | NA | NA | IPR001395, IPR023210 | -1.31 | 0.91 | Down |
| *POX05830* | NA | NA | NA | NA | -1.04 | 0.83 | Down |
| *POX05841* | NA | NA | NA | IPR001330, IPR008930, IPR026873 | 1.21 | 0.84 | Up |
| *POX05850* | NA | NA | NA | IPR001128, IPR002401, IPR017972 | 1.79 | 0.93 | Up |
| *POX05851* | NA | NA | NA | IPR000873, IPR001242, IPR006162, IPR009081, IPR013120, IPR016040, IPR020806, IPR020845 | 1.86 | 0.83 | Up |
| *POX05852* | NA | NA | AA7 | IPR006094, IPR012951, IPR016166, IPR016169 | 2.34 | 0.94 | Up |

| | | | | | | | |
|---|---|---|---|---|---|---|---|
| *POX05853* | NA | NA | NA | IPR001128, IPR002401, IPR017972 | 1.28 | 0.89 | Up |
| *POX05855* | NA | NA | NA | IPR008775 | 2.60 | 0.97 | Up |
| *POX05856* | NA | NA | NA | IPR029063 | 1.62 | 0.93 | Up |
| *POX05857* | NA | NA | NA | IPR008030, IPR016040 | 1.48 | 0.90 | Up |
| *POX05858* | NA | NA | NA | IPR008775 | 1.02 | 0.89 | Up |
| *POX05860* | NA | NA | NA | IPR008030, IPR016040 | 1.96 | 0.87 | Up |
| *POX05861* | NA | NA | NA | IPR002938, IPR016040, IPR023753 | 1.70 | 0.90 | Up |
| *POX05864* | NA | NA | NA | IPR008030, IPR016040 | 1.80 | 0.92 | Up |
| *POX05865* | NA | NA | NA | IPR023213 | 1.88 | 0.92 | Up |
| *POX05866* | NA | NA | NA | IPR008775 | 2.51 | 0.96 | Up |
| *POX05868* | NA | NA | NA | IPR008775 | 2.16 | 0.95 | Up |
| *POX05869* | NA | NA | NA | IPR000192, IPR015421, IPR015424 | 1.59 | 0.91 | Up |
| *POX05870* | NA | NA | NA | IPR029063 | 1.53 | 0.93 | Up |
| *POX05874* | NA | NA | NA | IPR000719, IPR002290, IPR011009 | 2.89 | 0.96 | Up |
| *POX05881* | NA | NA | NA | NA | 1.12 | 0.87 | Up |
| *POX05887* | NA | NA | NA | NA | 2.43 | 0.96 | Up |
| *POX05888* | NA | NA | NA | IPR001128, IPR002401 | 2.43 | 0.95 | Up |
| *POX05889* | Homeodomain-like | NA | NA | IPR009057, IPR017877 | 1.05 | 0.82 | Up |
| *POX05892* | NA | NA | NA | NA | 2.38 | 0.93 | Up |
| *POX05898* | NA | NA | NA | NA | 1.97 | 0.95 | Up |
| *POX05902* | NA | NA | NA | IPR000873, IPR005914, IPR020845, IPR025110, IPR032387 | 2.55 | 0.88 | Up |
| *POX05915* | NA | NA | NA | IPR003663, IPR005828, IPR005829, IPR020846 | -2.20 | 0.96 | Down |
| *POX05916* | NA | Endo-β-1'4-xylanase | GH10 | IPR001000, IPR013781, IPR017853 | -2.37 | 0.95 | Down |
| *POX05929* | NA | NA | NA | NA | -2.19 | 0.90 | Down |
| *POX05947* | NA | NA | NA | NA | 3.71 | 0.88 | Up |
| *POX05957* | NA | NA | NA | IPR002938, IPR023753 | -1.57 | 0.84 | Down |
| *POX05962* | NA | NA | NA | IPR007074, IPR009644 | 1.15 | 0.83 | Up |
| *POX05980* | NA | NA | NA | IPR003439, IPR003593, IPR010929, IPR013525, IPR017871, IPR027417, IPR029481 | 6.03 | 0.89 | Up |
| *POX05990* | NA | NA | NA | IPR001248, IPR030175 | 2.39 | 0.91 | Up |

| | | | | | | | |
|---|---|---|---|---|---|---|---|
| *POX05996* | NA | NA | NA | NA | 1.15 | 0.89 | Up |
| *POX05998* | NA | NA | NA | NA | 1.76 | 0.87 | Up |
| *POX06002* | NA | NA | NA | IPR001283, IPR014044, IPR018244 | -1.78 | 0.94 | Down |
| *POX06006* | NA | NA | NA | IPR007325, IPR018247 | -1.16 | 0.83 | Down |
| *POX06025* | NA | NA | NA | NA | 1.29 | 0.89 | Up |
| *POX06029* | NA | NA | NA | IPR006694 | 1.78 | 0.89 | Up |
| *POX06030* | NA | NA | NA | IPR002198, IPR002347, IPR016040, IPR020904 | 1.23 | 0.86 | Up |
| *POX06051* | NA | NA | NA | IPR003663, IPR005828, IPR005829, IPR020846 | -1.89 | 0.96 | Down |
| *POX06054* | NA | NA | NA | NA | 2.64 | 0.91 | Up |
| *POX06055* | NA | NA | NA | IPR004045, IPR004046, IPR010987, IPR012336 | 5.49 | 0.87 | Up |
| *POX06057* | NA | NA | NA | IPR017981, IPR022596, IPR023041 | -1.45 | 0.81 | Down |
| *POX06069* | NA | NA | NA | IPR014898 | 1.02 | 0.84 | Up |
| *POX06079* | NA | β-glucosidase | GH1 | IPR001360, IPR013781, IPR017853, IPR018120 | -2.09 | 0.96 | Down |
| *POX06086* | NA | NA | NA | NA | 2.03 | 0.92 | Up |
| *POX06098* | NA | NA | NA | IPR001128, IPR002401, IPR017972 | 3.98 | 0.89 | Up |
| *POX06102* | NA | NA | NA | NA | 2.18 | 0.96 | Up |
| *POX06117* | NA | NA | NA | IPR006089, IPR006091, IPR009075, IPR009100, IPR013786 | 1.54 | 0.92 | Up |
| *POX06121* | NA | NA | NA | NA | 1.17 | 0.93 | Up |
| *POX06147* | NA | Endo-β-1'4-glucanase | CBM1; GH5 | IPR000254, IPR001547, IPR013781, IPR017853, IPR018087 | -1.15 | 0.92 | Down |
| *POX06151* | NA | NA | NA | NA | 3.09 | 0.94 | Up |
| *POX06153* | NA | NA | NA | IPR007312 | 1.33 | 0.92 | Up |
| *POX06168* | NA | NA | NA | NA | -1.23 | 0.87 | Down |
| *POX06177* | NA | NA | NA | IPR002085, IPR011032, IPR013149, IPR016040, IPR020843 | 1.11 | 0.90 | Up |
| *POX06178* | NA | NA | NA | IPR000103, IPR023753 | 3.04 | 0.87 | Up |
| *POX06207* | NA | NA | NA | IPR021848 | -2.66 | 0.85 | Down |
| *POX06218* | NA | NA | NA | IPR001544, IPR027417 | -1.20 | 0.87 | Down |
| *POX06231* | NA | NA | NA | NA | -2.79 | 0.97 | Down |
| *POX06287* | NA | NA | NA | NA | 11.80 | 0.99 | Up |

| | | | | | | | |
|---|---|---|---|---|---|---|---|
| *POX06301* | NA | NA | GH47 | IPR001382 | 1.49 | 0.93 | Up |
| *POX06311* | NA | NA | NA | IPR023753 | 2.64 | 0.89 | Up |
| *POX06317* | NA | NA | NA | IPR010440, IPR011009 | -4.61 | 0.84 | Down |
| *POX06324* | NA | NA | GH55 | IPR011050, IPR012334, IPR024429, IPR024535 | 4.06 | 0.95 | Up |
| *POX06334* | NA | NA | NA | IPR013112, IPR013121, IPR013130, IPR017927 | 2.33 | 0.87 | Up |
| *POX06337* | NA | NA | NA | IPR003439, IPR003593, IPR005285, IPR010929, IPR013525, IPR017871, IPR027417, IPR029481 | -1.78 | 0.81 | Down |
| *POX06345* | NA | NA | NA | IPR021706, IPR021851 | -2.54 | 0.95 | Down |
| *POX06372* | NA | NA | NA | NA | 3.93 | 0.89 | Up |
| *POX06373* | NA | NA | NA | IPR027417 | 2.78 | 0.85 | Up |
| *POX06377* | Negative transcription al regulator | NA | NA | IPR007396, IPR012349 | -1.43 | 0.91 | Down |
| *POX06380* | NA | NA | GH132 | IPR005556 | -2.05 | 0.87 | Down |
| *POX06395* | NA | NA | NA | IPR001810 | -1.54 | 0.93 | Down |
| *POX06398* | NA | NA | NA | NA | -3.17 | 0.93 | Down |
| *POX06399* | NA | NA | NA | IPR001128, IPR002401, IPR017972 | -2.03 | 0.94 | Down |
| *POX06407* | NA | NA | NA | NA | 1.97 | 0.93 | Up |
| *POX06410* | NA | NA | NA | NA | 5.49 | 0.99 | Up |
| *POX06413* | NA | NA | NA | IPR001452 | 1.42 | 0.89 | Up |
| *POX06418* | NA | NA | NA | NA | 2.32 | 0.82 | Up |
| *POX06426* | NA | NA | NA | IPR003663, IPR005828, IPR005829, IPR020846 | 1.11 | 0.83 | Up |
| *POX06450* | NA | NA | NA | IPR002198, IPR002347, IPR016040, IPR020904 | 1.49 | 0.81 | Up |
| *POX06471* | NA | NA | NA | IPR012535 | 1.90 | 0.91 | Up |
| *POX06473* | NA | NA | NA | IPR000092, IPR008949, IPR017446 | 1.28 | 0.90 | Up |
| *POX06478* | NA | NA | NA | NA | 3.91 | 0.95 | Up |
| *POX06500* | NA | NA | GT35 | IPR000811, IPR011833 | -1.74 | 0.94 | Down |
| *POX06513* | NA | NA | NA | IPR003495, IPR011629, IPR027417 | -1.99 | 0.96 | Down |
| *POX06525* | NA | NA | NA | NA | 2.88 | 0.95 | Up |
| *POX06526* | NA | NA | NA | IPR003445 | 2.65 | 0.94 | Up |

| | | | | | | | |
|---|---|---|---|---|---|---|---|
| *POX06528* | NA | NA | NA | NA | -3.95 | 0.95 | Down |
| *POX06534* | C2H2 zinc finger | NA | NA | IPR007087, IPR013087, IPR015880 | 4.00 | 0.97 | Up |
| *POX06541* | NA | NA | NA | IPR001544, IPR027417 | -1.92 | 0.86 | Down |
| *POX06549* | NA | NA | NA | IPR019931 | -3.22 | 0.93 | Down |
| *POX06583* | NA | NA | NA | IPR006139, IPR006140, IPR016040, IPR029752, IPR029753 | 1.52 | 0.88 | Up |
| *POX06590* | NA | NA | NA | IPR004045, IPR004046, IPR010987, IPR012336 | -1.18 | 0.87 | Down |
| *POX06595* | NA | NA | NA | IPR001338 | -1.12 | 0.93 | Down |
| *POX06599* | NA | β-xylosidase | CBM1; GH62 | IPR000254, IPR005193, IPR023296 | -2.09 | 0.95 | Down |
| *POX06600* | NA | β-xylosidase | CBM1; GH43 | IPR000254, IPR006710, IPR013320, IPR023296 | -1.89 | 0.87 | Down |
| *POX06601* | NA | Endo-β-1'4-xylanase | CBM1; GH30 | IPR000254, IPR001139, IPR013780, IPR013781, IPR017853 | -4.49 | 0.97 | Down |
| *POX06603* | NA | NA | NA | NA | -2.04 | 0.87 | Down |
| *POX06604* | NA | NA | NA | IPR005828, IPR020846 | 1.59 | 0.90 | Up |
| *POX06607* | NA | NA | NA | IPR030960 | 1.57 | 0.92 | Up |
| *POX06608* | NA | NA | NA | IPR001251, IPR011074 | 2.30 | 0.95 | Up |
| *POX06641* | NA | NA | NA | IPR011701, IPR020846 | 1.52 | 0.85 | Up |
| *POX06654* | NA | NA | NA | IPR020373 | -1.41 | 0.93 | Down |
| *POX06701* | Transcription factor TFIIS | NA | NA | IPR001222, IPR001529, IPR012164 | -1.17 | 0.85 | Down |
| *POX06738* | NA | NA | NA | IPR006823, IPR031329, IPR031331 | -1.55 | 0.88 | Down |
| *POX06740* | NA | NA | NA | NA | -2.19 | 0.83 | Down |
| *POX06743* | NA | NA | NA | IPR006328, IPR006439, IPR023198, IPR023214 | -1.61 | 0.92 | Down |
| *POX06754* | NA | NA | NA | NA | 3.10 | 0.92 | Up |
| *POX06755* | NA | NA | NA | IPR017981, IPR022596, IPR023041 | -1.25 | 0.81 | Down |
| *POX06768* | NA | NA | NA | IPR019236 | 2.06 | 0.92 | Up |
| *POX06772* | NA | NA | NA | NA | 1.62 | 0.90 | Up |
| *POX06783* | NA | Endo-β-1'4-xylanase | CBM1; GH11 | IPR000254, IPR001137, IPR013319, IPR013320, IPR018208 | -3.72 | 0.97 | Down |
| *POX06820* | NA | NA | NA | NA | -1.91 | 0.96 | Down |

| | | | | | | | |
|---|---|---|---|---|---|---|---|
| *POX06835* | NA | β-glucosidase | GH3 | IPR001764, IPR002772, IPR017853, IPR019800, IPR026891, IPR026892 | -1.78 | 0.92 | Down |
| *POX06891* | NA | NA | NA | IPR032710 | -1.67 | 0.84 | Down |
| *POX06899* | NA | NA | GH5 | IPR001547, IPR013781, IPR017853 | 5.89 | 0.98 | Up |
| *POX06900* | NA | NA | GH2 | IPR006102, IPR006103, IPR006104, IPR008979, IPR013781, IPR013812, IPR017853 | 3.66 | 0.94 | Up |
| *POX06901* | NA | NA | NA | IPR012292 | -4.80 | 0.97 | Down |
| *POX06904* | NA | NA | NA | IPR018466 | -1.66 | 0.95 | Down |
| *POX06915* | NA | NA | NA | IPR011701, IPR020846 | -1.07 | 0.87 | Down |
| *POX06921* | NA | NA | NA | NA | -2.89 | 0.92 | Down |
| *POX06951* | NA | NA | NA | IPR025331 | 4.07 | 0.98 | Up |
| *POX06952* | NA | NA | NA | IPR016040 | 3.53 | 0.82 | Up |
| *POX06959* | NA | NA | GH81 | IPR005200 | 3.12 | 0.94 | Up |
| *POX06963* | NA | NA | NA | NA | -3.45 | 0.95 | Down |
| *POX06964* | NA | NA | NA | NA | -3.39 | 0.97 | Down |
| *POX06983* | NA | Endo-β-1'4-glucanase | GH12 | IPR002594, IPR013319, IPR013320 | -3.04 | 0.93 | Down |
| *POX06986* | NA | NA | NA | IPR006671, IPR013763 | 2.03 | 0.89 | Up |
| *POX07003* | NA | NA | NA | IPR001841, IPR013083 | 9.68 | 0.88 | Up |
| *POX07025* | TEA/ATTS | NA | NA | IPR000818 | 4.62 | 0.95 | Up |
| *POX07033* | NA | NA | NA | IPR000262, IPR012133, IPR013785 | 3.24 | 0.95 | Up |
| *POX07037* | NA | NA | NA | IPR000048, IPR001715, IPR029955 | 1.72 | 0.85 | Up |
| *POX07052* | NA | NA | NA | IPR011701, IPR020846 | -1.52 | 0.85 | Down |
| *POX07073* | NA | NA | NA | NA | 4.13 | 0.82 | Up |
| *POX07075* | NA | NA | NA | NA | 1.07 | 0.83 | Up |
| *POX07081* | NA | NA | NA | IPR017946 | 2.01 | 0.95 | Up |
| *POX07083* | NA | NA | GH16 | IPR000757, IPR013320 | 3.15 | 0.94 | Up |
| *POX07099* | Myb | NA | NA | IPR001005, IPR009057, IPR017930 | 1.48 | 0.94 | Up |
| *POX07135* | NA | NA | NA | NA | 1.36 | 0.94 | Up |
| *POX07155* | NA | NA | NA | IPR002587, IPR013021, IPR016040 | -1.52 | 0.94 | Down |
| *POX07158* | NA | NA | NA | IPR002085, IPR011032, IPR013149, IPR013154, IPR016040, IPR020843 | -1.67 | 0.86 | Down |
| *POX07173* | NA | NA | NA | IPR025714, IPR029063 | -3.26 | 0.95 | Down |
| *POX07196* | NA | NA | NA | NA | 3.46 | 0.92 | Up |
| *POX07222* | NA | NA | NA | IPR000182, IPR016181 | 1.47 | 0.90 | Up |

| | | | | | | | |
|---|---|---|---|---|---|---|---|
| *POX07226* | NA | NA | NA | NA | 1.45 | 0.81 | Up |
| *POX07227* | NA | NA | NA | IPR003663, IPR005828, IPR005829, IPR020846 | 4.79 | 0.93 | Up |
| *POX07232* | NA | NA | NA | NA | 2.53 | 0.93 | Up |
| *POX07238* | NA | NA | NA | NA | 5.53 | 0.99 | Up |
| *POX07255* | NA | NA | NA | NA | -1.01 | 0.85 | Down |
| *POX07258* | NA | NA | NA | NA | 2.62 | 0.81 | Up |
| *POX07260* | NA | NA | NA | NA | 3.70 | 0.83 | Up |
| *POX07261* | NA | NA | NA | NA | 2.45 | 0.83 | Up |
| *POX07268* | NA | NA | NA | IPR005629, IPR013320 | 4.71 | 0.95 | Up |
| *POX07269* | NA | NA | NA | IPR005801, IPR015890, IPR019996 | 1.46 | 0.93 | Up |
| *POX07270* | NA | NA | NA | IPR025714, IPR029063 | 1.70 | 0.95 | Up |
| *POX07279* | NA | NA | NA | NA | 1.40 | 0.89 | Up |
| *POX07285* | NA | NA | GH5 | IPR001547, IPR013781, IPR017853 | 1.61 | 0.83 | Up |
| *POX07313* | NA | NA | NA | NA | 1.34 | 0.82 | Up |
| *POX07332* | NA | NA | NA | IPR011701, IPR020846 | 2.30 | 0.95 | Up |
| *POX07339* | NA | NA | NA | IPR009009, IPR010829 | 1.51 | 0.93 | Up |
| *POX07351* | NA | NA | NA | IPR001494, IPR011989, IPR016024, IPR021133 | -1.10 | 0.88 | Down |
| *POX07371* | NA | NA | NA | IPR000209, IPR009020, IPR015366, IPR030400 | -1.31 | 0.81 | Down |
| *POX07379* | NA | NA | NA | IPR008427, IPR014005 | 1.54 | 0.92 | Up |
| *POX07380* | NA | NA | GH25 | IPR002053, IPR008270, IPR013781, IPR017853, IPR018077 | 1.32 | 0.91 | Up |
| *POX07387* | NA | NA | NA | IPR011701, IPR020846 | -2.01 | 0.89 | Down |
| *POX07402* | NA | NA | NA | NA | 1.15 | 0.87 | Up |
| *POX07403* | NA | NA | NA | IPR006045, IPR011051, IPR014710, IPR017774 | 3.05 | 0.87 | Up |
| *POX07408* | NA | NA | NA | IPR002198, IPR002347, IPR016040 | -1.71 | 0.96 | Down |
| *POX07412* | NA | NA | NA | NA | 1.33 | 0.81 | Up |
| *POX07413* | NA | NA | NA | NA | 1.16 | 0.86 | Up |
| *POX07422* | NA | NA | NA | IPR002562, IPR012337 | -1.67 | 0.84 | Down |
| *POX07423* | NA | NA | CBM18 | IPR001002, IPR018392 | -2.43 | 0.89 | Down |
| *POX07424* | NA | NA | GH18 | IPR001223, IPR001579, IPR011583, IPR013781, IPR017853, IPR029070 | -2.06 | 0.88 | Down |
| *POX07428* | NA | NA | NA | IPR004648, IPR004813 | 2.72 | 0.91 | Up |

| | | | | | | | |
|---|---|---|---|---|---|---|---|
| *POX07432* | NA | NA | NA | IPR013216, IPR029063 | -1.31 | 0.93 | Down |
| *POX07451* | NA | NA | NA | IPR003663, IPR005828, IPR005829, IPR020846 | 2.98 | 0.87 | Up |
| *POX07466* | NA | NA | NA | IPR013087, IPR015880, IPR027775 | 1.13 | 0.85 | Up |
| *POX07470* | NA | NA | GH20 | IPR013781, IPR015883, IPR017853, IPR025705, IPR029018, IPR029019 | 1.76 | 0.89 | Up |
| *POX07477* | NA | NA | NA | IPR013226 | 2.26 | 0.92 | Up |
| *POX07482* | NA | NA | CBM50 | IPR018392 | -2.55 | 0.89 | Down |
| *POX07485* | NA | NA | NA | NA | -1.34 | 0.80 | Down |
| *POX07488* | NA | NA | NA | IPR025676 | -1.47 | 0.94 | Down |
| *POX07491* | NA | NA | NA | IPR018465 | -1.37 | 0.80 | Down |
| *POX07497* | NA | NA | NA | IPR003100, IPR003165, IPR012337, IPR014811, IPR032472, IPR032474 | -1.62 | 0.90 | Down |
| *POX07511* | NA | NA | NA | IPR018466 | 7.82 | 1.00 | Up |
| *POX07516* | NA | NA | NA | IPR003689, IPR004698 | -2.13 | 0.96 | Down |
| *POX07517* | NA | NA | NA | NA | -2.21 | 0.89 | Down |
| *POX07524* | NA | NA | GH78; CBM67 | IPR008902, IPR008928, IPR008979, IPR013737, IPR016007 | -1.20 | 0.85 | Down |
| *POX07532* | NA | NA | NA | NA | -4.48 | 0.98 | Down |
| *POX07534* | NA | NA | GH17 | IPR013781, IPR017853 | -1.97 | 0.96 | Down |
| *POX07535* | NA | Endo-β-1'4-glucanase | GH12 | IPR002594, IPR013319, IPR013320 | -2.41 | 0.94 | Down |
| *POX07539* | NA | NA | NA | IPR013057 | -2.05 | 0.92 | Down |
| *POX07545* | NA | NA | NA | IPR001357, IPR004102, IPR008893, IPR012317 | -1.11 | 0.88 | Down |
| *POX07576* | NA | NA | NA | IPR003663, IPR005828, IPR005829, IPR020846 | 3.99 | 0.96 | Up |
| *POX07584* | NA | NA | NA | IPR006045, IPR011051, IPR014710, IPR017774 | -1.65 | 0.95 | Down |
| *POX07594* | NA | NA | NA | NA | 7.00 | 1.00 | Up |
| *POX07603* | NA | NA | NA | NA | 2.06 | 0.81 | Up |
| *POX07609* | NA | NA | GT34 | IPR008630 | 2.86 | 0.86 | Up |
| *POX07618* | NA | NA | NA | IPR000182, IPR016181 | -1.66 | 0.85 | Down |
| *POX07619* | NA | NA | NA | IPR022124 | -4.26 | 0.97 | Down |
| *POX07620* | NA | NA | NA | NA | -2.98 | 0.92 | Down |
| *POX07621* | NA | NA | NA | IPR000571, IPR027417 | -7.33 | 0.94 | Down |

| | | | | | | | |
|---|---|---|---|---|---|---|---|
| *POX07622* | NA | NA | NA | NA | -6.24 | 0.97 | Down |
| *POX07639* | NA | NA | NA | IPR002198, IPR002347, IPR016040 | 2.61 | 0.91 | Up |
| *POX07641* | NA | NA | CBM24; GH71 | IPR000209, IPR005197, IPR015500 | -1.95 | 0.81 | Down |
| *POX07642* | NA | NA | NA | IPR001806, IPR027417 | -1.22 | 0.85 | Down |
| *POX07668* | NA | NA | NA | NA | 2.79 | 0.95 | Up |
| *POX07669* | NA | NA | NA | NA | 2.44 | 0.95 | Up |
| *POX07677* | NA | NA | NA | NA | -1.62 | 0.82 | Down |
| *POX07682* | NA | NA | NA | NA | 3.06 | 0.92 | Up |
| *POX07706* | NA | NA | NA | IPR007130 | 1.14 | 0.86 | Up |
| *POX07747* | Zn2Cys6 | NA | NA | IPR007219 | 1.08 | 0.84 | Up |
| *POX07751* | NA | NA | NA | NA | 6.38 | 0.93 | Up |
| *POX07772* | NA | NA | NA | IPR003609 | 1.48 | 0.84 | Up |
| *POX07785* | NA | NA | NA | NA | -2.51 | 0.82 | Down |
| *POX07819* | NA | NA | NA | NA | -1.38 | 0.83 | Down |
| *POX07830* | NA | NA | NA | NA | -1.11 | 0.92 | Down |
| *POX07845* | NA | NA | NA | NA | 3.53 | 0.94 | Up |
| *POX07861* | NA | NA | NA | NA | 1.23 | 0.82 | Up |
| *POX07865* | NA | NA | NA | IPR000873, IPR007817, IPR009081, IPR020845, IPR023213 | -1.71 | 0.95 | Down |
| *POX07866* | NA | NA | NA | IPR007274, IPR003480 | 2.58 | 0.82 | Up |
| *POX07871* | Zn2Cys6 | NA | NA | IPR001138, IPR007219 | -1.11 | 0.85 | Down |
| *POX07881* | NA | NA | NA | IPR002085, IPR011032, IPR013149, IPR013154, IPR016040, IPR020843 | 1.89 | 0.83 | Up |
| *POX07887* | NA | NA | NA | IPR027843, IPR032710 | -2.89 | 0.97 | Down |
| *POX07889* | NA | NA | NA | IPR002938, IPR023753 | -1.84 | 0.93 | Down |
| *POX07890* | NA | Pectate | PL1 | IPR002022, IPR011050, IPR012334 | 3.99 | 0.97 | Up |
| *POX07894* | NA | NA | NA | IPR010699 | 1.67 | 0.85 | Up |
| *POX07896* | NA | NA | NA | IPR015813, IPR018523 | 5.15 | 0.99 | Up |
| *POX07899* | NA | NA | NA | IPR000873, IPR025110 | -4.36 | 0.87 | Down |
| *POX07909* | NA | NA | NA | IPR008757, IPR024079 | 3.20 | 0.86 | Up |
| *POX07913* | NA | NA | NA | IPR011058 | 3.05 | 0.95 | Up |
| *POX07932* | NA | Pectin | CE8 | IPR000070, IPR011050, IPR012334, IPR018040 | -2.99 | 0.91 | Down |
| *POX07938* | Zn2Cys6 | NA | NA | IPR001138 | 1.16 | 0.93 | Up |
| *POX07948* | NA | NA | NA | IPR000719, IPR002290, IPR008271, IPR011009, IPR017441 | -9.35 | 0.90 | Down |

Journal of Fungi

MDPI

| | | | | | | | |
|---|---|---|---|---|---|---|---|
| *POX07949* | NA | NA | NA | NA | -1.79 | 0.92 | Down |
| *POX07962* | NA | NA | NA | IPR029068 | -1.46 | 0.93 | Down |
| *POX07963* | NA | β-glucosidase | GH3 | IPR001764, IPR002772, IPR017853, IPR019800, IPR026891, IPR026892 | 2.03 | 0.89 | Up |
| *POX07975* | NA | NA | NA | IPR000742, IPR013032 | -1.69 | 0.87 | Down |
| *POX07996* | NA | NA | NA | IPR007138, IPR011008 | 1.14 | 0.88 | Up |
| *POX08027* | NA | NA | NA | NA | 3.09 | 0.96 | Up |
| *POX08030* | NA | NA | NA | IPR004045, IPR010987, IPR012336 | -1.80 | 0.96 | Down |
| *POX08032* | NA | NA | NA | IPR016040 | 1.47 | 0.85 | Up |
| *POX08034* | NA | NA | GH114 | IPR004352, IPR017853 | -1.48 | 0.92 | Down |
| *POX08037* | NA | NA | NA | NA | -1.69 | 0.92 | Down |
| *POX08048* | NA | NA | NA | NA | 1.39 | 0.93 | Up |
| *POX08049* | NA | NA | NA | NA | 1.80 | 0.93 | Up |
| *POX08065* | NA | NA | NA | NA | -1.32 | 0.82 | Down |
| *POX08076* | NA | NA | GT71 | IPR022751, IPR029044 | 4.00 | 0.89 | Up |
| *POX08077* | NA | NA | GT34 | IPR008630 | 3.22 | 0.88 | Up |
| *POX08078* | NA | NA | NA | IPR002656 | 6.11 | 0.88 | Up |
| *POX08079* | NA | NA | NA | IPR001128, IPR001433, IPR002401, IPR003097, IPR008254, IPR017927, IPR017938, IPR017972, IPR023173, IPR029039 | 1.01 | 0.88 | Up |
| *POX08080* | NA | NA | GH16 | IPR000757, IPR013320 | -1.55 | 0.94 | Down |
| *POX08097* | C2H2 zinc finge | NA | NA | IPR007087, IPR013087, IPR015880 | -2.33 | 0.95 | Down |
| *POX08104* | NA | NA | NA | IPR004304 | -1.35 | 0.89 | Down |
| *POX08107* | NA | NA | NA | IPR000873, IPR003480, IPR020845, IPR023213, IPR025110, IPR032387 | 3.26 | 0.95 | Up |
| *POX08108* | NA | NA | NA | IPR008949, IPR024652 | 2.16 | 0.96 | Up |
| *POX08109* | NA | NA | NA | IPR000092, IPR008949 | 3.62 | 0.97 | Up |
| *POX08116* | NA | NA | NA | NA | 1.27 | 0.88 | Up |
| *POX08127* | NA | NA | NA | NA | 8.80 | 1.00 | Up |
| *POX08130* | NA | NA | NA | IPR021346 | -1.10 | 0.82 | Down |
| *POX08140* | NA | β-xylosidase | GH43 | IPR006710, IPR016840, IPR023296 | -2.96 | 0.92 | Down |
| *POX08154* | NA | NA | NA | NA | 1.75 | 0.92 | Up |
| *POX08157* | NA | NA | NA | NA | -3.15 | 0.85 | Down |

Journal of Fungi MDPI

| | | | | | | | |
|---|---|---|---|---|---|---|---|
| *POX08171* | NA | NA | GH18 | IPR001002, IPR001223, IPR001579, IPR011583, IPR013781, IPR017853, IPR018371 | 6.41 | 0.98 | Up |
| *POX08183* | NA | NA | NA | NA | -2.07 | 0.81 | Down |
| *POX08190* | NA | NA | NA | IPR008928, IPR014870, IPR032514 | 2.81 | 0.96 | Up |
| *POX08192* | NA | NA | NA | IPR018502 | 1.31 | 0.84 | Up |
| *POX08194* | NA | NA | NA | IPR009291 | 1.28 | 0.90 | Up |
| *POX08241* | NA | NA | NA | IPR003663, IPR005828, IPR005829, IPR020846 | -2.03 | 0.94 | Down |
| *POX08242* | NA | NA | NA | IPR002818, IPR029062 | -1.94 | 0.96 | Down |
| *POX08243* | NA | NA | NA | IPR001461, IPR001969, IPR021109 | -1.52 | 0.92 | Down |
| *POX08268* | NA | NA | NA | IPR001841, IPR002035, IPR031127 | -1.88 | 0.87 | Down |
| *POX08288* | NA | NA | NA | NA | 4.22 | 0.85 | Up |
| *POX08293* | NA | NA | NA | IPR025870, IPR029068 | 1.58 | 0.92 | Up |
| *POX08296* | NA | NA | NA | IPR002838, IPR016031 | -1.61 | 0.94 | Down |
| *POX08341* | NA | NA | NA | IPR000132, IPR003010 | -1.00 | 0.88 | Down |
| *POX08345* | NA | NA | NA | NA | 2.31 | 0.94 | Up |
| *POX08359* | NA | NA | NA | NA | -1.10 | 0.89 | Down |
| *POX08378* | NA | NA | NA | IPR012337, IPR013520 | 3.40 | 0.93 | Up |
| *POX08381* | NA | NA | NA | NA | 6.50 | 0.97 | Up |
| *POX08386* | NA | NA | NA | NA | 3.80 | 0.95 | Up |
| *POX08423* | NA | NA | NA | IPR010619, IPR024528 | 4.97 | 0.81 | Up |
| *POX08438* | NA | NA | NA | NA | -1.29 | 0.83 | Down |
| *POX08446* | NA | NA | NA | IPR011600, IPR029030 | -1.31 | 0.90 | Down |
| *POX08452* | NA | NA | NA | NA | 1.27 | 0.93 | Up |
| *POX08454* | NA | NA | NA | IPR008462 | -1.22 | 0.94 | Down |
| *POX08455* | NA | NA | NA | IPR000898 | 1.03 | 0.90 | Up |
| *POX08456* | NA | NA | NA | IPR002293 | 1.92 | 0.95 | Up |
| *POX08457* | NA | NA | NA | NA | 1.76 | 0.93 | Up |
| *POX08482* | NA | NA | NA | IPR024079, IPR029482 | 1.56 | 0.85 | Up |
| *POX08485* | NA | Expansin-like | CBM1 | IPR000254, IPR007112, IPR007117, IPR009009 | -1.39 | 0.94 | Down |
| *POX08486* | NA | NA | NA | NA | 1.09 | 0.93 | Up |
| *POX08497* | NA | NA | NA | NA | -1.25 | 0.92 | Down |
| *POX08579* | NA | NA | NA | NA | -1.40 | 0.90 | Down |
| *POX08596* | NA | NA | NA | IPR008427 | 7.15 | 1.00 | Up |

| | | | | | | | |
|---|---|---|---|---|---|---|---|
| *POX08602* | NA | NA | NA | IPR006913, IPR011057 | 3.13 | 0.81 | Up |
| *POX08613* | NA | NA | NA | IPR006680, IPR032466 | -1.92 | 0.90 | Down |
| *POX08617* | NA | NA | NA | NA | 1.59 | 0.87 | Up |
| *POX08627* | NA | NA | NA | NA | -3.85 | 0.91 | Down |
| *POX08628* | NA | NA | NA | IPR000626, IPR001841, IPR009060, IPR013083, IPR015940, IPR019103, IPR021109, IPR029071 | -1.41 | 0.93 | Down |
| *POX08633* | NA | NA | NA | IPR003819 | -2.74 | 0.83 | Down |
| *POX08636* | NA | NA | NA | IPR019623 | 4.10 | 0.96 | Up |
| *POX08656* | NA | NA | NA | IPR005829, IPR011701, IPR020846 | 6.04 | 0.93 | Up |
| *POX08659* | NA | NA | NA | IPR002655, IPR006091, IPR009075, IPR009100, IPR012258, IPR013786, IPR029320 | 2.63 | 0.95 | Up |
| *POX08664* | RFX DNA-binding domain | NA | NA | IPR003150, IPR011991 | 1.13 | 0.80 | Up |
| *POX08677* | NA | NA | NA | IPR018466 | -1.97 | 0.96 | Down |
| *POX08683* | NA | NA | NA | IPR021054 | 2.56 | 0.96 | Up |
| *POX08684* | NA | NA | NA | IPR006694 | 2.36 | 0.93 | Up |
| *POX08685* | NA | NA | NA | IPR005804, IPR015876 | 2.30 | 0.91 | Up |
| *POX08694* | NA | NA | AA1 | IPR001117, IPR002355, IPR008972, IPR011706, IPR011707, IPR017762 | 1.69 | 0.95 | Up |
| *POX08696* | NA | NA | NA | NA | 2.00 | 0.96 | Up |

Note: PFKM, fragments per kb per million reads.
